# Engineering Noble Metals Single-Atom Catalysts for Photothermal-Enhanced Photocatalytic Hydrogen Production

**DOI:** 10.1007/s40820-026-02298-2

**Published:** 2026-07-20

**Authors:** Luyu Zhou, Sixiang Liu, Lei Mou, Quan Xie, Wensheng Yang, Xiangyan Luo, Guolong Wu, Maosheng Ye, Wang Zhang, Jie Peng, Shuhui Sun, Junlong Tian

**Affiliations:** 1 Department of Electronic Science and Technology, Institute of Advanced Optoelectronic Materials and Technology, College of Big Data and Information Engineering, Guizhou University, Guiyang, 550025 People’s Republic of China; 2 Centre Énergie Matériaux Télécommunications, Institut National de La Recherche Scientifique (INRS), Varennes, QC J3X 1P7 Canada; 3 State Key Laboratory of Metal Matrix Composites, Shanghai Jiao Tong University, Shanghai, 200240 People’s Republic of China; 4 Laboratory for Quantum Engineering and Micro-Nano Energy Technology, School of Physics and Optoelectronic, Xiangtan University, Xiangtan, 411105 People’s Republic of China; 5 Guizhou Province Electronic Components and Devices Laboratory, Guiyang, 550016 People’s Republic of China

**Keywords:** Photothermal-enhanced photocatalysis, Photocatalytic hydrogen evolution, Single-atom catalysts, Noble metals, Operando thermometry

## Abstract

Photothermal-enhanced photocatalytic hydrogen evolution (PHE) converts nonradiative losses into localized interfacial heating, while precious-metal single-atom catalysts (SACs) offer atom-efficient and well-defined active sites. However, progress remains difficult to compare and translate because reaction-zone temperatures are often poorly constrained, the active forms of single atoms under light and heat are rarely verified, and performance in model suspensions does not readily extend to practical reactors or long-term operation. This Review summarizes major material platforms for photothermal harvesting and single-atom site design, and defines key support requirements, including strong light absorption, efficient charge and heat transport, and stable anchoring sites. It also outlines the typical roles of different noble metals and proposes actionable frameworks for the field. Thermometry-anchored protocols help disentangle thermal, photochemical, and synergistic effects through temperature-matched controls, absorption-normalized kinetics, and activation-energy benchmarks. An operando and ultrafast evidence framework tracks coordination, valence, charge transfer, and intermediates in real time, enabling verification of dynamic active sites. Finally, scale-relevant design rules connect photon and heat management with mass transport, bubble dynamics, scalable synthesis, and long-term validation, guiding photocatalysis toward mechanistically accountable and deployable solar H_2_ production.

## Highlights

- Defines support design rules for anchoring, charge extraction, heat management, mass transport, and durability in photocatalytic hydrogen evolution (PHE)-single-atom catalysts (SACs).
- Clarifies Pt, Ru/Ir, and Au/Ag functions as hydrogen evolution reaction centers, robust motifs, and plasmonic photothermal antennas.
- Summarizes key parameters and evidence for separating photochemical, photothermal, and coupled light–heat effects.
- Discusses stability, thermometry, long-term operation, and scale-up challenges for particulate PHE-SAC systems.

## Graphical abstract

**Figure:**
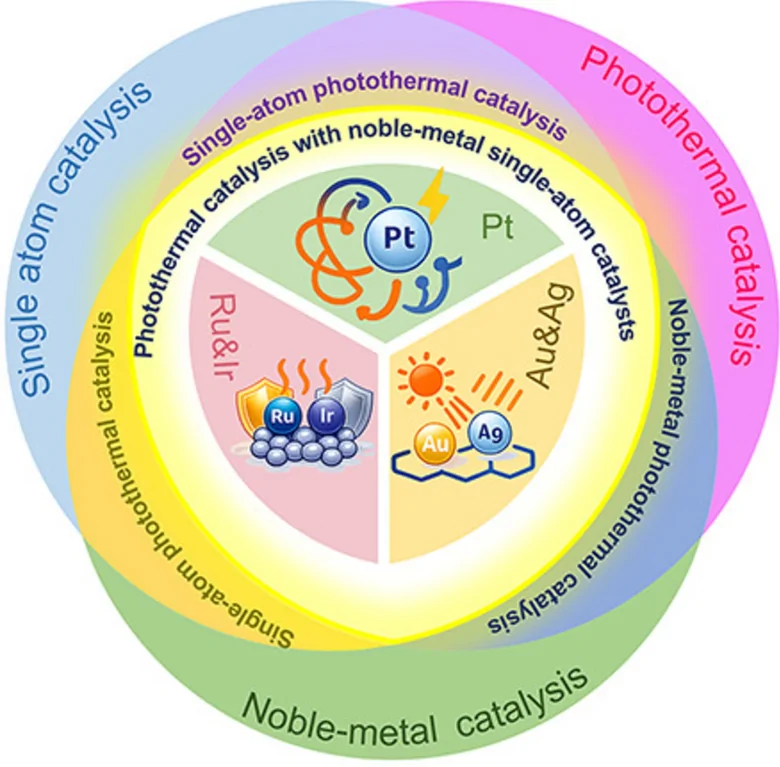

## Introduction

**Fig. 1:**
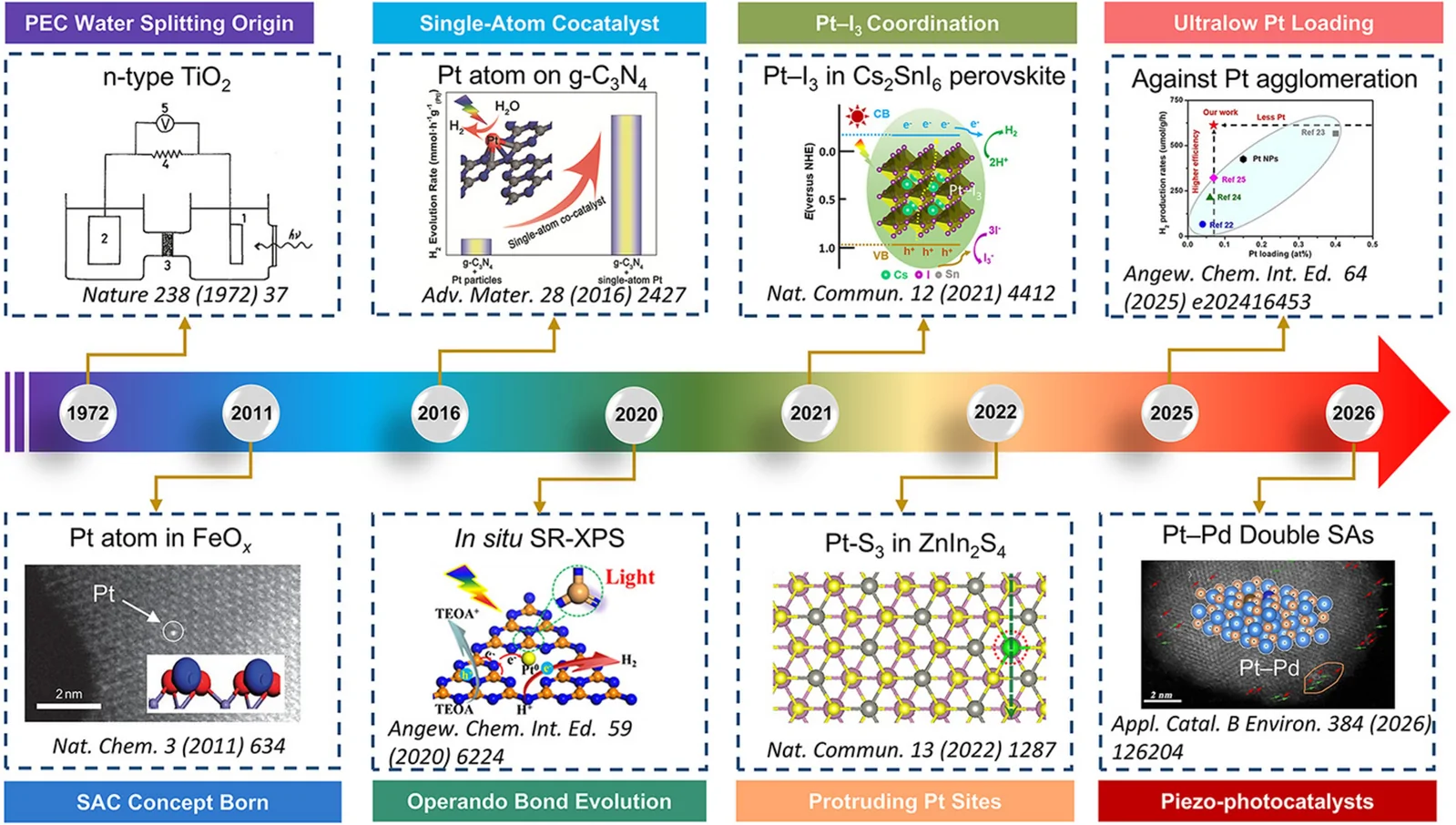
From the origin of water splitting to SACs-enabled photocatalytic H_2_ evolution

In 1972, Fujishima and Honda showed that light can drive water splitting on a semiconductor electrode, marking the starting point of solar-to-chemical hydrogen production (Fig. 1) [1]. Since then, photocatalysis has been viewed as a direct route to convert sunlight into chemical fuels for sustainable H_2_ generation. Among solar-to-fuel approaches, particulate photocatalysts for water splitting have attracted broad interest because of their simple device architecture, potentially low cost, and inherent scalability [2–5]. Recent progress at both the materials and system levels has pushed particulate and sheet platforms toward higher solar-to-hydrogen (STH) efficiency and scale-up validation. For example, STH efficiencies exceeding 9% have been reported together with outdoor demonstrations at the 100 m^2^ level, and particulate photocatalyst sheets have surpassed 1% STH, alongside system concepts that explicitly address practical gas handling and separation [4, 6–9]. Nevertheless, practical photocatalytic hydrogen evolution (PHE) is commonly limited by three coupled bottlenecks: (i) rapid recombination of photogenerated carriers, (ii) incomplete utilization of the solar spectrum, and (iii) sluggish surface reaction kinetics at the semiconductor–water interface, which often drives the system into a regime where surface kinetic and mass transport limitations become intertwined [2–5, 10, 11].

Compared with conventional thermocatalysis, which mainly relies on externally supplied bulk heat to overcome reaction barriers, photothermal-enhanced PHE offers a distinct light–heat–charge coupled route. In this route, absorbed photons generate electron–hole pairs for redox reactions, while part of the absorbed energy is converted into localized heat through nonradiative relaxation, defect-mediated recombination, or plasmonic decay. Such localized interfacial heating can accelerate adsorption/desorption, proton-coupled electron transfer, H_2_ desorption, and near-interface mass transport without necessarily heating the entire reaction medium to the same extent. Therefore, photothermal-enhanced PHE is not simply thermocatalysis under illumination, but a strategy to integrate solar light harvesting, charge separation, catalytic site activation, and heat management within one particulate catalyst system. This distinction provides the basis for developing photothermal-enhanced PHE, especially when atomic cocatalysts and engineered supports are used to couple electron delivery with local thermal activation.

In 2011, single atoms were established as genuine heterogeneous active centers with a clear evidence chain [12]. Since then, single-atom catalysts (SACs) have become a powerful option to tackle key limits in PHE. By placing isolated metal atoms at semiconductor interfaces, SACs maximize metal use at ultralow loadings and create more well-defined reaction sites [13–16]. Figure 1 summarizes how this field has progressed from early light-driven water splitting to the rise of atomically precise cocatalysts. Around 2016, noble-metal single atoms began to be used in photocatalytic H_2_ evolution as ultralow-loading electron sinks and as atomically defined hydrogen evolution reaction (HER) cocatalysts [17]. In many systems, these single atoms play two roles at once: They pull electrons away from the semiconductor to suppress recombination, and they act as catalytic centers for proton reduction. This dual function helps connect interfacial charge extraction with catalytic turnover in a more direct structure–function picture [18–28].

Typical examples include Pt single atoms hosted by covalent organic frameworks (COFs), immobilized on TiO_2_ through planar adsorption of Pt–porphyrin (Pt-TCPP), or stabilized on carbon nitride via Pt–N coordination, all of which allow control over site environment and location [19]. As the field matured, attention moved from simply making single atoms to engineering what the site is and where it sits: coordination and valence tuning to define the working site and improve stability [27], interface placement to shorten the electron-delivery path [25], and reconfigurable platforms that allow post-synthetic site programming under coupled stresses [28]. Such atomic-level engineering effectively modulates the electronic structure and interfacial charge-transfer dynamics, leading to pronounced enhancements in H_2_ evolution at ultralow Pt loadings [18–20, 23]. Mechanistic investigations further reveal that hybrid systems combining Pt single atoms with Pt clusters or other Pt species can exhibit synergistic dual-site behavior, providing insight into how distinct atomic ensembles partition charge collection and catalytic functions under illumination [21, 28]. Beyond Pt, pre-anchored Ru single atoms in red phosphorus exemplify how isolated metals can concurrently tailor defect chemistry and interfacial coupling, while oxygen-assisted stabilization of Au single atoms underscores the importance of preserving atomic dispersion under operating conditions.

Even when SACs improve interfacial charge extraction, many PHE systems still show pronounced surface kinetic limitations under realistic aqueous operation, particularly when *H adsorption or desorption, interfacial water reorganization, and near-interface mass transport become rate-determining [2–5, 29–31]. Meanwhile, a substantial fraction of absorbed solar energy is dissipated as heat via nonradiative relaxation. Photothermal-enhanced photocatalysis seeks to convert this dissipated energy into localized interfacial heating, thereby accelerating thermally activated elementary steps and improving transport near the reaction zone [32–36]. This coupling can be especially meaningful for SACs systems, because atomic sites can be placed at key charge-extraction regions, and their electronic structure and adsorption thermodynamics can be sensitive to modest temperature and solvation changes [15, 18, 32]. In this context, Au and Ag are frequently introduced as plasmonic antennas and effective nanoscale heaters to broaden spectral utilization and generate hotspots, whereas Pt, Ru, and Ir more commonly serve as atomic-scale reaction centers and electron sinks; the complementarity between “antenna” and “reactor” functionalities provides a structural basis for antenna–reactor-type designs [14, 18, 24, 37–43].

Constructing effective photothermal-enhanced SACs photocatalysts, therefore, requires coordinated optimization across multiple strongly coupled parameters. The support must stabilize isolated atoms in robust coordination pockets, enable rapid carrier extraction through favorable band alignment and strong electronic coupling, sustain controlled local heating in liquids, and maintain wettability and mass transport as temperature and solvent properties change [10, 11, 13–15, 31, 32, 35]. These requirements have motivated rapid progress across carbon nitrides, sulfides, oxides, and hybrid scaffolds, where different material families provide distinct levers including coordination chemistry, defect anchoring, interfacial fields, and photothermal response, to couple atomic-site chemistry with heat-assisted reactivity [11, 13, 15, 20, 23, 25, 31, 32, 34, 44–46]. For instance, carbon nitride can stabilize diverse single-atom cocatalysts and enable metal-dependent activity tuning, while ZnIn_2_S_4_- and CdS-based sulfides offer structurally tunable active motifs and rich interfacial coupling opportunities. TiO_2_-based platforms can promote stable single-atom anchoring and efficient charge extraction. Hybrid supports and heterostructure scaffolds further expand the design space by decoupling light harvesting, charge separation, and atomic-scale catalysis across multiple components, with interfaces serving as the central conduits for charge delivery to isolated metal sites [22, 47–56]. While such architectures enable precise control over coordination chemistry and charge-transfer pathways, their long-term durability under coupled optical, thermal, and chemical stresses remains a critical consideration.

To establish defensible structure and performance relationships, it is essential to rigorously distinguish thermal contributions from photochemical contributions. Early plasmonic and photothermal studies sometimes conflated localized heating with non-thermal hot-carrier effects, leading to ambiguous mechanistic interpretations [37, 41]. More recent work has advanced reproducible experimental frameworks, including temperature-matched dark controls, operando thermometry (or validated proxy thermometry), wavelength-resolved action spectroscopy, and Arrhenius-type kinetic analysis, to separate pure thermal acceleration from light-driven charge-transfer contributions and to quantify their relative importance [5, 37–42]. These tools further indicate that photothermal enhancement can originate from multiple channels, such as thermal acceleration of adsorption or desorption, temperature-dependent modulation of interfacial barriers and potentials, thermally induced interface restructuring, and mitigation of mass-transfer limitations including interfacial gas-evolution constraints [22, 35, 38–42, 46].

Operando and in situ characterization has become increasingly indispensable for connecting materials design to working mechanisms in SACs-enabled PHE and photothermal-enhanced systems. Synchrotron-based X-ray techniques provide element-specific access to coordination and oxidation-state evolution of single atoms under realistic conditions, while broader in situ*/operando* X-ray toolkits developed for photocatalysis offer transferable concepts for tracking active interfaces under illumination and bias [57–59]. For particulate systems, operando measurement strategies that infer effective catalyst potentials help bridge laboratory kinetics to system-relevant driving forces at the semiconductor–water interface [60]. Mechanistic attribution can be strengthened by direct operando identification of reactive electron species that actually drive HER under illumination, while electronic structure analyses can link frontier orbital alignment and carrier dynamics to the function of single-atom cocatalysts [61, 62]. Complementarily, modern kinetic frameworks for photocarrier transfer in catalysis and charge-carrier dynamics in organic photocatalysts provide essential benchmarks for judging whether photocarriers can reach SAC sites before recombination and whether near-interface transport truly limits observed rates [29, 30].

This Review is written for readers who want clear guidance on when photothermal-enhanced PHE truly works and how precious-metal single atoms can help make it work better. We begin with a short, practical overview of photothermal absorbers and single-atom site design, then distill what a good support looks like in real use, for example, strong light capture, fast charge and heat flow, and stable anchoring sites, and what different noble metals usually do in these systems. We then offer simple, usable ways to judge progress: how to measure and report the temperature where the reaction happens, how to use fair controls and basic kinetic checks to tell heat effects from light-driven chemistry, and how to verify the real working form of single atoms under light and heat rather than relying on static pictures. Finally, we connect these lessons to what matters for scale-up, including light and heat management, mass transport and bubble release, practical synthesis, and long-term testing.

## Design Principles of Support for Photothermal-Enhanced PHE

Photothermal-enhanced photocatalytic hydrogen evolution is not a simple extension of conventional photocatalysis, but a coupled process involving photon absorption, charge generation, nonradiative energy dissipation, localized heating, and surface proton-reduction kinetics. Under irradiation, semiconductor supports absorb photons to generate electron–hole pairs, while part of the absorbed energy is inevitably converted into heat through carrier relaxation, defect-mediated recombination, lattice vibration, or plasmonic decay. If this heat is generated near the catalytic interface, it can accelerate adsorption/desorption, proton-coupled electron transfer, H_2_ desorption, and local mass transport. Therefore, the design of supports in photothermal-enhanced PHE should consider not only light absorption and charge separation [11], but also photothermal conversion [63], heat localization, heat dissipation [64], and interfacial reaction accessibility [35].

### Functional Requirements of Semiconductor Supports

In aqueous single-atom photothermal-enhanced PHE, semiconductor supports are not merely hosts for noble-metal single atoms, but multifunctional platforms integrating light absorption, carrier generation, local heat production, charge extraction, and interfacial mass transport. Under irradiation, the support determines whether absorbed photons are converted into separated photocarriers, transformed into useful local heat, or lost through recombination and uncontrolled bulk heating. An effective support should, therefore, stabilize isolated atomic sites, deliver photocarriers to these sites before recombination, regulate heat localization and dissipation, and maintain access to water, protons, sacrificial donors, and H_2_ release pathways. Across inorganic semiconductors, polymer semiconductors, and COFs, these requirements can be summarized as atom trapping, energetic alignment, interfacial charge extraction, liquid-phase photothermal response, heat/mass transport, aqueous wettability, reaction-zone accessibility, and durability.

**Fig. 2:**
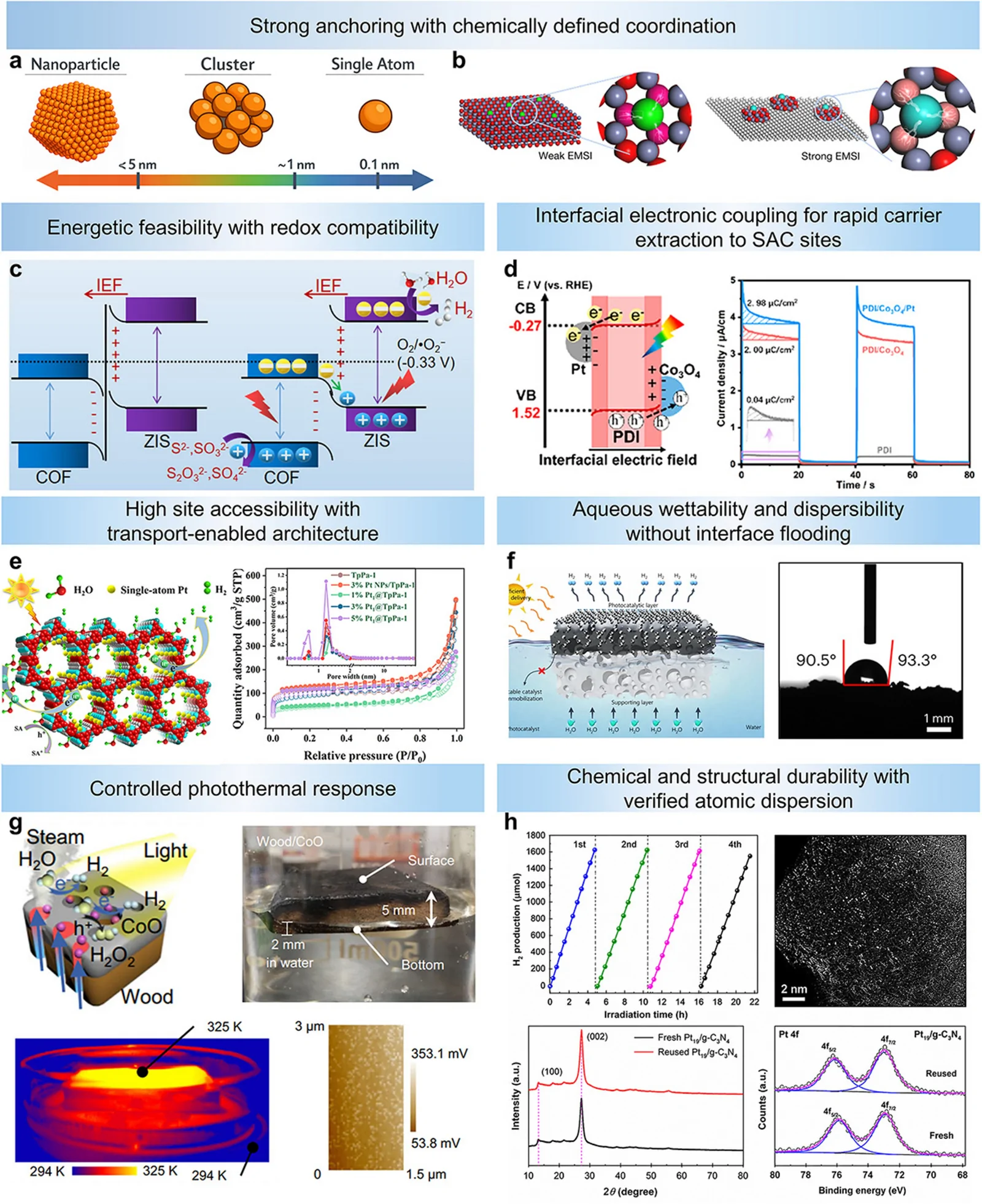
Support requirements for SACs photocatalysis. **a** Size regimes (single atom/cluster/nanoparticle) [65]. Copyright 2013, American Chemical Society. **b** Tunable EMSI via oxide band engineering [66]. Copyright 2025, Springer Nature. **c** S-scheme charge transfer [67]. Copyright 2024, Elsevier. **d** Interfacial coupling and charge density. [68] Copyright 2023, American Chemical Society. **e** Pt SA on TpPa-1-COF [69]. Copyright 2021, American Chemical Society. **f** Wettability and dispersion benefits [70]. Copyright 2023, Nature. **g** Photothermal interface heating [63]. Copyright 2021, Springer Nature. **h** Cycling durability and structural retention [23]. Copyright 2024, Royal Society of Chemistry

*Strong anchoring with chemically defined coordination.* In photothermal-enhanced PHE, local heating can promote not only surface reaction kinetics but also atom migration and coordination reconstruction. Therefore, chemically defined anchoring sites are required to keep noble-metal atoms isolated at the electron-delivery and HER-active interface. Compared with clusters (~ 1 nm) and nanoparticles (NPs) (< 5 nm), single atoms (~ 0.1 nm) are far more prone to migration and aggregation. Accordingly, supports must provide site-specific anchoring motifs, such as vacancies, heteroatoms, pore edges, or functional groups, to suppress atom migration and sintering (Fig. 2a, b) [65]. A primary requirement is robust, chemically defined coordination that stabilizes isolated atoms against migration and light-driven restructuring in water. For example, cation vacancies in Ti-deficient TiO_2_ can act as stable anchoring sites for Pt single atoms and mitigate the aggregation risks often associated with more labile vacancy environments under illumination [71–73]. A practical rule is to design strong binding pockets with high diffusion barriers and well-defined geometry (vacancies, heteroatom coordination, or confinement motifs) that preserve the intended single-atom coordination during operation. In practice, this requirement is most convincingly supported when the single-atom coordination remains unchanged in X-ray absorption near edge structure (XANES) and extended X-ray absorption fine structure (EXAFS) and isolated-atom contrast persists in high-angle annular dark-field scanning transmission electron microscopy (HAADF-STEM) after catalysis, with density functional theory (DFT) used to rationalize binding and migration barriers when available [74, 75]. Beyond geometric confinement, supports should enable chemically well-defined coordination environments (e.g., M–Nx, M–Ox, or mixed coordination), while their electronic structures should be adjustable to modulate the electronic metal–support interaction (EMSI). As schematically illustrated in Fig. 2b, tuning the oxide support (e.g., via size-dependent band-structure variation) provides a practical handle to regulate the charge state and local electronic structure of SAC sites, thereby optimizing adsorbate binding and activation while maintaining atomic dispersion [66]. Recent COF-based photo/electrocatalysts further support this principle, showing that structurally defined porphyrinic and metalated organic coordination motifs can stabilize metal centers while regulating their catalytic electronic structures [76, 77].

*Energetic feasibility with redox compatibility.* Photothermal heating can accelerate surface kinetics, but it cannot compensate for intrinsically unfavorable redox energetics. Therefore, the support must first provide suitable band positions or frontier energy levels for proton reduction and the corresponding oxidation reaction. The support must provide energy levels that thermodynamically enable proton reduction while remaining compatible with the chosen oxidation half-reaction (water oxidation or sacrificial donors). This requirement is often realized through deliberate band alignment and interfacial charge management architectures, where the relative positions of the conduction and valence bands (for inorganic semiconductors) or frontier energy levels (for organic and polymer semiconductors) are tuned via composition control and donor–acceptor engineering to provide sufficient driving force. In particular, beyond simple type-II alignment, S-scheme (or related step-scheme) heterostructures can leverage interfacial band bending and built-in electric fields to drive charge redistribution such that weakly reactive carriers recombine across the interface, while high-energy electrons and holes are selectively retained on their respective components, thereby maintaining strong reduction and oxidation capability under illumination. Energetic feasibility must be evaluated together with carrier lifetime, because favorable alignment alone is insufficient if photocarriers are rapidly lost to recombination [67, 78, 79]. Accordingly, studies typically substantiate this point by reporting band-edge or flat-band values from Mott–Schottky analysis alongside optical gaps, then linking the “usable” driving force to wavelength-resolved quantum yields or carrier lifetime evidence rather than alignment alone (Fig. 2c) [67, 80].

For SAC-containing heterostructures, the role of an S-scheme junction should be further connected to the atomic catalytic outlet. The S-scheme interface can preserve high-energy electrons on the reductive component, but these electrons must still be extracted by isolated metal sites before recombination. In this sense, SACs serve as atomically defined electron sinks and proton-reduction centers that convert the charge-separation advantage of the S-scheme into measurable HER kinetics. Therefore, the effectiveness of S-scheme/SAC architectures should be evaluated not only by band alignment, but also by direct evidence of directional charge transfer from the reductive semiconductor component to SAC sites, such as transient photocurrent, time-resolved spectroscopy, in situ irradiated XPS, EPR, KPFM, or operando tracking of metal valence and coordination.

*Interfacial electronic coupling for rapid carrier extraction to SAC sites.* Local heating becomes productive only when thermally promoted surface steps are supplied with electrons at a comparable rate. Therefore, beyond favorable band positions, the support–SAC interface must rapidly deliver photogenerated electrons to isolated metal sites before recombination, while suppressing back-transfer. This requires intimate atomic-scale contact and sufficiently strong electronic coupling to enable fast electron injection or tunneling, while keeping the defect density under control so that trap-assisted recombination does not dominate (Fig. 2d) [68]. This function is critical because SAC sites act as efficient electron sinks and catalytic outlets. Accordingly, supports must not only generate photocarriers, but also deliver them rapidly to single-atom centers for proton reduction [61, 73, 81]. This role is commonly evidenced by reduced electrochemical impedance spectroscopy (EIS) resistance, enhanced transient photocurrent or photovoltage responses, and time-resolved spectroscopic signatures that distinguish interfacial electron transfer from recombination [81, 82]. For S-scheme/SAC systems, complementary evidence such as in situ irradiated XPS, EPR, KPFM, or operando tracking of metal valence and coordination is especially useful for confirming directional charge transfer from the reductive semiconductor component to isolated metal sites.

*High site accessibility with transport-enabled architecture.* When photothermal effects accelerate proton reduction and H_2_ desorption, the bottleneck can shift from intrinsic surface kinetics to reactant supply and product removal. Therefore, supports should expose a high density of accessible anchoring sites and provide efficient transport pathways for water, ions, sacrificial donors, and product gases near the catalytic interface. A clear structural principle is provided by hierarchical porous COFs, where introducing macropores into a microporous framework, namely, macro-TpBpy, increased the measured H_2_ evolution rate to 4.88 mmol g^−1^ h^−1^, about four times higher than its microporous analog, consistent with improved mass transport and light penetration (Fig. 2e) [83]. Notably, this PHE system relied on a Pt cocatalyst and a sacrificial electron donor, so it primarily illustrates how pore architecture can relieve transport resistance and bubble blockage once interfacial kinetics become fast. The same transport constraint may become even more decisive in SAC-based systems when photothermal contributions accelerate surface steps, because thermally promoted surface dynamics can shift the rate limitation toward mass transfer. Accordingly, claims regarding accessibility and transport are most defensible when pore metrics, such as surface area and hierarchical pore size distribution, are correlated with kinetic trends consistent with transport relief, as exemplified by macro-TpBpy-type hierarchical COFs and related morphology-controlled porous COF systems [83–86].

*Aqueous wettability and dispersibility without interface flooding.* In liquid-phase PHE, the solid–liquid interface determines whether local heating, proton supply, and H_2_ release can be sustained at catalytic sites. Therefore, support wettability should maintain water access while avoiding bubble pinning, excessive swelling, or interfacial flooding. A well-wetted and well-dispersed support can maximize effective photon utilization and sustain continuous water contact with catalytic sites [87]. This requirement is not merely a materials property but an interfacial transport function: Properly tuned surfaces enhance reactant access, facilitate heat exchange with the surrounding liquid, and minimize bubble adhesion, thereby keeping active sites available under irradiation (Fig. 2f) [70]. For polymer photocatalysts, inserting hydrophilic non-conjugated segments into the main chain can improve water–polymer interfacial contact and enable high apparent quantum yields under visible light, for example, 17.82% at 460 nm in a representative system [80]. Practically, wettability should be balanced because excessive swelling or interfacial flooding can slow H_2_ evacuation, weaken local thermal gradients, and hinder interface renewal [88]. Operationally, this balance is often reflected in strengthened water–material interactions. Where reported, it can be captured by contact angle measurements or water uptake characterization, and it commonly coincides with higher AQY under defined aqueous conditions, as observed in polymer systems incorporating hydrophilic segments [80].

*Controlled photothermal response.* The defining feature of photothermal-enhanced PHE is that part of the absorbed photon energy is converted into useful local heat rather than merely lost through nonradiative dissipation. Therefore, supports should provide a controlled photothermal response near the reaction zone instead of simply increasing the bulk temperature. In aqueous media, localized and moderate interfacial heating can accelerate elementary steps and improve interfacial transport, but excessive heat accumulation may destabilize the semiconductor, alter single-atom coordination, or increase recombination. Photothermally induced biphase concepts illustrate how localized interfacial heating can lower gas escape resistance and reshape mass-transfer limitations by creating a more transport favorable reaction environment. For support design, this means that photothermal functionality should be introduced into the support or coupled to the support in a way that broadens solar spectrum utilization, converts absorbed energy into productive local heating, and keeps photocarrier losses under control [35, 89]. Mechanistically, photothermal contributions are most credible when interfacial temperature is measured with calibrated micro- or nanoscale thermometry and when matched dark heating or light filter controls support a kinetic interpretation under comparable temperatures (Fig. 2g) [63, 64, 87].

*Chemical and structural durability with verified atomic dispersion.* Photothermal operation exposes supports and SAC sites to coupled photon, thermal, chemical, and mass transport stresses. Therefore, durability should be evaluated by both sustained activity and preservation of the working support–SAC structure. The support must resist photocorrosion, dissolution, framework collapse or swelling for porous organics, loss of porosity or crystallinity for COFs, and surface reconstruction that destroys anchoring motifs or electronic coupling [14, 90, 91]. This requirement is particularly stringent in SAC-based systems, because stable H_2_ evolution should coincide with preserved atomic dispersion and coordination rather than being inferred from activity retention alone (Fig. 2h) [23]. Accordingly, robust studies should couple long-term rate retention with structural verification of single-atom coordination, such as X-ray absorption fine structure and microscopy, as well as integrity checks of the support framework, such as powder X-ray diffraction and porosity retention [74, 75, 79, 92, 93].

Under photothermal PHE conditions, SAC stability is a dynamic property governed by coupled light, heat, charge, and mass transport. Local photothermal gradients may amplify deactivation pathways such as thermally assisted diffusion, atom migration, transient clustering, and irreversible sintering, while photogenerated charges, adsorbates, pH variations, solvent effects, and bubble evolution may alter the valence and coordination of isolated metal sites. Therefore, stable H_2_ evolution alone cannot prove SAC stability unless atomic dispersion and coordination are verified after, or preferably during, operation.

A universal temperature or photon flux threshold for SAC destabilization is not yet available, because stability depends on metal identity, support chemistry, coordination geometry, anchoring site density, metal loading, reaction medium, illumination spectrum, local temperature, and operation time. Future studies should report system-specific stability windows, including bulk/local temperatures, irradiance or photon flux, metal loading, reaction duration, and structural evidence before and after illumination. Representative stabilization strategies and material examples are summarized in Table 1.

**Table 1:** Stabilization strategies for SACs under photothermal-relevant conditions

Stabilization strategy	Substrate materials in three representative research articles	Single-atom metal species	Main stabilizing effect	Photothermal relevance	Key evidence
Vacancy/defect engineering	Ni(OH)ₓ with Ni^2^⁺ vacancies [94]	Pt	Vacancy/defect sites provide strong anchoring and increase migration barriers	Helps suppress thermally assisted atom migration and aggregation near local hotspots	HAADF-STEM, XANES/EXAFS, DFT binding energy, absence of M–M coordination
	Defective CoOOH with oxygen vacancies or threefold hollow sites [95]	Ir			
	Oxygen vacancy-rich Fe_2_O_3_ nanosheets [96]	Pt			
Coordination environment design	P/N-coordinated carbon support in Co–P_2_N_2_–C [97]	Co	Tailored M–N, M–P/N, or M–Nₓ coordination stabilizes local geometry and tunes electronic structure	Reduces detachment, valence drift, and coordination reconstruction during light–heat cycling	XANES/EXAFS coordination number, XPS valence, DFT charge distribution, operando XAS when available
	Mesoporous graphitic carbon nitride CNₓ [98]	Pd			
	Graphitic carbon nitride g-C_3_N_4_ [99]	Pd/Pt			
Spatial confinement	N/S/F-co-doped graphitized carbon [100]	Fe/Co/Ru/Ir/Pt	Pores, multilayers, or molecular cavities physically isolate metal atoms and limit atom–atom encounter	Restrains clustering and sintering under local heating, especially at higher loading	Pore/cage structure, HAADF-STEM statistics, EXAFS absence of M–M bonds, long-term cycling
	Pore-confined framework structures [101, 102]	Pt/Ir/Au/Ru			
	Porphyrinic MOF [103]	Pt			
Strong metal–support interaction	CeO_2_ [104]	Pt	Strong oxide anchoring and interfacial electronic coupling immobilize isolated atoms	Improves resistance to local thermal stress and suppresses thermally activated migration	High-temperature treatment stability, XAS coordination, Pt–O/Pt–Ce environment, post-reaction STEM

### Carbon Nitride Supports Including g-C_3_N_4_ Derivatives

Carbon nitrides are widely used supports for single-atom photothermal catalysts because their high specific surface area can expose abundant reaction interfaces, shorten carrier transport distances, and facilitate the transport of water, sacrificial agents, and product gases near catalytic sites. These features provide a structural basis for rapid interfacial reactions under photothermal-enhanced conditions. Moreover, their nitrogen-rich covalent frameworks provide abundant coordination sites, which can stabilize isolated metal atoms while maintaining visible-light-responsive activity. Beyond serving as passive hosts, recent studies increasingly demonstrate that the coordination microenvironment of carbon nitride actively reshapes interfacial charge-transfer kinetics [13, 20, 105–110]. In parallel, dopant and defect co-engineering on nitrogen-rich g-C_3_N_4_ further shows that local electron density and proton delivery can be tuned at the support level [105].

**Fig. 3:**
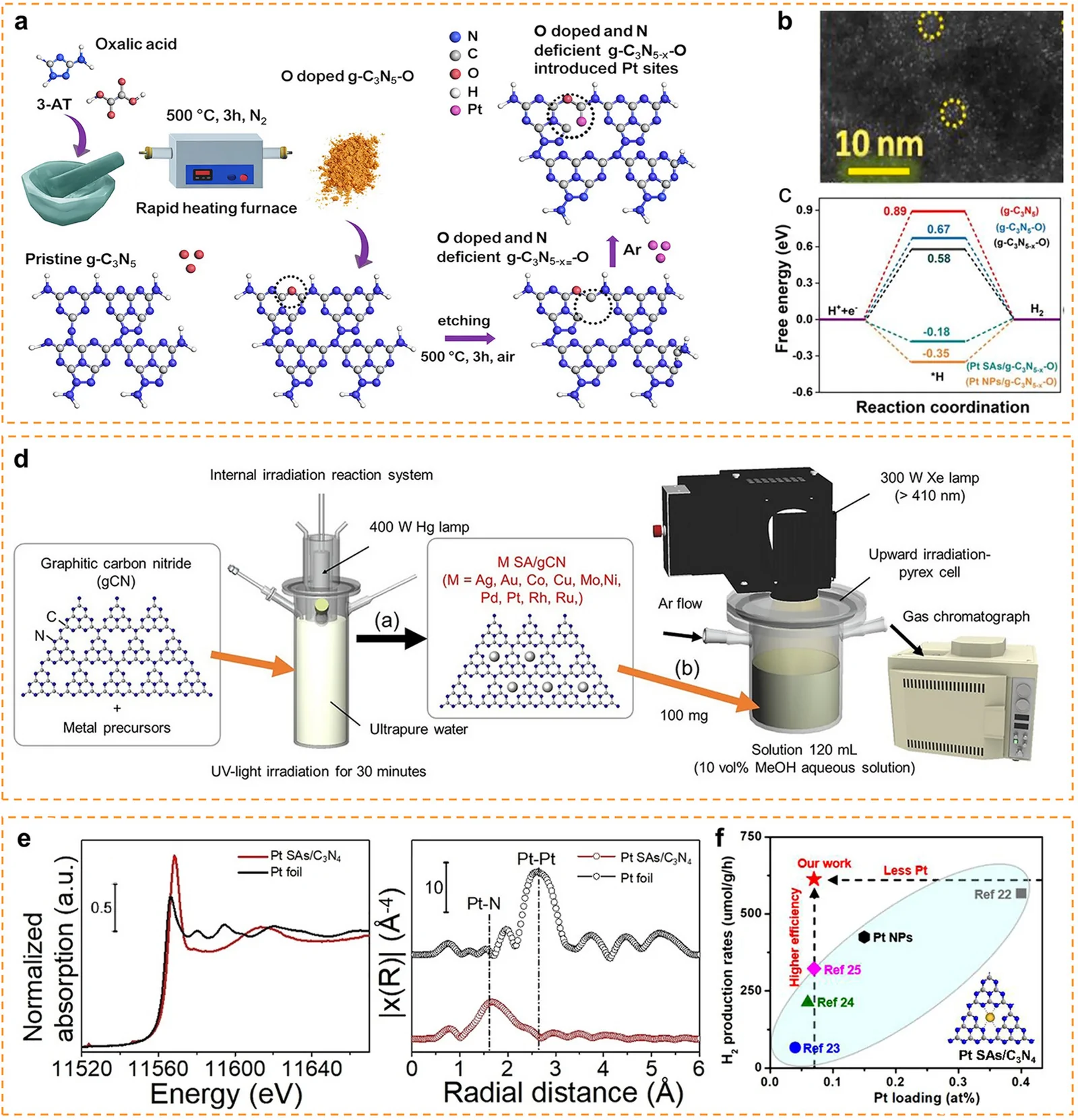
Single-atom engineering and support-level regulation in carbon nitride photocatalysts. **a** Synthesis of Pt single atoms on O-doped/N-vacancy g-C_3_N_5_. **b** HAADF-STEM confirms isolated Pt atoms (10 nm). **c** DFT ΔGH* shows optimized HER thermodynamics via defects and doping and Pt sites [105]. Copyright 2025, Elsevier. **d** Photodeposition route and H_2_-testing workflow on g-C_3_N_4_ [13]. Copyright 2023, Wiley–VCH. **e** Normalized XANES spectra at the Pt L_3_ edge and Fourier transform EXAFS spectra (R-space) of Pt SAs/C_3_N_4_. **f** Comparison of H_2_ production rate as a function of Pt loading [20]. Copyright 2024, Wiley–VCH

For example, Zhang et al. anchored Pt single atoms on O doped g-C_3_N_5_ [105]. They used a two-step thermal strategy to introduce O dopants and then create N defects, followed by Pt anchoring on g-C_3_N_5_ (Fig. 3a-c) [105]. The HAADF-STEM image in Fig. 3b evidences atomically dispersed Pt without detectable NPs, indicating effective anchoring of Pt onto the defect and dopant-engineered g-C_3_N_5_ matrix. Consistently, the DFT results in Fig. 3c show that the Pt–(O-doped, N-vacancy) local environment optimizes the hydrogen adsorption thermodynamics, thereby facilitating the key surface H* formation and desorption steps and promoting efficient H_2_ evolution.

The resulting site tuned intermediate adsorption. The photocatalyst delivered an H_2_ production rate of 1809.2 μmol g^−1^ h^−1^ under visible light [105]. This work shows how defect and dopant co-engineering can turn carbon nitride into an active pocket support, not a passive scaffold. A key next step is to test other metals on the same CN pocket design and clarify how metal identity changes the mechanism and stability. Akinaga et al. provided a metal species benchmark on g-C_3_N_4_ via photo-deposition (Fig. 3d). They irradiated g-C_3_N_4_ with metal precursors using a 400 W Hg lamp for 30 min, without sacrificial reagent, to weaken photoreduction and suppress aggregation [13]. This enabled single-atom loadings near 3 wt% for Cu, Ni, Pd, Pt, Rh, and Ru, while Au and Ag aggregated more easily. Pd single atoms showed 8.6 times higher H_2_ evolution rate per active site than Pd NPs [13]. The study set a practical synthesis and screening protocol. The main limitation is that most activity tests used methanol as a hole scavenger, while the surface reaction steps remain insufficiently resolved. From the perspective of metal utilization, Lazaar et al. further demonstrated that minimizing Pt single-atom loading on C_3_N_4_ can maximize photocatalytic H_2_ production efficiency. XANES and EXAFS confirmed Pt–N coordination without Pt–Pt scattering, indicating atomically dispersed Pt sites on the C_3_N_4_ framework (Fig. 3e). The resulting Pt SAs/C_3_N_4_ achieved higher H_2_ production efficiency at lower Pt loading than Pt nanoparticles and previously reported Pt/C_3_N_4_ systems, highlighting that efficient carbon nitride support design depends on placing isolated Pt atoms at electronically active coordination sites rather than simply increasing metal loading (Fig. 3f) [20].

Table 2 shows that carbon nitride-based single-atom photocatalysts mainly rely on nitrogen-rich frameworks to stabilize isolated metal sites and promote charge separation through metal–N coordination. Pt single atoms generally deliver higher H_2_ evolution rates and AQY values, confirming their advantages in electron trapping and proton reduction. Notably, most systems use TEOA as the sacrificial agent, usually at 10–20 vol%, while a few cases employ methanol. Overall, carbon nitride provides a tunable platform for SAC photocatalytic H_2_ evolution, and the combination of suitable metal sites with optimized coordination environments is essential for improving catalytic performance.

**Table 2:** Research status of single-atom photocatalysts supported on carbon nitride for hydrogen production

Support	Single-Atom Site / Coordination	Catalyst amount (mg)	Sacrificial agent	Light Irradiation HER	H_2_ Evolution Performance (mmol g^−1^ h^−1^)	AQY/AQE (nm)	Year
N-rich g-C_3_N_4_ (N–CN_x_)	Pt_1_–N_4_	10	10%TEOA, 50 mL	128.3 mW cm^−2^/ IR cutoff	30.0	21.3% (400) 15.9% (365) 8.2% (450) 1.4% (550)	2025 [111]
O substitution doping N_2c_	Pt–N_4_/Pt–N_2_/Pt–N_3_O	2	10%TEOA, 20 mL	285 mW cm^−2^/ full-spectrum irradiation	66.4	26.44% (390) 24.3% (365) 1.7% (450)	2025 [112]
N-rich C_3_N_4_	Pt single atoms	6	10%TEOA, 30 mL	UV cut-off	64.1	25.3% (420)	2025 [113]
Polymeric carbon nitride (PCN)	Rh single atoms	50	10%TEOA, 100 mL	*λ* > 420 nm	3.41	14.9% (420)	2025 [114]
g-C_3_N_4_	Pt single atoms (CD-stabilized)	10	20%TEOA, 100 mL	0.38 W cm^−2^ / AM 1.5	4.54	4.5% (420)	2024 [115]
Porous carbon nitride (PCN)	Cu single atoms	10	15% TEOA, 80 mL	λ > 420 nm	2.14	19.3% (420)	2023 [116]
P-doped carbon nitride	Co and Ag dual single atoms	20	10%TEOA, 80 mL	*λ* > 420 nm	1.19	1.49% (365)	2023[117]
g-C_3_N_4_	Pd single atoms	100	10% Methanol, 108 mL	*λ* > 420 nm	0.065	–	2023 [13]
g-C_3_N_4_	Cu single atom	20	10%TEOA, 100 mL	200 mW cm^–2^/ λ > 420 nm	11.23	66.38% (365) 31.60% (420) 11.87% (450) 3.92% (550)	2023 [118]
5-bromouracil-derived polymeric carbon nitride	Pt	40	10%TEOA, 40 mL	100 mW cm^–2^/ *λ* > 420 nm	0.563	2.62% (420)	2024 [119]
Ultrathin g-C_3_N_4_	Pt	100	10%TEOA, 10 mL	100 mW cm^–2^/ λ > 420 nm	1.13	15.20% (420)	2023 [120]
g-C_3_N_4_	Wu	10	10% TEOA	*λ* > 420 nm	3.02	4.11% (420)	2021 [121]
g-C_3_N_4_	Mo	10	10% TEOA, 50 mL	*λ* > 420 nm	4.86	4.88% (420)	2023 [122]

### Sulfide Supports Including CdS and ZnIn_2_S_4_

Metal sulfides such as CdS and ZnIn_2_S_4_ can serve as supports for single-atom photothermal catalysts because their suitable bandgaps enable visible-light absorption, while their rich surface chemistry provides anchoring sites for isolated metal atoms. In photothermal-enhanced PHE, their strong light absorption can further support local heat generation and promote interfacial reaction kinetics. However, carrier recombination and photocorrosion remain major bottlenecks, and these problems may be further aggravated when local heating promotes sulfur-vacancy evolution or surface reconstruction. Single-atom cocatalysts offer a direct way to address these issues. They maximize noble-metal efficiency and create strong EMSI that accelerates interfacial charge extraction [126]. Recent photothermal-oriented discussions further emphasize that isolated sites are also advantageous for stabilizing dynamic surface states of CdS under light and heat, provided that coordination and anchoring are well designed [123–125].

**Fig. 4:**
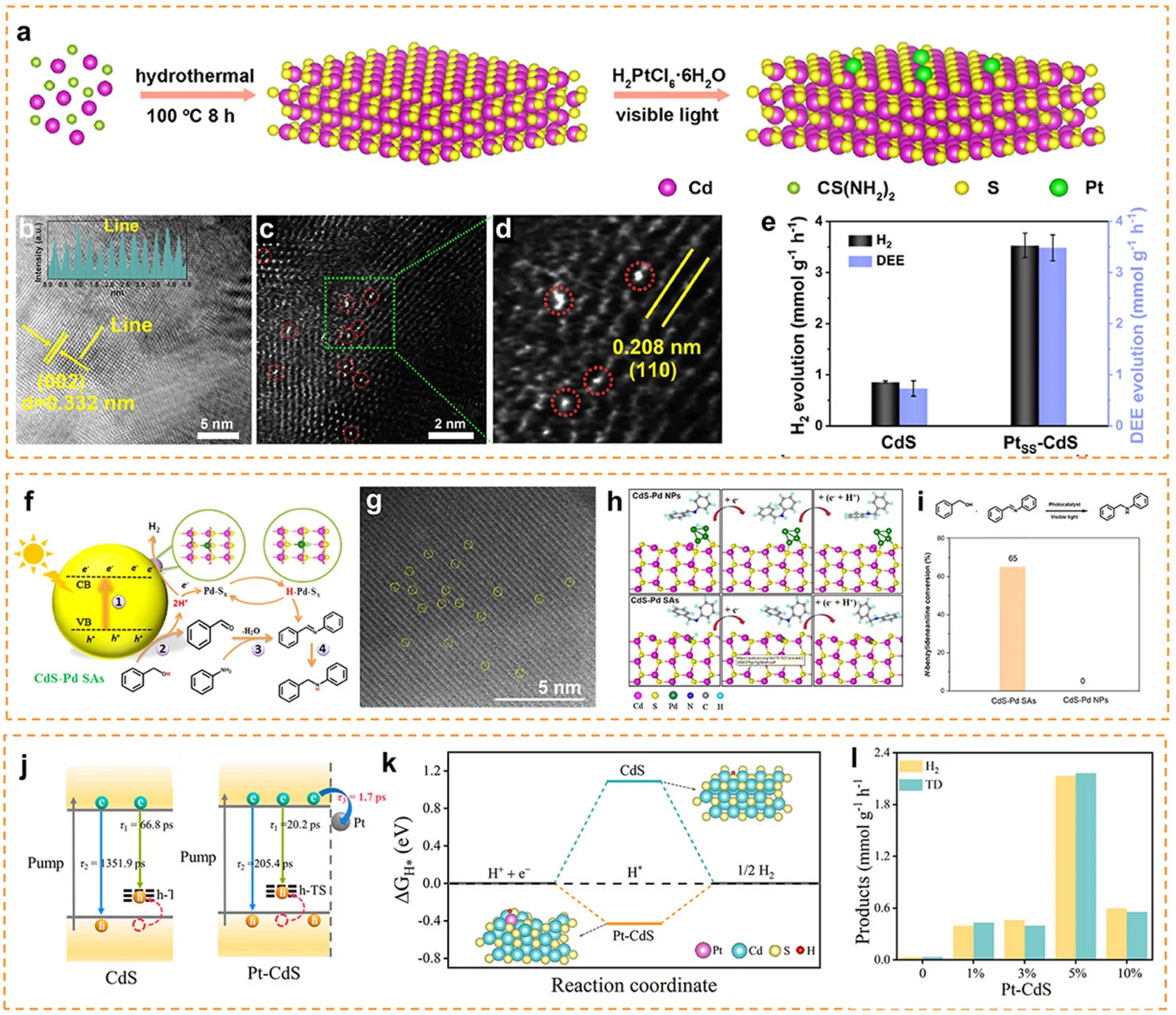
Single-atom Pt engineered on CdS regulates coordination, charge transfer, and H_2_-evolution pathways. **a** Scheme illustrating the fabrication of Pt_SS_-CdS. **b** HRTEM images of Pt_SS_-CdS. **c**, **d** HAADF-STEM images of Pt_SS_-CdS. **e** H_2_ evolution from ethanol under 420 nm [123]. Copyright 2023, American Chemical Society. **f** Schematic illustration of the mechanism for photocatalytic. **g** HAADF-STEM images of CdS−Pd SAs. **h** Hydrogenation reaction pathway illustration on the models of CdS−Pd NPs and CdS−Pd SAs.** i** Model reactions illustrating the strong hydrogen autotransfer ability of CdS−Pd NPs and CdS−Pd SAs [124]. Copyright 2022, American Chemical Society. **j** Ultrafast dynamics show faster electron extraction and reduced recombination. **k** Favorable HER free-energy profile on Pt–CdS. **l** H_2_/TD formation *vs.* Pt loading [125]. Copyright 2023, Elsevier

Recent CdS-based single-atom photocatalysts further demonstrate the importance of metal–S coordination in coupling H_2_ evolution with selective organic transformation. Jiang et al. constructed Pt single sites on two-dimensional CdS nanosheets, where isolated Pt atoms were coordinated with three surface S atoms to form Pt–S_3_ motifs (Fig. 4a-e) [123]. The Pt–S coordination promoted electron transfer from CdS to Pt and optimized H adsorption, leading to a H_2_ evolution rate of 11.5 mmol g^–1^ h^–1^ under simulated solar light with highly selective 1,1-diethoxyethane production. In another example, Niu et al. reported in situ coordinated Pd–Sx single sites on CdS for visible-light-driven C–N coupling with simultaneous H_2_ production (Fig. 4f-i) [124]. The Pd–Sx sites trapped photogenerated electrons, prolonged carrier lifetime, and served as hydrogen-transfer shuttles, achieving a H_2_ evolution rate of 11.8 mmol g^–1^ h^–1^. These studies indicate that atomically dispersed Pt–S and Pd–Sx motifs on CdS can act as electron extraction and hydrogen-transfer centers, thereby improving both proton reduction and coupled organic conversion.

Compared to the Pt–N_3_–CdS system, which focused on local coordination chemistry, this work demonstrated that extended defect engineering can provide dense anchoring sites and internal fields for directional charge flow, suggesting that morphology control and defect topology can be as decisive as chemical coordination. To further reveal the kinetic origin of charge utilization, Xiang et al. integrated atomically dispersed Pt on CdS quantum dots via a one-pot solvothermal deposition method (Fig. 4j-l) [125]. Ultrafast transient absorption spectroscopy confirmed electron transfer from CdS to Pt within 1.7 ps, greatly suppressing recombination [125]. The resulting Pt–CdS QDs exhibited a 70-fold enhancement in H_2_ generation compared to bare CdS and achieved 95.3% conversion and 87.2% selectivity in the oxidation of 2-thiophene methanol to 2-thiophenecarboxaldehyde [125]. This work complemented the previous two by establishing that rapid charge extraction at the atomic interface is equally critical as structural or coordination optimization.

**Fig. 5:**
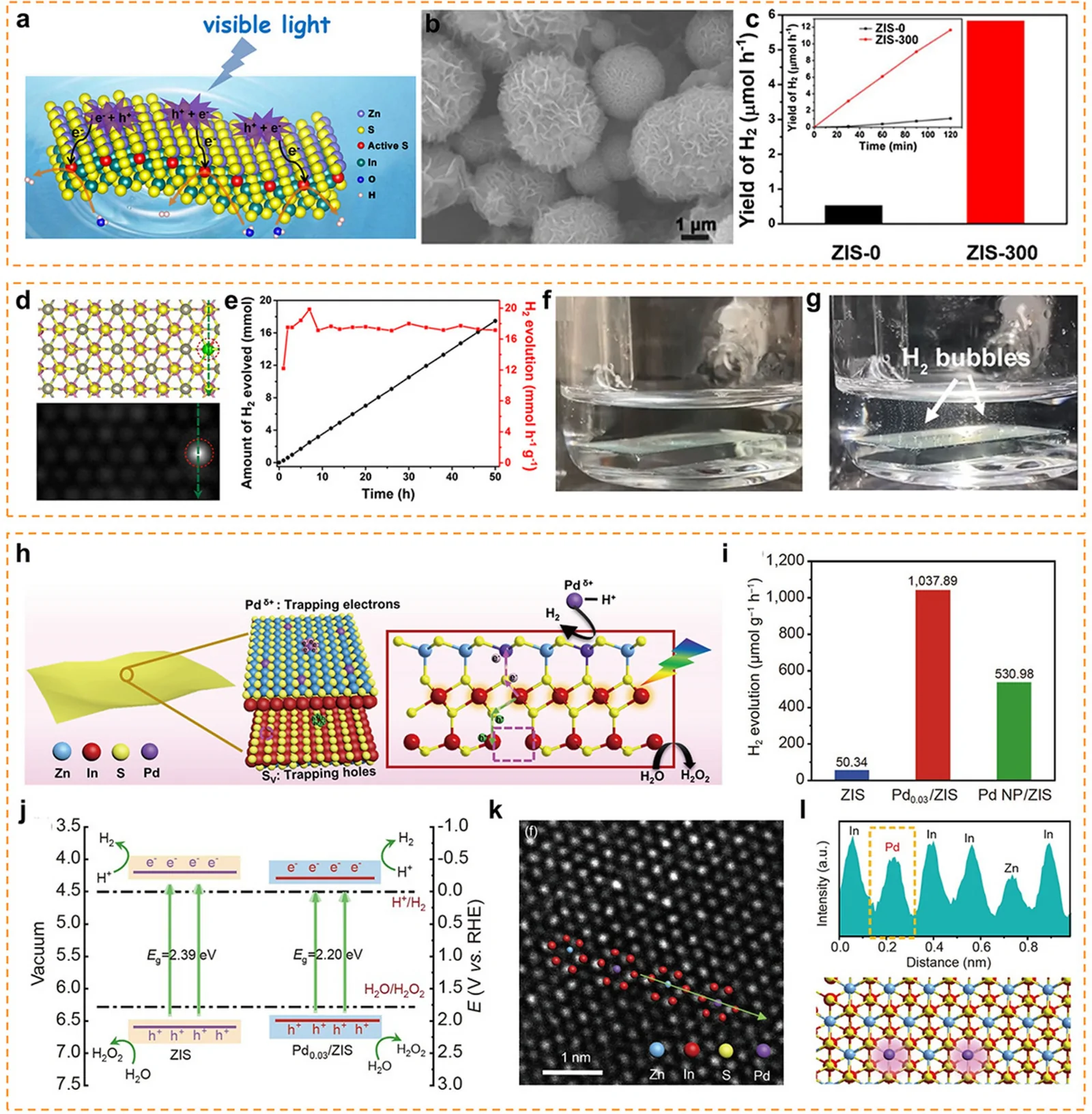
ZnIn_2_S_4_ sulfide supports and single-atom motifs for hydrogen evolution. **a–c** Ultrathin ZnIn_2_S_4_ nanosheets with enhanced (110) facet exposure and charge separation for cocatalyst-free PHE [127]. Copyright 2020, Elsevier. **d**–**g** Protruding Pt-S_3_ single sites on ZnIn_2_S_4_ enabling high-rate visible-light H_2_ evolution and film-based bubble generation [25]. Copyright 2022, Springer Nature. **h**–**l** Pd single atoms cooperating with sulfur vacancies in ZnIn_2_S_4_ for sacrificial-agent-free pure water splitting to H_2_ and H_2_O_2_ [128]. Copyright 2023, Springer Nature

Building on CdS, where single atoms often mitigate photocorrosion while extracting electrons, ZnIn_2_S_4_ provides a layered sulfide support where surface sulfur ligands can bind isolated noble metals. For pristine ZnIn_2_S_4_ nanosheets, the bandgap is about 2.39 eV, so visible-light excitation is efficient. The key limitation is inefficient charge-carrier separation and utilization. Electrons and holes often recombine before reaching the surface, and sulfur sites are not equally active across facets [25, 54, 127, 128]. Recent ZnIn_2_S_4_ studies, therefore, follow a clear ladder: expose intrinsic active S sites by facet and thickness control, add atom-economical single-site electron sinks with defined coordination, and finally couple single atoms with vacancies to enable sacrificial-agent-free water redox. For example, Shi et al. built the host baseline by synthesizing ultrathin ZnIn_2_S_4_ nanosheets (2.6–5.0 nm) using a trisodium citrate-assisted hydrothermal route (Fig. 5a-c) [127]. DFT identified the sulfur atom on the (110) facet as the key PHE site. Thinning increased (110) exposure and shortened carrier travel distance. Femtosecond transient absorption showed a longer average electron decay lifetime of 359 ps. With TEOA and visible light (*λ* > 420 nm), cocatalyst-free PHE reached 1.94 mmol g^–1^h^–1^ and an AQE of 10.1% at 420 nm [127]. This work defines the facet-level origin of activity, but it still relies on sacrificial donors.

On this activated host, Shi et al. stabilized ultralow Pt single sites (0.26 wt%) by a photochemical reaction, forming a protruding Pt-S_3_ tetrahedral motif on the basal plane (Fig. 5d-g) [25]. Compared with defect-trapped Pt single sites, the protruding geometry improved electron extraction and local proton delivery. Under visible light (*λ* > 420 nm) with 10 vol% TEOA, the H_2_ yield rate increased by a factor of 2.2 and reached 17.5 mmol g^–1^ h^–1^ [25]. A drop-cast film produced visible H_2_ bubbles. In addition, Sun et al. advanced practicality by coupling Pd single atoms with sulfur vacancies in ZnIn_2_S_4_ nanosheets (Fig. 5h-l) [128]. AC-HAADF-STEM and intensity analysis indicated isolated Pd that replaces part of Zn sites, while the bandgap decreased to 2.20 eV [128]. In pure water without any sacrificial agent, Pd0.03/ZIS produced H_2_ and H_2_O_2_ in a stoichiometric ratio, with productivities of 1037.9–1021.4 μmol g^–1^ h^–1^, respectively [128]. This replaces sluggish O_2_ evolution with a two-electron H_2_O_2_ pathway, but it raises needs for H_2_O_2_ management and long-term stability.

As summarized in the updated Table 3, several non-noble-metal single-atom systems (e.g., CdS@Ni, ZnIn_2_S_4_@Ni1/UiO-66-NH_2_, and ZnIn_2_S_4_@Cu) achieve very high mass-normalized H_2_ evolution rates under visible-light irradiation. However, comparison based on AQY/AQE reveals a contrasting trend: The highest AQYs in sulfide-supported single-atom photocatalysts are predominantly reported for noble-metal systems, particularly Pt and Pd-based single atoms, indicating more efficient photon-to-hydrogen conversion. This contrast suggests that peak H_2_ evolution rates alone do not necessarily reflect effective light utilization. Precious-metal single atoms more reliably translate absorbed photons into productive interfacial charge transfer and proton reduction, leading to higher AQYs and reduced wavelength sensitivity. In this context, noble-metal single atoms function not only as active sites but also as mechanistically transparent electron sinks. These insights naturally motivate a shift toward oxide supports, particularly TiO_2_, where strong metal–oxygen coordination, well-defined electronic structures, and superior chemical stability enable deeper mechanistic interrogation of noble-metal single atoms in photocatalytic H_2_ evolution.

**Table 3:** Research status of single-atom photocatalysts supported on CdS and ZnIn_2_S_4_ for hydrogen production

Support	Single-Atom Site/Coordination	Catalyst amount (mg)	Sacrificial agent	Light Irradiation HER	H_2_ Evolution Performance (mmol g^−1^ h^−1^)	AQY / AQE (nm)	Year
CdS@PCOF	Pt single atom	5	17% LA, 24 mL	700 mW cm^−2^ *λ* > 420 nm	110	62.2% (420) 64.8% (450)	2025 [50]
CdS	Pt single atom	10	10% benzyl alcohol, 50 mL	OP was not quantified/ *λ* ≥ 420	5.4	30.5% (420)	2025 [129]
CdS	Ni single atom	5	0.35 M Na_2_S and 0.25 M Na_2_SO_3_, 100 mL	OP was not quantified/ *λ* > 420 nm	20.28	13.83% (380)	2024 [130]
CdS_x_-Twins	Pd single atom	50	Lactic acid, 10% or 0.25%	802 mW cm^–2^, *λ* ≥ 420 nm	154	90.2% (475)	2024 [131]
ZnIn_2_S_4_/WO_3_	Pt single atom	10	Simulated seawater + Na_2_S/Na_2_SO_3_, 10 mL	*λ* > 420 nm	3.71	4.25% (420)	2026 [132]
Nitrogen-doped ZnIn_2_S_4_	Ni single atom	20	10% TEOA, 150 mL	–	22.5	46.1% (365) 19.8% (420) 3.8% (450) 0.6% (500)	2025 [133]
ZnIn_2_S_4_@UiO-66-NH_2_	Ni single atom	10	20% TEOA, 50 mL	*λ* > 400 nm	37.54	31.6% (400) 13.9% (420) 7.8% (450) 0.7% (500)	2024 [56]
ZnIn_2_S_4_	Pt–S_3_	20	10% TEOA, 45 mL	Visible-light	17.5	31.6% (400)	2022 [25]
ZnIn_2_S_4_	Cu single atoms	10	10% TEOA, 50 mL	*λ* > 420 nm	41.10 ± 3.43	20.81% (420 ± 15)	2022 [134]

### Oxide Supports Including TiO_2_ for Photocatalytic H_2_ Evolution

TiO_2_ is the most widely used oxide support for noble-metal single-atom photocatalysts and serves as a benchmark platform, particularly for studies emphasizing photon utilization efficiency and mechanistic clarity rather than peak H_2_ evolution rates alone. Its chemical stability in aqueous media, resistance to photocorrosion, and well-established synthetic toolbox enable precise control over crystal facets, defect density, and film thickness, which directly regulate metal–support interactions, interfacial charge transfer, and site accessibility. Abundant surface oxygen atoms provide strong anchoring sites for Pt–O coordination, effectively suppressing aggregation at ultralow loadings. In photothermal-enhanced PHE, these features make TiO_2_ a stable platform for probing heat-assisted interfacial charge transfer and hydrogen-related surface processes. However, its wide bandgap and weak intrinsic visible-light photothermal response usually require defect engineering, black TiO_2_ formation, or heterostructure coupling to improve solar absorption and local heat generation. More importantly, TiO_2_ is not a passive scaffold. It can participate in hydrogen storage, electron trapping, and surface/subsurface proton-coupled electron transfer. As a result, performance in TiO_2_-supported single-atom systems is governed by the balance among site stability, accessibility, and available charge flux rather than by atomic dispersion alone, making TiO_2_ an ideal platform for dissecting structure–function relationships in noble-metal single-atom photocatalysis.

**Fig. 6:**
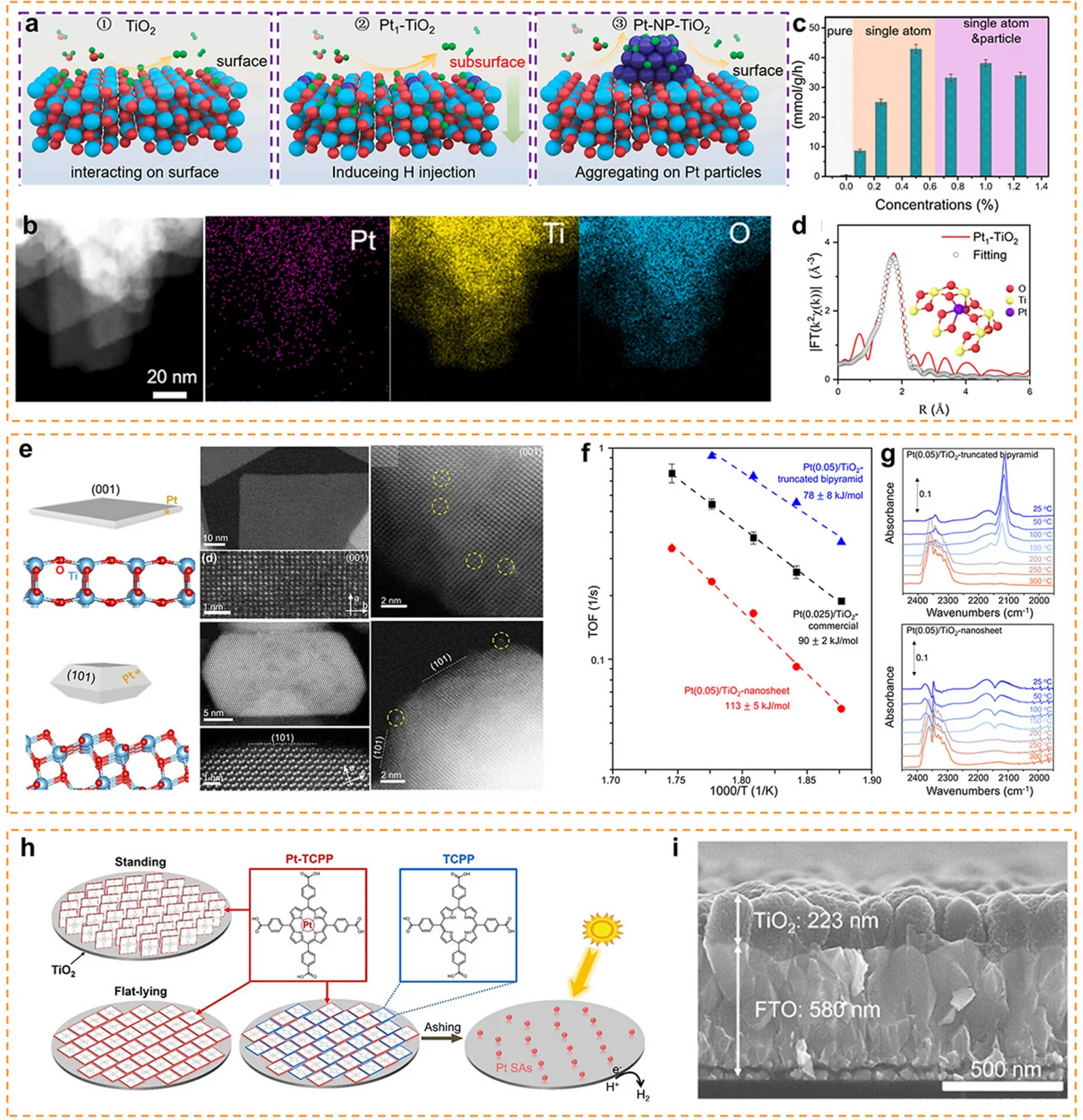
Pt single-atom engineering on TiO_2_ for photocatalytic H_2_ evolution. **a** Schematics comparing bare TiO_2_, Pt₁–TiO_2_ (subsurface H injection), and Pt nanoparticle–TiO_2_ (aggregation), with **b** STEM-EDS maps confirming a uniform Pt distribution on TiO_2_; **c** corresponding H_2_-evolution rate as a function of Pt concentration highlights a single-atom optimum, and **d** Pt K-edge EXAFS fitting further verifies the atomically dispersed Pt sites [135]. Copyright 2023, Elsevier. **e** Facet-dependent Pt anchoring on TiO_2_ truncated pyramids ((001)/(101)) with corresponding (HR)TEM images, **f** Arrhenius analysis of TOF for different TiO_2_ supports, and **g** operando IR spectra probing interfacial adsorbates [136]. Copyright 2024, Springer Nature. **h** Schematic illustration of porphyrin (Pt–TCPP)-mediated immobilization of Pt single atoms (SAs) on TiO_2_, and **i** cross-sectional SEM image of the TiO_2_/FTO film [19]. Copyright 2024, Wiley–VCH

Xue and coworkers demonstrated that Pt single atoms on anatase TiO_2_ nanosheets fundamentally modify hydrogen behavior during photocatalytic H_2_ evolution (Fig. 6a-d) [135]. Pt single atoms were introduced by wet chemical deposition using H_2_PtCl_6_, while Pt NPs prepared by NaBH_4_ reduction were used as a reference. In a water and triethanolamine system under Xe lamp irradiation, Pt single-atom TiO_2_ delivered a H_2_ evolution rate exceeding that of Pt nanoparticle TiO_2_ by more than four times (Fig. 6c) [135]. Extended X-ray absorption fine structure analysis confirmed isolated Pt oxygen coordination without Pt–Pt contributions (Fig. 6d) [135]. The key proposal was that Pt single atoms facilitate hydrogen injection into the TiO_2_ subsurface, forming a hydrogen-rich region that lowers the barrier for H_2_ release. This work established TiO_2_ as an active hydrogen reservoir, although direct operando evidence for subsurface hydrogen species is still lacking.

Building on the role of Pt depth, Zang et al. addressed how crystal facets control accessibility of Pt single atoms (Fig. 6e-g) [136]. Using strong electrostatic adsorption followed by calcination, low loading Pt was deposited on shape-controlled anatase TiO_2_. Pt single atoms on dominant 101 facets remained surface exposed, whereas those associated with 001 rich nanosheets migrated into subsurface positions after thermal treatment [136]. Carbon monoxide adsorption and oxidation kinetics revealed significantly lower reactivity for buried Pt sites. This study highlighted that thermodynamically stable Pt single atoms can be catalytically underutilized if they are not accessible to reactants under operating conditions. Finally, Qin et al. focused on maximizing utilization by matching Pt site density with photogenerated charge supply (Fig. 6h, i) [19]. Planar monolayers of Pt TCPP were assembled on TiO_2_ films and converted into isolated Pt sites after ligand removal. By co-adsorbing metal-free TCPP, the spacing between Pt atoms was precisely tuned. H_2_ evolution increased with Pt density and reached a maximum at an optimal spacing, beyond which activity saturated due to limited electron flux [19]. This work provided a practical design principle for TiO_2_ supported single atoms: Charge generation and lifetime should be optimized first, followed by adjustment of Pt density to match the available electrons. Although TiO_2_ itself has limited visible absorption, it serves as a model platform to interrogate how temperature reshapes interfacial barriers, defect-mediated electron trapping, and hydrogen spillover and injection pathways under coupled light heat fields. Therefore, TiO_2_-based SACs systems are particularly useful for validating mechanistic decoupling protocols discussed later in this review.

TiO_2_ is a highly reliable oxide support for single-atom photocatalysts with excellent stability and strong metal–oxygen interactions to stabilize noble-metal atoms and boost interfacial electron extraction for hydrogen evolution. Its wide bandgap makes most high-performance cases in Table 4 adopt near-UV excitation to produce adequate charge carriers. Performance improvement is mainly realized by facet/morphology regulation and coordination-controlled single-atom construction for better stability and reproducibility. TiO_2_ acts as a steady benchmark linking atomic site structure and photocatalytic activity, offering design rules for complex systems. Based on such TiO_2_ platforms, the next section discusses hybrid supports and heterostructures to break single-support limits and realize efficient charge separation for photocatalytic H_2_ evolution.

**Table 4:** Research status of single-atom photocatalysts supported on TiO_2_ for hydrogen production

Support	Single-Atom Site/Coordination	Catalyst amount (mg)	Sacrificial agent	Light Irradiation HER	H_2_ Evolution Performance (mmol g^−1^ h^−1^)	AQY / AQE (nm)	Year
TiO_2_	Zn single atoms	10	33% methanol, 30 mL	*λ* > 400 nm	9.21	6.82% (400)	2026 [137]
TiO_2_	Pt single atoms	20	20% methanol, 50 mL	120 mW cm^−2^/ LED 365 nm	102.8	84% (365 ± 5)	2025 [138]
TiO_2_	Pd single atom	10	25% methanol, 120 mL	–	24.6	23.4% (365)	2023 [139]
TiO_2_	Pd and Cu dual atom	10	66.7% methanol, 120 mL	–	94.35	83.81% (365)	2023 [140]
TiO_2_	Pd single atoms	10	25% methanol, 120 mL	200 mW cm^−2^	53.64	40.16% (365) 5.97% (385) 0.98% (420)	2022 [141]
M-TiO_2_ (derived from M-MiL-125(Ti_v_)	Cu single atoms	20	66% methanol, 120 mL	500 W m^−2^/ AM1.5	101.7	56% (365)	2022 [26]
cake-like mesoporous TiO_2_	Cu single atoms	10	20% methanol, 90 mL	–	17.77	20.15% (365)	2022 [142]

### Hybrid Supports and Heterostructure Scaffolds

**Fig. 7:**
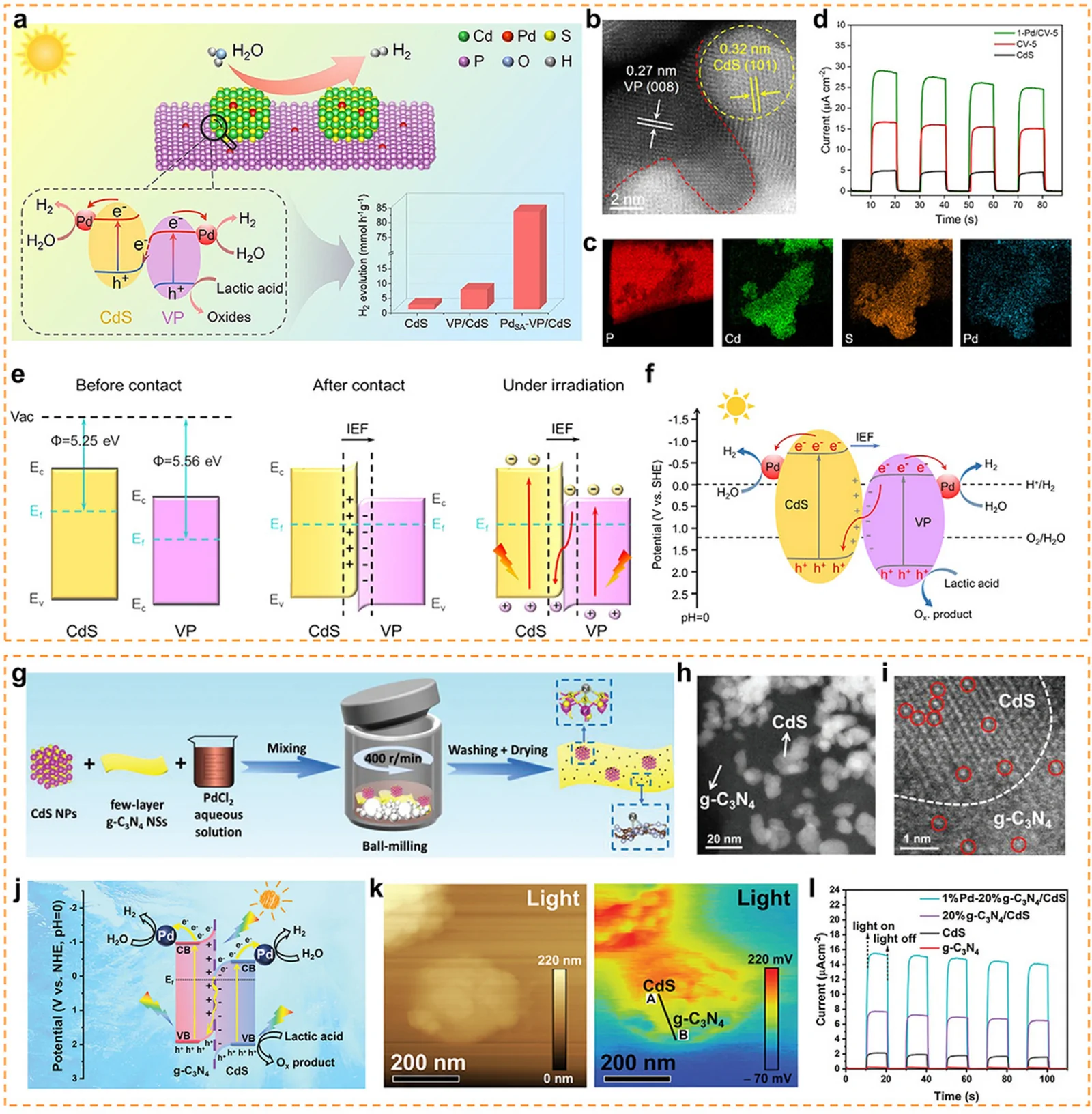
Pd single-atom/CdS–VP and Pd/g-C_3_N_4_–CdS heterostructures for photocatalytic H_2_ evolution. **a** Concept and activity comparison. **b** HRTEM identifies CdS(101) and VP(008).** c** Elemental maps verify Pd distribution. **d** Transient photocurrent responses. **e**, **f** Band alignment, interfacial electric field (IEF) and charge-transfer pathways under light [55]. Copyright 2024, American Chemical Society. **g** Ball-milling synthesis of Pd–g-C_3_N_4_/CdS. **h**, **i** STEM/AC-HAADF confirm interfacial contact and Pd single atoms. **j** Proposed Z-scheme charge transfer.** k** AFM/ KPFM maps under illumination. **l** Photocurrent enhancement with Pd loading [143]. Copyright 2024, Wiley–VCH

Hybrid supports and heterostructure scaffolds are used to host noble-metal single atoms and regulate photocarrier migration in photocatalytic H_2_ evolution. Because a single support rarely provides strong light absorption, efficient charge separation, stable single-atom anchoring, and favorable transport simultaneously, hybrid scaffolds divide these functions across different components, while engineered interfaces guide charge flow toward atomic catalytic sites. In photothermal-enhanced PHE, this division of labor should further include heat management. Photothermal domains, charge-transport interfaces, and HER-active single-atom sites need to be spatially coupled so that local heat generation is matched with directional electron delivery and accessible reaction pathways. COF matrices offer strong, designable coordination. PtSA@S-TFPT embeds low-valence Pt single atoms in a sulfur-containing COF and reaches 12.1 wt% Pt with 11.4 mmol h^–1^ g^–1^ H_2_ under visible light [18]. S-scheme design principles and verification tools are summarized in recent review papers [144]. S-scheme junctions are widely used because they enhance separation while retaining strong redox carriers for H_2_ chemistry. For example, Li et al. assembled few-layer violet phosphorene, CdS NPs, and Pd single atoms by a one-step ball-milling route (Fig. 7a-f). The VP/CdS contact forms a p-n heterojunction. The optimal 1 wt% Pd and 5 wt% VP sample delivers 82.5 mmol h^–1^ g^–1^ H_2_ with an AQE of 25.7% at 420 nm [55]. Here, VP enhances light harvesting, while Pd single atoms act as concentrated reduction centers on a separated charge scaffold. They also clarify the interfacial kinetics (Fig. 7e, f). In situ irradiated XPS and electron paramagnetic resonance (EPR) support an S-scheme charge-transfer pathway across VP/CdS [55]. Ultrafast electron transport of 2.2 ps is reported, with Pd-S and Pd-P bonds acting as transfer channels [55]. This shows how interfacial bonding can couple carrier separation to single-atom electron extraction. Finally, Pd single atoms are grafted onto g-C_3_N_4_/CdS S-scheme heterostructures via a one-pot mechanochemical method (Fig. 7g-l). The S-scheme pathway is corroborated by in situ irradiated XPS, EPR, and KPFM [143]. HAADF-STEM and XAFS identify Pd-S_3_ and Pd-N_2_ moieties, and carrier migration is accelerated to 1.05 ps [143]. The best sample reaches 85.66 mmol g^–1^ h^–1^ H_2_, 51.3-fold above pristine CdS [143]. This sets the stage for broader scaffold design.

These examples show that, in SAC-based S-scheme heterostructures, band alignment is only the starting point. The key question is whether the high-energy electrons retained by the S-scheme interface can be directionally delivered to isolated metal sites and rapidly consumed for proton reduction. Therefore, improved HER activity should be supported not only by S-scheme evidence, but also by charge-transfer signatures linking interfacial band bending to SAC-centered reduction chemistry.

Building on these representative heterostructure examples, Table 5 summarizes integration strategies for single-atom–heterostructure systems in PHE, spanning 2D/2D sulfide junctions, 2D/3D MOF–sulfide hybrids, carbon nitride composites, and oxide-containing heterojunctions. Despite structural diversity, these systems share an interfacial design principle that spatially separates light harvesting, charge separation, and catalytic sites, while directing electrons toward isolated metal atoms via interfacial charge transfer. A clear trend emerges that single atoms function as truly atom-economical reduction centers only when interfaces not only generate separated charges but also precisely funnel electrons to isolated sites through well-defined coordination pathways, thereby suppressing back recombination. Accordingly, Table 5 includes systems achieving tens of mmol g^–1^ h^–1^ hydrogen evolution under visible light and AQYs exceeding 50% at 420 nm [50, 53], highlighting heterostructures as a versatile toolbox tailored to different rate-limiting steps.

**Table 5:** Research status of single-atom photocatalysts supported on heterostructure for hydrogen production

Support	Single-Atom Site/ Coordination	Catalyst amount (mg)	Sacrificial agent	Light Irradiation HER	H_2_ Evolution Performance (mmol g^−1^ h^−1^)	AQY / AQE (nm)	Year
ZnIn_2_S_4_/g-C_3_N_4_	Ag single atoms	20	20% TEOA, 60 mL	*λ* > 420 nm	3.86	54.92% (420)	2025 [53]
In_2_O_3_/Nb_2_O_5_-10	Pd–S_4_	25	1 M TEOA, 50 mL	100 mW cm^−2^ *λ* > 400 nm	80.15	28.9% (400)	2025 [52]
CdS@PCOF	Pt single atoms	5	20 mL water + 4 mL lactic acid	700 mW cm^–2^ *λ* > 420 nm	110	62.2% (420) 64.8% (450)	2025 [50]
violet phosphorene /CdS	Pd–S/P dual-coordinated Pd_1_ sites	3	20% lactic acid, 5 mL	100 mW cm^–2^ *λ* > 420 nm	82.5	25.7% (420)	2024 [55]
β-ketoenamine-linked covalent organic framework (Tp-Tta COF)	C_3_–Co–N / N–Co–C_3_	50	10% TEOA, 80 mL	320–780 nm	1.80	3.82% (365) 3.02% (420)	2024 [47]
TiO_2_/g-C_3_N_4_ (TCN) S-scheme heterojunction	Ni single atoms	50	10% TEOA, 150 mL	–	0.13	15% (420)	2023 [49]
CCN/poly(triazine imide)	Co single atoms	50	10% TEOA, 30 mL	*λ* > 420 nm	3.54	20.88% (425)	2021 [48]

Across these support families, the same apparent rate enhancement can originate from different bottlenecks: carrier extraction on carbon nitrides, corrosion suppression and vacancy chemistry on sulfides, facet-directed site placement on oxides, or interface-directed charge routing in hybrids. This is why the next section switches from “which support” to “which metal role,” so that activity trends are interpreted through a consistent reactor-antenna framework rather than by material labels alone.

## Recent Progress in Photothermal-Enhanced Photocatalytic Hydrogen Evolution on Precious-Metal Single-Atom Sites

**Fig. 8:**
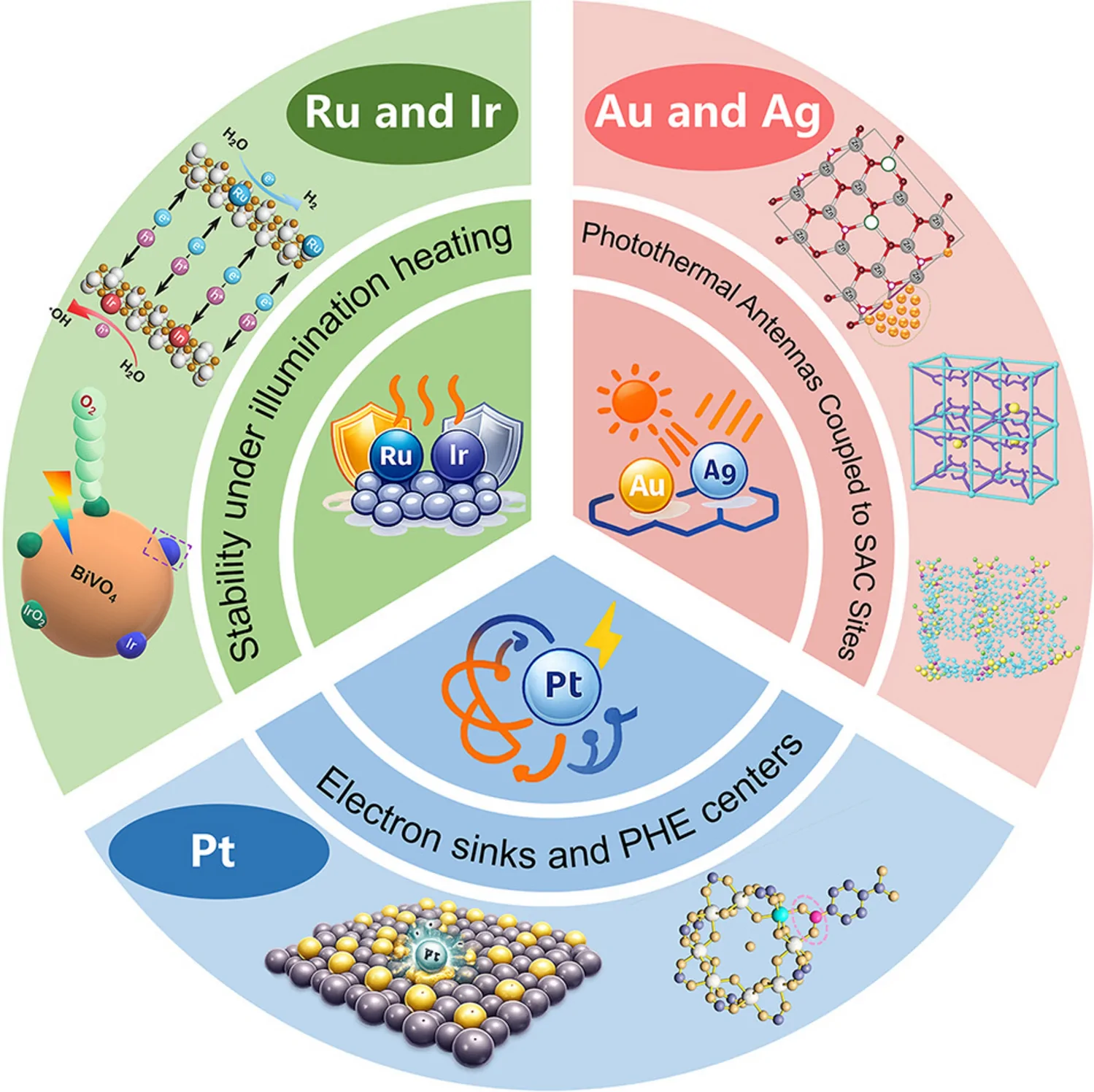
Functional role map of noble-metal motifs in photothermal-enhanced photocatalytic H_2_ evolution, highlighting Pt-centered electron-sink chemistry, Ru/Ir stability constraints under illumination-induced heating, and Au/Ag plasmonic antenna coupling

With these support-level design principles in place, the discussion now shifts from “where to host single atoms” to “what single atoms actually do under photothermal operation.” Building on the previous chapter’s discussion of how single-atom sites are anchored and how interfacial transport pathways are constructed, this chapter moves to a more immediate question: In photothermal-enhanced hydrogen evolution, noble-metal single atoms are not simply a matter of “switching the element.” In practice, they often enter the system’s energy and charge balance in different ways, and that ends up shaping both the apparent activity and the stability. As illustrated in Fig. 8, in some cases, the noble metal site mainly behaves as an electron extraction and accumulation center and directly promotes key surface steps in hydrogen evolution. In other cases, what stands out is how illumination-driven energy dissipation and local heating feedback on the coordination environment, induce valence drift, and open up deactivation pathways. And once plasmonic components are introduced, noble metals may additionally enhance light harvesting and couple thermal and charge processes at the interface, effectively amplifying the local driving forces in the reaction zone. Guided by these functional differences, this chapter will examine representative metal-based systems in turn, summarizing their structural characteristics, reaction behavior, and reproducible characterization signatures, and then comparing where different design routes converge, and where their boundaries become clear.

### Pt Single Atoms as Electron Sinks and PHE Centers

**Fig. 9:**
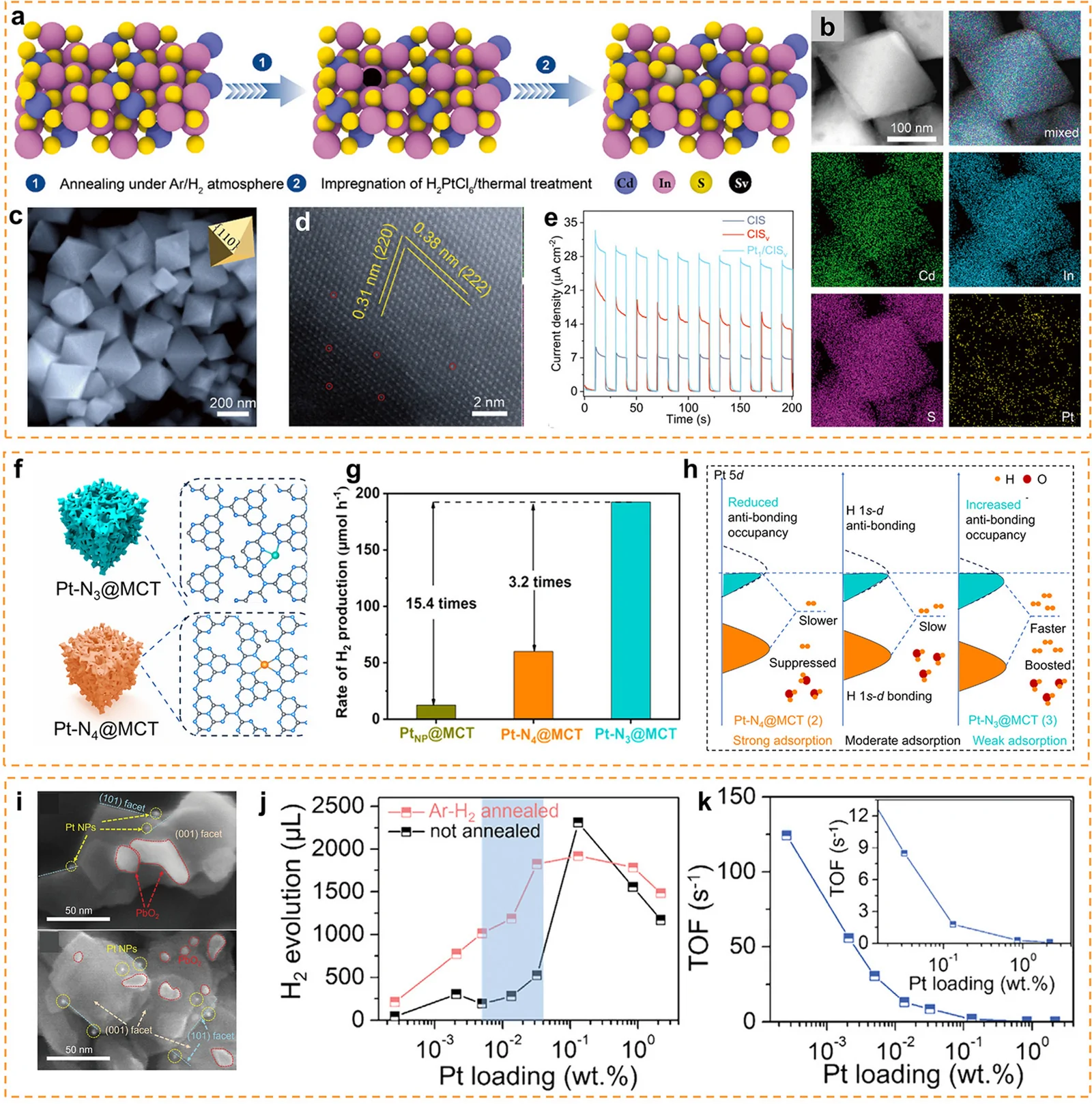
Coordination and loading effects in Pt single-atom catalysts. **a** Creating S vacancies then impregnating H_2_PtCl_6_ to anchor Pt on CIS. **b** STEM/EDS maps confirm Cd/In/S and dispersed Pt. **c**, **d** SEM and AC-HAADF show CIS crystals and isolated Pt atoms. **e** Transient photocurrent of CIS/CIS_v_/Pt–CIS_v_ [72]. Copyright 2025, Elsevier. **f** Schematics of Pt–N_3_@MCT and Pt–N_4_@MCT. **g** H_2_ evolution rates. **h** Coordination-dependent Pt–H adsorption and hydrogen evolution kinetics [145]. Copyright 2025, Elsevier. **i** Pt NPs *vs.* Pt SAs on facets. **j**, **k** H_2_ evolution and TOF versus Pt loading, revealing an optimum at low Pt content [146]. Copyright 2025, Wiley–VCH

Pt single atoms are among the most effective cocatalysts for photocatalytic H_2_ evolution because they integrate charge extraction and surface proton reduction in one atomic motif. At the semiconductor interface, an isolated Pt site can accept electrons and suppress recombination, a role favored by the relatively high work function of Pt and the resulting tendency to form electron-accepting interfacial contacts with many photoexcited semiconductors [147]. It also provides a defined coordination environment that tunes hydrogen intermediate binding and lowers the kinetic barrier of PHE. This catalytic function is closely related to the suitable Pt d-band position [148] and near-thermoneutral hydrogen adsorption free energy, which allow Pt–H interactions to remain neither too strong for H_2_ desorption nor too weak for H* formation [145]. Therefore, the benefit is maximized when Pt is strongly anchored, placed at the natural electron outlet of the host, and remains accessible to protons. Under these conditions, Pt most readily functions as both an electron sink and an HER reaction center. Classic atomic interface work on defective TiO_2_ established this concept, showing that an engineered interface can promote electron transfer from defect states to single Pt atoms [149]. More broadly, single-atom behavior can deviate from nanoparticle trends, so precise control of site structure is essential rather than assuming Pt is always optimal [150]. Recent progress can be organized into three complementary levers that are easy to compare: vacancy pinning on sulfides, coordination programming on carbon nitride, and facet selective placement on TiO_2_ [18, 25, 72, 145, 146, 149–153]. Chen and co-workers confined Pt single atoms on CdIn_2_S_4_ octahedrons stabilized by sulfur vacancies (Fig. 9a-e) [72]. They first annealed CdIn_2_S_4_ under Ar/H_2_ to create vacancy-rich surfaces, then introduced Pt via H_2_PtCl_6_ impregnation followed by thermal treatment to trap Pt as isolated atoms. Elemental mapping supports homogeneous Pt distribution, consistent with vacancy-assisted stabilization [72]. In performance tests for photocatalytic H_2_ evolution, the optimized Pt-based catalyst achieved 827.2 μmol h^–1^ g^–1^ under visible light, which is about 12 times higher than pristine CdIn_2_S_4_ [72]. Sulfur vacancies contribute in two ways. They pin Pt and suppress sintering, securing the single-atom motif. They also create trap states that funnel electrons to Pt, strengthening its role as an electron sink and accelerating PHE turnover (Fig. 9e). A remaining improvement is to directly track vacancy healing and Pt coordination drift under long irradiation, especially under photothermal conditions where atomic mobility can increase.

Liu and co-workers moved from defect anchoring to coordination programming by constructing asymmetric Pt–N_3_ and symmetric Pt–N_4_ motifs on a carbon nitride scaffold denoted MCT (Fig. 9f-h) [145]. They used supramolecular precursor assembly and pyrolysis to build MCT, then combined Pt precursor introduction with controlled thermal conversion to lock distinct Pt–N coordination numbers (Fig. 9f). The comparison is mechanistically sharp. Pt–N_3_ is described as an electron collector that promotes charge separation and transfer and increases occupied Pt d character, which weakens the Pt–H interaction and accelerates PHE turnover. Correspondingly, Pt–N_3_@MCT delivered 195 μmol h^–1^, about 3 times Pt–N_4_@MCT (65 μmol h^–1^), and reached an apparent quantum yield of 34.1% at 420 nm [145]. The broader impact is conceptual. “Pt single atoms” are not interchangeable. Coordination number is a design knob that links electron-sink strength with intrinsic surface kinetics. A practical next step is to couple operando spectroscopy with kinetic analysis to identify how coordination shifts the rate-limiting step, and to validate activity under more stringent conditions with reduced reliance on sacrificial donors (Fig. 9h). Qin and co-workers emphasized spatial placement by concentrating Pt single atoms on the electron-exit facet of anatase [146]. Pt single atoms were deposited on single-crystal anatase nanosheets exposing minor (101) and major (001) facets, then subjected to thermal treatments in air, Ar, and Ar/H_2_ (Fig. 9i-k) [146]. Ar/H_2_ annealing drives Pt species to accumulate exclusively on the minor (101) facets, which are associated with electron exit, thereby maximizing the probability that electrons reach Pt sinks before recombination. At an extremely low overall Pt loading of about 0.005 wt%, they obtained 11.7 mmol h^–1^ g^–1^ H_2_ production (Fig. 9j) and a TOF up to 253 H_2_ site^–1^ s^–1^ (Fig. 9k) [146]. This TOF was evaluated based on the reported Pt loading/site-density basis, with the corresponding Pt SA density given as 3.1 × 10^4^ atoms μm^–2^ for samples prepared from a 0.2 μM Pt precursor solution. Pt loading was analyzed by AAS, while HAADF-STEM, XPS, and CO-DRIFTS were used to support the facet-selective distribution and isolated-state assignment of Pt species. This work sets a benchmark for Pt utilization, shifting the design question from “how much Pt” to “where to place Pt.” Future studies should verify whether facet selectivity and single-atom dispersion persist under coupled light and heat, where Pt redistribution can confound mechanistic interpretation.

Recent studies demonstrate that Pt SACs serve as highly effective electron sinks and proton reduction centers in PHE, benefiting from maximized atomic utilization and strong metal–support electronic coupling. As summarized in Table 6, tailoring the coordination environment (e.g., Pt–N or Pt–O) and semiconductor support enables efficient charge separation, suppressed recombination, and enhanced H_2_ evolution rates across UV–visible excitation regimes. However, under intensified irradiation, photothermal effects inevitably arise, altering local reaction temperatures and interfacial kinetics. Such thermal contributions may further accelerate surface proton reduction, while simultaneously imposing stability challenges for isolated Pt sites. These considerations highlight the need to move beyond Pt-centric systems and examine alternative noble metal SACs with superior thermal robustness. In this context, Ru and Ir single atoms emerge as compelling candidates, whose stability and activity under illumination heating are discussed in the following section.

**Table 6:** Comparison of PHE performance of Pt single-atom catalysts on different supports

Support	Single-Atom Site/Coordination		Catalyst amount (mg)	Sacrificial agent	Light Irradiation HER	H_2_ Evolution Performance (mmol g^−1^ h^−1^)	AQY / AQE (nm)	Year
ZnIn_2_S_4_/WO_3_		Pt single atom	10	Simulated seawater + Na_2_S/Na_2_SO_3_, 10 mL	*λ* > 420 nm	3.71	4.25% (420)	2026 [132]
TiO_2-x_B_x_		B–Pt–O	5	66% methanol, 30 mL	5W/ 365 nm LED	627.6	98.4% (365)	2025 [28]
BTSO		Pt single atom	0.25	0.1 M ascorbic acid, 10 mL	100 mW cm^−2^ 320–780 nm	209	17.4% (360)	2025 [154]
COF@MOF		Pt–C + Pt–Pt	5	0.2 M ascorbic acid, 50 mL	*λ* > 420 nm	125.4	11.74% (420)	2025 [155]
N-rich g-C_3_N_4_ (N–CN_x_)		Pt_1_–N_4_	10	10%TEOA, 50 mL	128.3 mW cm^−2^/ IR cutoff	30.0	21.3% (400) 15.9% (365) 8.2% (450) 1.4% (550)	2025 [111]
O substitution doping N_2c_		Pt–N_4_ / Pt–N_2_ / Pt–N_3_O	2	10%TEOA, 20 mL	285 mW cm^−2^/ full-spectrum irradiation	66.4	26.44% (390) 24.3% (365) 1.7% (450)	2025 [112]
N-rich C_3_N_4_		Pt single atoms	6	10%TEOA, 30 mL	OP was not quantified/ UV cut-off	64.1	25.3% (420)	2025 [113]
g-C_3_N_4_		Pt single atoms (CD-stabilized)	10	20%TEOA, 100 mL	0.38 W cm^−2^ / AM 1.5	4.54	4.5% (420)	2024 [115]
FAPbBr_3-x_I_x_		Pt-X_3_	50	25 mM sodium phosphate buffer solution, 100 mL	AM 1.5G	6.826	STH = 4.50%	2022 [156]
ZnIn_2_S_4_		Pt–S_3_	20	10% TEOA, 45 mL	Visible-light	17.5	31.6% (400)	2022 [25]
MOF		Pt–O_4_	5	5% TEOA, 20 mL	*λ* > 380 nm	68.33	67.6% (420)	2018 [103]

### Ru and Ir Single Atoms as HER-Active and Thermally Robust Motifs

**Fig. 10:**
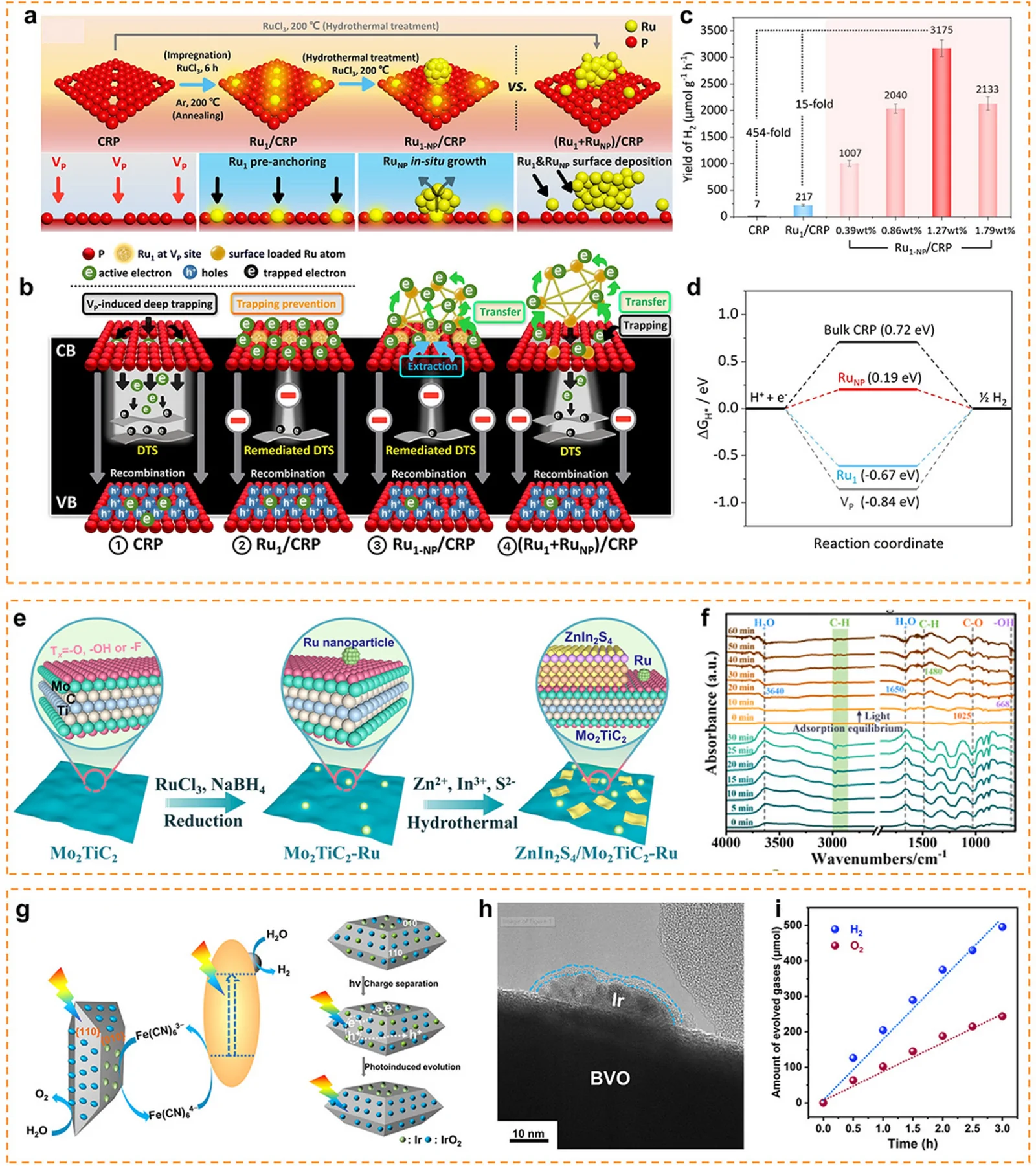
Ru and Ir single-atom cocatalysts enabling stable water splitting under illumination. **a**–**d** Stepwise construction of Ru_1_/CRP and Ru_1_ + Ru_*np*_/CRP, with enhanced H_2_ evolution and optimized ΔGH*, and mechanistic schemes for trapping prevention and electron extraction [24]. Copyright 2024, Wiley–VCH. **e**, **f** ZnIn_2_S_4_/Mo_2_TiC_2_–Ru synthesis and operando FTIR under light cycling, evidencing reversible electron storage and release [157]. Copyright 2024, Elsevier. **g**–**i** Ir on BiVO_4_: Z-scheme concept, TEM confirmation, and time-dependent H_2_/O_2_ evolution [158]. Copyright 2024, Elsevier

After Pt single atoms, Ru and Ir provide a valuable comparison because they also offer high atomic utilization, but exhibit different electronic structure and coordination responses under illumination-induced heating. When support-induced electronic structure modulation shifts the Ru d-band position and tunes the Ru–H binding strength, Ru single atoms can achieve a Pt-like $\Delta {G}_{{\mathrm{H}}^{*}}$, thereby serving as HER-active centers rather than merely stabilization motifs [24]. In contrast, Ir sites are generally more oxophilic and often function through high work function-related charge regulation, strong metal–support interactions, and redox-tolerant coordination, making them more suitable for maintaining charge separation and structural stability under light–heat stress [158]. This flexibility can strengthen anchoring, tune band bending, and improve charge separation, but it may also induce valence or coordination drift when local heating promotes surface diffusion or reconstruction. Therefore, when H* adsorption is optimized and electron delivery is efficient, Ru is more likely to serve as an HER-active site or electron-buffering site. By contrast, when oxidative tolerance and strong anchoring are required, Ir is more likely to act as a stable charge-handling motif. The studies in Fig. 10 illustrate three stabilization routes: defect repair, electron buffering, and strong metal–support interaction, which together sustain hydrogen output and retain atomic dispersion over time [24, 157, 158].

For example, Bian et al. designed a programmed Ru single atom plus nanoparticle bridge on crystalline red phosphorus (Fig. 10a, b) [24]. They first impregnated crystalline red phosphorus with RuCl_3_ and annealed at 200 ℃ to pre anchor Ru single atoms at phosphorus vacancy sites, giving Ru single atoms on red phosphorus. A second hydrothermal step grows Ru NPs from those anchored sites, forming Ru single atoms and Ru NPs on red phosphorus. The schematic highlights two roles of the pre-anchored Ru single atoms: suppressing vacancy-induced deep trapping and guiding uniform nanoparticle nucleation. The performance in Fig. 10c shows a strong increase in PHE, reaching 3175 μmol g^–1^ h^–1^ for the optimized sample. The free-energy diagram (Fig. 10d) explains why the hybrid works, red phosphorus defects trap charges, while Ru nanoparticle surfaces provide more suitable hydrogen adsorption. Impactfully, this work turns defects from liabilities into anchoring assets under irradiation.

Xi et al. used an electron parking cocatalyst to smooth illumination and heat fluctuations (Fig. 10e) [157]. They reduced RuCl_3_ with NaBH_4_ to deposit Ru on Mo_2_TiC_2_, then grew ZnIn_2_S_4_ on the Ru modified two-dimensional transition metal carbide or nitride (MXene) by a hydrothermal method to form ZnIn_2_S_4_/Mo_2_TiC_2_-Ru. The left schematic emphasizes the conductive MXene scaffold and the Ru modified surface as a capacitive electron reservoir. The key result is continuous hydrogen evolution even when light is switched off. The composite delivers 5.72 mmol h^–1^ g^–1^ under visible light and maintains about 1.67 mmol h^–1^ g^–1^ in the dark. The time resolved in situ infrared spectra in Fig. 10f support dynamic adsorption and reaction turnover during the storage release cycle. Conceptually, this approach complements Pt-based fast kinetics by improving reliability under real irradiance and heating transients [157]. Qi et al. demonstrated that strong metal support interaction can lock Ir motifs during irradiation, then used photoinduction to place dual cocatalysts on specific facets (Fig. 10g, h) [158]. They created Ir/BiVO_4_ with strong metal support interaction via high-temperature hydrogen reduction, then used in situ photoinduction to form Ir and IrO_2_ on different BiVO_4_ facets. The microscopy image shows an Ir overlayer on BiVO_4_, and the system delivers stoichiometric hydrogen and oxygen in a redox-driven Z scheme (Fig. 10i). The oxygen evolution activity is boosted by about 75 times, and the Z scheme reaches an apparent quantum efficiency of 16.9% at 420 nm. Although this is not a standalone sacrificial photo PHE test, it offers a strong stability message for illumination heating that Ir-based motifs remain highly dispersed when the interface is engineered to suppress migration and to guide redox evolution [158].

Table 7 summarizes representative Ru and Ir-based single-atom catalysts supported on oxides, sulfides, carbon nitrides, and molecular frameworks. Key parameters, including coordination environment, light irradiation conditions, hydrogen evolution rates, apparent quantum efficiencies, and publication years, are compiled to illustrate recent progress in non-Pt noble metal single-atom systems under photothermal-relevant excitation.

**Table 7:** Comparison of PHE performance of Ru and Ir single-atom catalysts on different supports

Support	Single-Atom Site/Coordination	Catalyst amount (mg)	Sacrificial agent	Light Irradiation HER	H_2_ Evolution Performance (mmol g^−1^ h^−1^)	AQY / AQE (nm)	Year
ZnIn_2_S_4_	Ru–S/Ru-Zn(In)	25	No Sacrificial	*λ* > 400 nm	0.58	4.36% (400)	2024 [159]
CNF (ZnO) nanocages	Ru–O	20	Aqueous Na_2_S and Na_2_SO_3_, 40 mL	*λ* ≥ 400 nm	5.8	3.8% (400)	2023 [160]
TiO_2_	Ru–O_6_/Ru–O_4_	5	1% benzyl alcohol, 50 mL	*λ* ≥ 300 nm	2.91	2.24% (365)	2023 [161]
Zn_0.5_Cd_0.5_S	Ru single atoms	20	17 mM furfuryl alcohol, 60 mL	*λ* ≥ 400 nm	0.87	\	2023 [162]
g-C_3_N_4_	Ru–N_4_	30	10% TEOA, 100 mL	*λ* ≥ 300 nm	4.05	3.8% (420)	2022 [163]
black TiO_2_ nanosheets	Ru–O	10	10% aqueous, 60 mL	332 mW cm^–2^	17.81	21.3% (365)	2022 [164]
Melem-cyameluric acid	Ir	20	No Sacrificial	100 mW cm^–2^ AM1.5 G	0.030	\	2025 [165]
BiVO_4_	Ir	50	25 mM sodium phosphate buffer solution, 100 mL	*λ* > 400 nm	4.0	16.9% (420 ± 10)	2024 [158]
PCN	Ir-Cl_3_/IrO_2_	50	10% TEOA, 100 mL	*λ* > 420 nm	1.56	15.7% (420)	2024 [166]
HNTM	Ir/Pt	50	8% TEOA, 50mL	*λ* > 400 nm	0.20	\	2018 [167]

In addition to Pt-based catalysts, Ru and Ir single-atom sites have been reported to deliver promising PHE activities under visible or simulated solar illumination, as summarized in Table 7. The collected studies indicate that appropriate metal–support coordination can stabilize isolated Ru and Ir atoms while enabling efficient charge utilization and proton reduction. Importantly, several systems maintain measurable activity under illumination heating, suggesting a degree of tolerance to thermally perturbed reaction environments. Rather than outperforming Pt, these systems expand the compositional and mechanistic landscape of photothermal-enhanced photocatalysis. Such observations motivate further discussion on how light-to-heat conversion can be deliberately harnessed at the atomic scale, leading naturally to Au- and Ag-based single-atom architectures functioning as photothermal antennas, as discussed in Sect. 3.3.

### Au and Ag as Photothermal Antennas Coupled to SAC sites

Au and Ag introduce an orthogonal design lever that is not available to most semiconductor hosts or isolated single-atom motifs [32, 33]. When Au or Ag is atomically dispersed and strongly coupled with the support, work function-mediated electron extraction and site-specific adsorption behavior can become dominant, allowing isolated Au or Ag single-atom sites to participate in charge-mediated reactant activation and proton reduction-related elementary steps [147, 171]. In their plasmonic nanoparticle or nanostructured forms, Au and Ag can further concentrate and spectrally extend photon harvesting, then redistribute the absorbed energy into near-field enhancement and local heating at the solid–liquid interface [170, 172]. In antenna–reactor architectures, the key question is not whether Au or Ag is intrinsically a better HER site than Pt, but whether plasmon-derived energy is delivered to an atomically defined reactor pocket quickly enough, and with a measurable temperature boundary condition, to raise interfacial electron flux and accelerate elementary steps without changing the reaction pathway. Au is generally more chemically robust in aqueous media, while Ag can exhibit stronger optical response but is more sensitive to oxidation and adsorbate chemistry [33]. Because these differences directly affect operando stability and attribution controls, Au and Ag systems should be evaluated with the same temperature-matched and wavelength-matched standards summarized in Sect. 4. Three design rules are repeatedly validated across Au and Ag antenna systems: (i) distance control, because energy delivery to a single-atom pocket is strongly separation-dependent; (ii) junction engineering, because Schottky barriers and band bending determine whether plasmon-assisted carriers actually reach the reactor site; and (iii) stability under realistic electrolytes, because surface oxidation or halide and sulfide adsorption can reshape the plasmon response and invalidate attribution if not monitored operando.

**Fig. 11:**
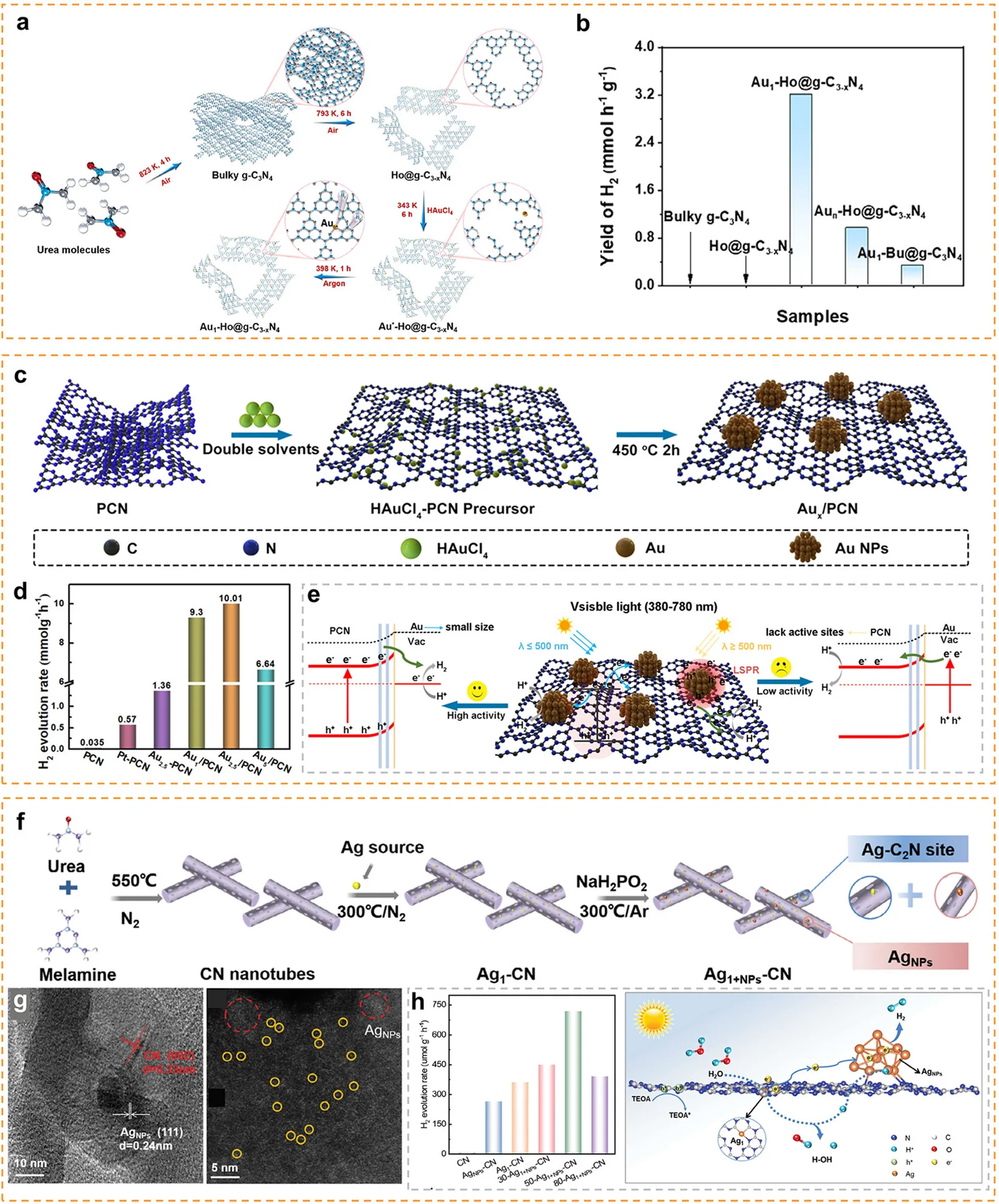
Au and Ag photothermal antennas coupled with single-atom like sites for photocatalytic PHE. **a** Schematic diagram for the synthesis process of bulky g-C_3_N_4_, Ho@g-C_3-x_N_4_, and Au_1_-Ho@g-C_3-x_N_4_ sample.** b** Photocatalytic H_2_ production performance [168]. Copyright 2025, Wiley–VCH. **c** Double solvent impregnation and pyrolysis for depositing Au on polymeric carbon nitride (Au on PCN). **d**–**e** H_2_ evolution rates and a proposed mechanism comparing small Au with vacancy assisted charge transfer versus large Au LSPR with reduced active site density [169]. Copyright 2023, Springer Nature. **f**–**h** Synthesis of Ag_1_–CN nanotubes with controlled Ag–C_2_N single sites and coexisting Ag NPs, with microscopy evidence and activity comparison [170]. Copyright 2025, Wiley–VCH

Xia et al. reported Au_1_–N_3_ single-atom sites anchored on carbon-defective holey g-C_3-x_N_4_ for efficient photocatalytic H₂ production (Fig. 11) [168]. The porous and defective carbon nitride framework stabilizes isolated Au atoms as Au_1_–N_3_ motifs rather than Au nanoparticles (Fig. 11a). These Au_1_–N_3_ sites introduce mid-band charge-trapping states, which promote electron transfer from g-g-C_3-x_N_4_ to Au single atoms and suppress electron–hole recombination. As a result, Au₁-Ho@g-C_3-x_N_4_ achieves a H_2_ evolution rate of 3.2 mmol g^–1^ h^–1^, corresponding to an approximately 333% increase compared with Au nanocluster-modified Ho@ g-C_3-x_N_4_ (Fig. 11b). This work highlights that Au single atoms can serve as efficient charge trapping and catalytic centers for photocatalytic hydrogen evolution.

Gao et al. provide the complementary “problem definition” on polymeric carbon nitride. Their double-solvent strategy first anchors HAuCl_4_ species on polymeric carbon nitride (PCN) and then converts them into dispersed Au NPs after thermal treatment (Fig. 11c) [169]. The bar chart highlights how Au loading controls activity, reaching an optimum H_2_ rate near 10.01 mmol h^–1^ g^–1^ for an intermediate Au content (Fig. 11d), while low loading gives only modest gains over PCN [169]. The mechanistic cartoon is the key message for this subsection. Under shorter wavelengths, small Au particles can collect electrons from PCN and support high activity, but under *λ* at least 500 nm the process becomes LSPR-dominated and activity drops because the surface lacks sufficient catalytic sites to consume plasmon-derived carriers efficiently (Fig. 11e) [169]. This directly motivates the next design step, namely, placing single-atom sites adjacent to Au antennas to serve as the missing reaction sinks. A useful upgrade for this line of work is a stricter wavelength-resolved action spectrum with matched photon flux and thermal controls to separate near-field from heating contributions.

Finally, Sun et al. show how Ag can follow a similar antenna–reactor logic when stability and dispersion are enforced by confinement (Fig. 11f) [170]. Carbon nitride nanotubes are first formed from urea and melamine at 550 °C under N_2_, then an Ag source is introduced at 300 °C under N_2_ to generate Ag_1_ sites, and a NaH_2_PO_2_ treatment at 300 °C under Ar partially reduces Ag to create a controlled mixture of Ag_1_ sites and Ag NPs [170]. The micrographs visualize this duality. High-resolution transmission electron microscopy (HRTEM) shows CN (002) fringes at 0.33 nm and Ag nanoparticle (111) fringes at 0.24 nm, while the annotated STEM image marks atomically dispersed Ag and larger Ag NPs (Fig. 11g). The activity plot indicates that moving from Ag_1_-CN to Ag_1+NPs_-CN steadily increases H_2_ evolution, with the best confined sample approaching the upper end of the displayed rate range (Fig. 11h) [170]. The mechanistic value is the explicit division of labor. Ag1 sites dominate H_2_O adsorption and dissociation, while Ag NPs facilitate H_2_ formation and desorption, so elementary steps are distributed across neighboring sites rather than forced onto one surface. Given Ag oxidation and halide sensitivity, post-reaction speciation using XPS and XAFS under relevant electrolyte matrices is necessary to validate long-term performance. Future improvements should address long-term photostability of Ag under realistic electrolytes and verify the proposed step assignment with operando spectroscopy.

Table 8 summarizes recent reports on Ag- and Au-based single-atom catalysts for PHE across a range of semiconductor supports. In these systems, coinage metal single atoms are frequently coupled with light-absorbing matrices such as g-C_3_N_4_, ZnO, and porous organic frameworks, where their electronic states and coordination environments are tailored to facilitate charge utilization. Notably, several studies combine isolated Ag or Au atoms with plasmonic NPs or extended conjugated supports, enabling enhanced light absorption over the visible spectrum and measurable hydrogen evolution activities under moderate irradiation. The reported AQY values and reaction rates indicate that, while coinage metal single-atom sites are generally less active for proton reduction than classical HER cocatalysts, they can nonetheless contribute meaningfully to overall activity by promoting hot-carrier generation, interfacial charge redistribution, or localized photothermal heating.

**Table 8:** Comparison of PHE performance of Au and Ag single-atom catalysts on different supports

Support	Single-Atom Site/Coordination	Catalyst amount (mg)	Sacrificial agent	Light Irradiation HER	H_2_ Evolution Performance (mmol g^−1^ h^−1^)		AQY / AQE	Year
g-C_3_N_4_	Ag single atoms + Ag nanoparticles	20	10% TEOA, 88 mL	–		≈ 0.70	1.6% (360)	2025[170]
g-C_3_N_4_	Ag–N + Ag nanoparticles	5	50% TEOA; 30 mL	*λ* = 400–700 nm		22.11	10.16% (420)	2025[172]
TiO_2_ / PF_3_T	Ag single atoms	5	Water:MeOH:TEA(1:1:1), 10 mL	*λ* ≥ 420 nm		3.26	–	2024[173]
PAF-164	Au single atoms	5	33% TEOA 5 mL	AM 1.5G		3.05	–	2024[171]
2D g-C_3_N_4_	Au single atoms	5	10% TEOA, 25 mL	–		7.2	9.24% (365)	2024[174]
ZnO	Au–O–Zn	10	0.35 M Na2_S_O_4_ and 0.25 M Na_2_SO_3_, 10 mL	UV–Vis		0.45	–	2024[175]
CN	Ag single atoms	10	20% TEOA,100 mL	*λ* > 400 nm		5.33	–	2022[176]
g-C_3_N_4_	Ag–N_4_ / Ag–N_3_	20	10% TEOA, 80 mL	*λ* ≥ 420 nm		2.0–2.2	6–7% (420)	2020[177]

Table 9 highlights the distinct functional roles of noble metals in photothermal-enhanced PHE. Pt, Ru, and Ir mainly act as catalytic or charge-handling centers, where their coordination environments determine electron extraction, H* adsorption/desorption, and stability under illumination. In contrast, Au and Ag more commonly serve as plasmonic antennas or photothermal components, enhancing light harvesting, local heating, and possible hot-carrier processes. Therefore, Pt/Ru/Ir-based systems should be evaluated mainly by atomic-site stability and charge-transfer kinetics, whereas Au/Ag-based systems require stricter thermal controls, wavelength-dependent tests, and local thermometry to distinguish productive plasmonic effects from simple heating artifacts.

**Table 9:** Role differentiation of noble metals in photothermal-enhanced PHE

Noble metal			Main role	Typical motifs	Dominant contribution	Key validation
Pt		HER catalytic center / electron sink		Pt–N_x_, Pt–O_x_, Pt–S_x_, facet-confined Pt_1_	Fast electron extraction, optimized H* adsorption/desorption, lower HER barrier	Atomic dispersion, Pt valence/coordination, charge-transfer kinetics
Ru	Catalytic center / electron-buffering site			Ru–N_x_, Ru–O_x_, Ru–S_x_, Ru–P, Ru_1_–Ru_NP_ hybrids	Defect regulation, electron storage/release, improved H adsorption	Ru coordination stability, light-on/off kinetics, long-term restructuring
Ir	Stable charge-handling / oxidative-tolerant site			Ir–O_x_, IrO_x_-like sites, Ir/Pt dual motifs	Charge separation, oxidative stability, stable water-splitting interfaces	Ir valence evolution, dispersion retention, H_2_/O_2_ stoichiometry
Au	Plasmonic antenna / photothermal heater			AuNPs, nanocages, Au–semiconductor junctions, Au_1_ sites	LSPR light harvesting, local heating, hot-carrier/near-field effects	Local thermometry, wavelength-dependent kinetics, hot carrier vs thermal controls
Ag	Plasmonic antenna / auxiliary adsorption site			Ag NPs, Ag–N_x_, Ag_1_ + Ag_NP_ motifs	Broad-spectrum absorption, charge redistribution, local heating, water activation	Ag oxidation/aggregation, action spectra, electrolyte stability

In practical photothermal-enhanced PHE, the most critical noble-metal function is not fixed, but is determined by which step limits the conversion of absorbed photons into H_2_. Electron-sink and HER-active functions are usually the first-order requirement, because photothermal heating cannot be efficiently translated into H_2_ production if photogenerated electrons recombine before reaching catalytic sites. Thus, in charge-limited carbon nitride, sulfide, and heterostructures, Pt-like sites are particularly important because their high work function, suitable d-band structure, and near-thermoneutral H adsorption promote electron accumulation and proton reduction. Once charge delivery is no longer the dominant bottleneck, the key issue shifts to whether atomic sites can survive the coupled light–heat field. Under intense irradiation, long-term operation, or thermally labile supports, Ru/Ir motifs become increasingly valuable because they combine charge regulation with stronger resistance to coordination drift, migration, and site deactivation. Au/Ag antenna units become decisive when photon harvesting or long-wavelength utilization limits the system. Even then, their plasmonic heating, near-field enhancement, or possible hot-carrier effects must be coupled to adjacent HER-active sites. Overall, charge extraction, light–heat stability, and antenna amplification should be matched to the specific bottleneck of each system, rather than maximized.

## Thermal and Photochemical Attribution in Photothermal-Enhanced PHE

Photothermal-enhanced PHE involves the concurrent conversion of absorbed photons into photogenerated carriers and local heat at or near the catalytic interface [41, 64]. The photochemical pathway relies on charge generation, separation, and electron transfer to HER-active sites, whereas the photothermal pathway originates from nonradiative relaxation, defect-mediated recombination, or plasmonic decay that converts part of the absorbed energy into heat [64]. This local heat can accelerate adsorption/desorption, proton-coupled electron transfer, H_2_ desorption, and interfacial mass transport [63]. However, these processes are difficult to attribute unambiguously because photon-driven charge processes and light-induced heating are initiated concurrently at the same interface, and both can produce similar apparent rate enhancements. When interpretation relies mainly on bulk temperature measurements, apparent kinetic trends, or limited control experiments, Arrhenius-type thermal acceleration can be readily conflated with pathways ascribed to photoexcited carriers [64].

To address this issue, it is first necessary to identify the key experimental parameters and reproducible characterization evidence for photothermal-enhanced PHE, so as to establish a comparable and verifiable basis for subsequent differentiation and mechanistic attribution of thermal, photochemical, and coupled light–heat contributions, as shown in Table 10.

**Table 10:** Key experimental parameters and reproducible characterization evidence for photothermal-enhanced PHE systems

Category	Key parameters/evidence to report	Corresponding tests or characterization methods	Main role
Reaction setup	Catalyst amount, solution volume, reactor geometry, stirring rate, gas atmosphere	Reactor photograph/schematic, gas-tightness test, GC calibration	Ensures reproducible H_2_ evolution testing
Reaction medium	Type and concentration of sacrificial agent, pH, solvent composition, ionic strength	pH meter, conductivity measurement, ion chromatography	Defines the chemical reaction environment
Optical input	Light source, irradiation wavelength range, optical power density, irradiation area, photon flux	Optical power meter, spectral irradiance meter, monochromatic light source/filter calibration	Defines photon supply and excitation conditions
Absorbed light	Catalyst absorptance, absorbed power, absorbed photon flux	UV–Vis DRS, integrating-sphere measurement, absorbed photon flux calculation	Enables fair comparison beyond incident light intensity
Thermal field	Bulk temperature, local/reaction-zone temperature, heating/cooling profile	Thermocouple, fiber-optic thermometer, IR thermography, Raman thermometry, luminescence thermometry	Distinguishes localized photothermal effects from bulk heating
Light–heat response	Light-on/off temperature change, thermal equilibration time, temperature stability during reaction	Light-on/off temperature curves, time-resolved temperature recording, thermal-imaging video	Evaluates photothermal dynamics
Catalytic output	H_2_ evolution rate, AQY/AQE, TOF, energy- or photon-normalized rate	Online GC, online MS, AQY/AQE measurement, TOF calculation	Enables quantitative comparison of catalytic performance
Stability test	Test duration, cycling number, activity retention, operating temperature	Long-term H_2_ evolution test, cycling test, continuous-flow or batch stability test	Evaluates operational durability
Photothermal attribution	Dark-heating control, temperature-matched light/dark reaction, wavelength dependence, light-intensity dependence, light-off test	Temperature-matched experiments, action spectrum, light-intensity-dependent kinetics, Arrhenius analysis, light-off benchmarking	Separates thermal, photochemical, and coupled light–heat effects
Atomic-site evidence	Atomic dispersion, coordination environment, valence state, electronic structure	HAADF-STEM, XANES/EXAFS, XPS, synchronous-illumination XPS, operando XAS	Provides *reproducible evidence* for SAC site structure and electronic state
Charge-dynamics evidence	Charge separation, electron extraction, recombination suppression, interfacial transfer rate	Transient photocurrent, EIS, TRPL, transient absorption, surface photovoltage, KPFM	Provides *reproducible evidence* for charge utilization promoted by single-atom sites
Reaction pathway evidence	Reaction intermediates, proton source, H_2_ formation process	Operando DRIFTS/Raman, isotope labeling, online GC/MS, EPR	Provides *reproducible evidence* for reaction pathway and product formation

Table 10 summarizes these parameters and evidence for photothermal-enhanced PHE systems. The key parameters include reaction setup, reaction medium, optical input, absorbed light, thermal field, catalytic output, and stability conditions, which define the reaction, photon, and heat boundary conditions required for reproducible H_2_ evolution tests. The characterization evidence includes photothermal attribution, atomic-site structure, charge dynamics, local heating, and reaction pathway verification, which help determine whether the observed enhancement originates from photothermal coupling, enhanced electron extraction, SAC site stabilization, or pathway changes. This table provides a practical reference for distinguishing thermal, photochemical, and coupled light–heat contributions, and offers guidance for rationally designing photothermal-enhanced PHE systems.

**Fig. 12:**
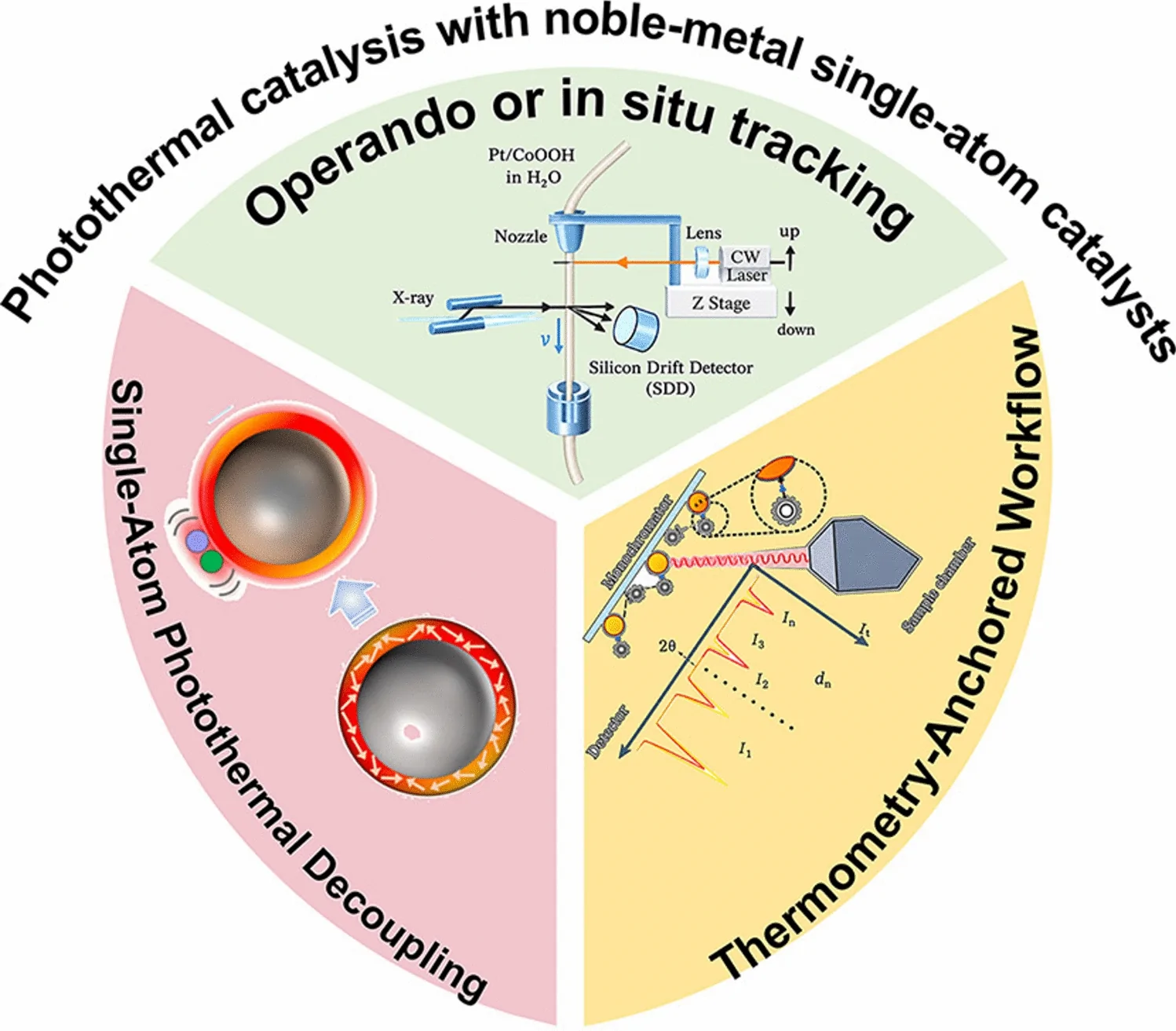
An attribution framework for photothermal-enhanced photocatalytic H_2_ evolution on noble-metal single-atom sites, integrating (i) structure-enabled decoupling in antenna and reactor architectures, (ii) thermometry-anchored diagnostics across length scales, and (iii) operando/in situ tracking that correlates site evolution, interfacial charge dynamics, and reaction kinetics under working conditions

Building on the parameter-and-evidence checklist in Table 10, Fig. 12 further organizes these requirements into an operando attribution workflow. A defensible attribution requires temperature, kinetic responses, and structural and electronic evolution to be evaluated under identical illumination and reaction conditions and aligned in time. Accordingly, Fig. 12 links three complementary components: structure-enabled single-atom photothermal decoupling, a thermometry-anchored diagnostic workflow, and operando/in situ tracking under illumination. Together, these components align temperature, structure, and reaction rate under the same conditions, providing a practical basis for separating predominantly thermal contributions from predominantly photon- and charge-driven contributions and enabling comparison across different material architectures.

### Conceptual Basis: Why Decoupling is Non-Trivial

**Fig. 13:**
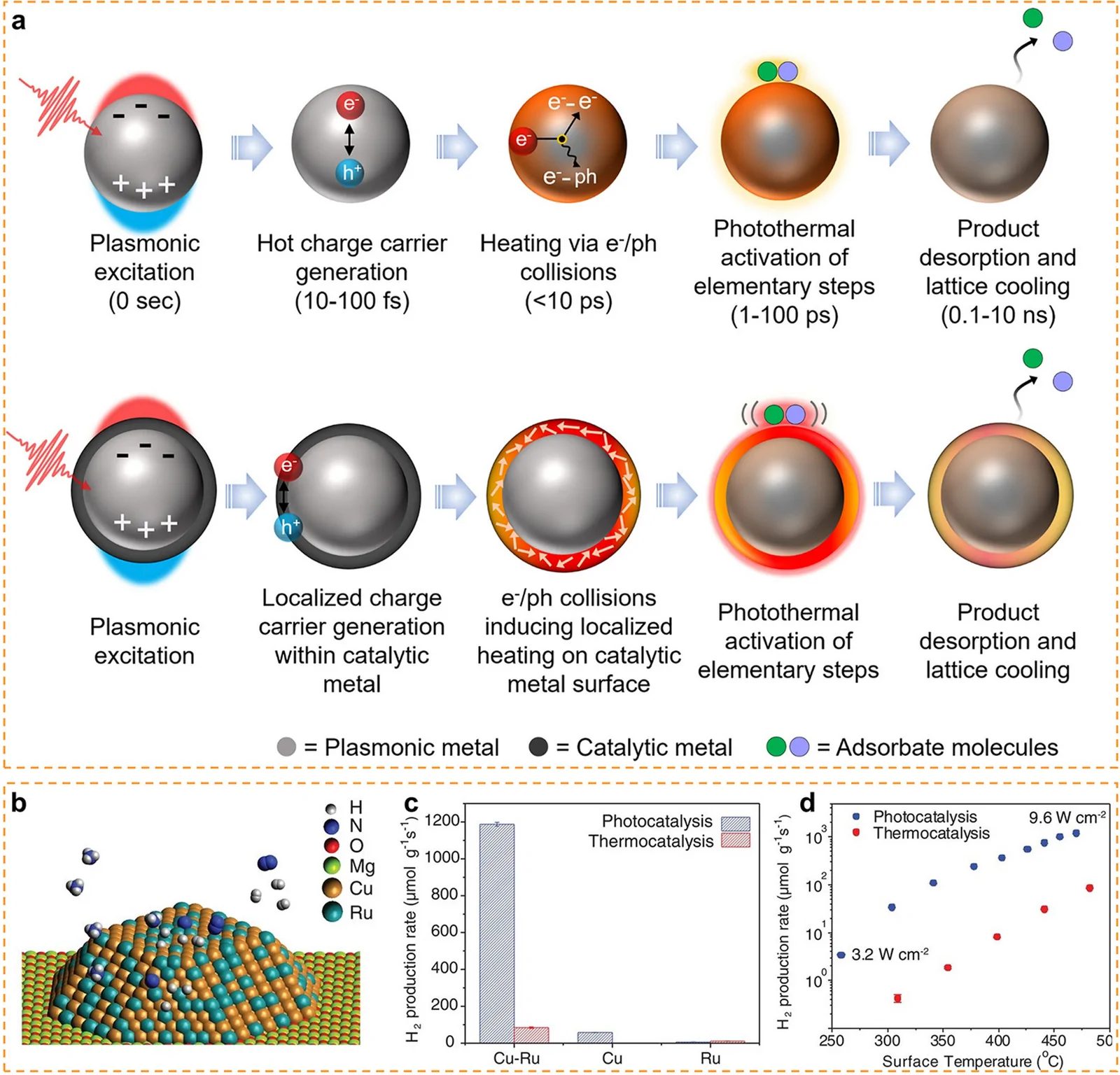
Plasmonic–photothermal pathways and temperature-driven H_2_ evolution. **a** Schematic time sequence from plasmon excitation to hot-carrier generation, electron–phonon heating, photothermal activation of elementary steps, and product desorption and cooling; comparison of charge generation in plasmonic versus catalytic metals [178]. Copyright 2025, Elsevier. **b, c** Atomic model of a Cu Ru catalyst and comparison of H_2_ production rates for Cu Ru, Cu, and Ru under photo and thermal conditions. **d** H_2_ production rate versus surface temperature, highlighting enhanced activity at higher local heating [37]. Copyright 2018, AAAS

Accurately delineating thermal and photochemical contributions in photothermal-enhanced photocatalysis (PHE) is essential for mechanism assignment and for establishing defensible structure–performance relationships (Fig. 13a) [41, 178]. Under illumination, photon absorption simultaneously generates non‑equilibrium charge carriers and dissipates energy as heat through ultrafast nonradiative relaxation, such that the measured rate enhancement is intrinsically a convolution of carrier‑mediated chemistry and temperature‑driven acceleration within the same catalytic microenvironment (Fig. 13b-d) [37, 41, 179]. This coupling is especially strong in antenna–reactor architectures that localize optical absorption and nanoscale heating while also promoting interfacial charge transfer, and in multimetal systems, where illumination can induce restructuring or composition‑dependent adsorption that further obscures causal attribution [178, 180, 181].

Conceptually, the observed reaction rate can be treated as the superposition of a photochemical term that depends on photon flux and carrier energetics, and a thermal term governed by the local temperature rise under illumination, with the important caveat that these terms are not strictly separable because temperature can feed back on carrier lifetimes, surface coverage, and interfacial kinetics [38, 178, 180]. A useful unifying viewpoint is that illumination can modify the effective activation landscape through two distinct levers: (i) photochemical activation, where hot carriers or excited-state charge transfer alters the reaction coordinate or driving force, and (ii) thermal activation, where a higher local temperature accelerates thermally activated steps via Arrhenius-type kinetics [41, 178, 179]. Classic analyses in plasmonic catalysis formalized these ideas by introducing light-dependent activation barriers and by extracting thermal rates from in situ thermal profiling, which together highlight why light-intensity and wavelength dependences alone are rarely definitive signatures [37].

Accordingly, rigorous decoupling requires explicit experimental designs that independently constrain the temperature field and the photochemical driving force [41, 178]. Emerging best practices emphasize temperature-matched dark controls, spatially relevant thermometry, and orthogonal perturbations such as geometric or layer-thickness variation and time-domain or functional modulation of illumination to separate fast carrier kinetics from slower thermal transients [38, 64]. These principles are particularly critical for single-atom catalysts, where the electronic structure of isolated noble-metal sites, interfacial electric fields, and possible hemilabile coordination dynamics can strongly amplify the coupling between charge flow and heat flow, making multi-probe cross-validation a prerequisite for credible mechanistic claims in photothermal-enhanced hydrogen evolution [180]. In practice, the goal is not to prove a non-thermal mechanism with a single signature, but to assemble a falsifiable evidence stack in which temperature, photon flux, and absorption are independently constrained.

### Distinguishing Photothermal Effects from Hot-Carrier-Driven Mechanisms

The distinction between photothermal effects and hot-carrier-driven mechanisms requires an evidence chain rather than a single diagnostic experiment. In plasmonic or strongly absorbing SAC-based PHE systems, illumination may simultaneously generate non-equilibrium carriers, local electromagnetic fields, and heat through nonradiative relaxation. Therefore, an increased H_2_ evolution rate under illumination cannot by itself identify the dominant enhancement channel. A rigorous assignment should combine reaction-zone thermometry, temperature-matched dark controls, wavelength-dependent action spectra, intensity-dependent kinetics, and time-resolved or operando spectroscopy. In this context, photothermal enhancement should be assigned when the rate follows local temperature and can be reproduced or bounded by temperature-matched controls, whereas hot-carrier contribution requires direct or indirect evidence of ultrafast carrier generation, injection, or charge-transfer dynamics beyond thermal acceleration.

Timescale separation provides an important criterion for distinguishing photothermal effects from hot-carrier-driven mechanisms. As summarized in Table 11, the key carrier- and heat-related processes in photothermal-enhanced PHE occur across distinct temporal windows, from femtosecond hot-carrier generation and interfacial injection to picosecond–nanosecond electron–phonon coupling, nanoscale heat diffusion, and millisecond-to-second bulk thermal equilibration. The corresponding experimental probes and attribution values are also summarized to guide mechanism assignment.

**Table 11:** Timescale separation of thermal and hot-carrier processes in photothermal-enhanced PHE

Process	Typical timescale	Experimental probes	Attribution value
Plasmon excitation/hot-carrier generation	fs	Ultrafast pump probe, fs-Transient absorption (fs-TA) [182, 183]	Supports hot-carrier generation
Hot-carrier thermalization versus interfacial injection	tens of fs	Ultrafast spectroscopy, fs-TA, time-resolved PL, two-temperature modeling [183, 184]	Distinguishes thermalization loss from nonthermal charge-transfer pathways
Carrier trapping, recombination, and SAC-mediated charge separation	ps–ns–μs	TA, TRPL, photocurrent decay, operando spectroscopy [185, 186]	Connects ultrafast charge dynamics with charge availability at SAC sites
Electron–phonon coupling and local lattice heating	ps–ns	Ultrafast spectroscopy, Raman thermometry, thermoreflectance [187, 188]	Bridges hot-carrier relaxation and photothermal heat generation
Heat diffusion around particles or plasmonic domains	ns–μs–ms	Raman thermometry, luminescence thermometry, thermal modeling [187, 189]	Supports local photothermal contribution when correlated with reaction rate Identifies bulk-heating artifacts and sets the boundary for thermal controls
Bulk solution heating and reactor thermal equilibration	ms–s or longer	Thermocouple, IR imaging, light-on/off thermal transients [41, 190]	Identifies bulk-heating artifacts and sets the boundary for thermal controls

Importantly, the detection of ultrafast charge transfer cannot directly confirm that hot carriers govern steady-state H_2_ production kinetics. Slow processes including proton reduction, charge recombination, mass transport, and bubble release frequently act as the overall rate-determining steps, so ultrafast spectroscopic evidence must be cross-validated with steady-state metrics such as AQY, TOF, apparent activation energy, and normalized H_2_ evolution rate.

Such mechanistic misattribution is widespread in plasmonic and photothermal photocatalysis research. For instance, improved hydrogen production under intense irradiation is commonly ascribed to localized hot-carrier or photothermal effects, while only bulk solution temperature is tracked. In such cases, the apparent performance gain may arise from macroscopic reactor heating or bulk thermal interference. Likewise, enhanced activity around Au/Ag LSPR absorption bands is often attributed to hot-carrier injection, yet LSPR excitation can simultaneously induce localized heating, near-field amplification, light scattering, and hot-carrier generation, making spectral correlation alone insufficient for verifying non-thermal reaction pathways. Therefore, transient spectroscopy should be integrated with thermometry, wavelength/intensity controls, and steady-state catalytic metrics to identify the dominant contribution to H_2_ evolution.

To reliably distinguish and identify the dominant enhancement mechanism, comprehensive validation is essential, combining temperature-matched control experiments, wavelength- and intensity-dependent kinetic analysis, time-resolved spectroscopic characterization, and key steady-state evaluation parameters.

### Thermometry-Anchored Decoupling Workflow and Kinetic Descriptors

**Fig. 14:**
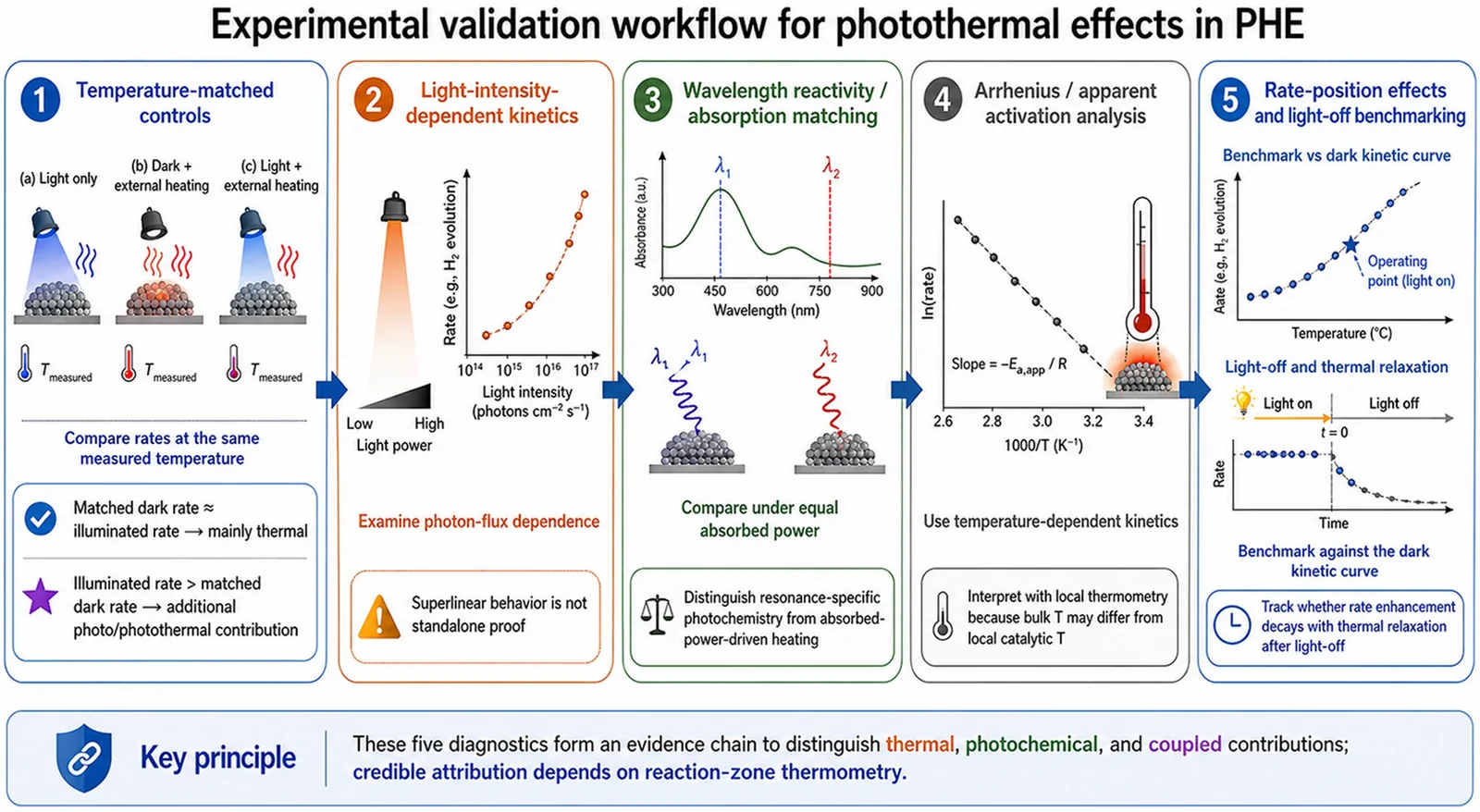
Quantitative separation of photochemical and thermal contributions during PHE. The scheme summarizes practical criteria and control strategies, including intensity kinetics, temperature-matched controls, wavelength and absorption matching, light-off benchmarking with rate-position effects, and Arrhenius-based apparent activation parameters, enabling identification of photochemical, thermal, and synergistic terms

Figure 14 summarizes the quantitative criteria and control strategies used to separate photochemical, thermal, and coupled contributions in PHE. The following discussion details the five complementary diagnostics, namely, intensity-dependent kinetics, temperature-matched controls, Arrhenius or apparent activation parameters, wavelength and absorption matching, and light-off benchmarking with rate-position effects.

*Step* 1: Temperature-matched control experiments. The most direct strategy is to compare (i) illumination without external heating, (ii) dark reaction with external heating to the same catalyst temperature reached under illumination, and (iii) illumination plus external heating [41, 179]. If the dark, temperature-matched rate equals the illuminated rate, the enhancement is predominantly thermal. If illumination yields a higher rate at the same measured temperature, a genuine photochemical contribution is implicated [41, 64, 179]. Reviews on methodology stress that the key difficulty is not the concept but the accuracy of temperature matching at the catalytic surface, since macroscopic thermocouples and thermal cameras may not reflect hotspot temperatures [41, 64].

*Step* 2: Light intensity-dependent kinetics. Reaction rates under illumination are often analyzed as a function of photon flux. A power-law dependence (rate ∝ intensity^*n*^, with *n* > 1) has been widely used as a diagnostic in plasmonic photocatalysis, where superlinear behavior has been discussed in the context of non-equilibrium carrier processes and local heating [191]. The early kinetic framework by Christopher, Xin, and Linic highlighted superlinear intensity dependence as a characteristic feature of plasmonic nanostructure photocatalysis, while later studies cautioned that superlinearity can also arise from photothermal heating when local temperature increases with absorbed power [41, 192]. Therefore, intensity dependence must be interpreted together with direct temperature tracking, absorption-normalized comparisons, and temperature-matched dark controls, rather than being treated as a standalone fingerprint of non-equilibrium carrier chemistry [41, 64].

*Step* 3: Wavelength-dependent reactivity and absorption matching. Photochemical pathways typically correlate with specific optical transitions, such as semiconductor bandgap excitation or plasmon resonance-driven carrier generation, whereas photothermal heating correlates more directly with absorbed power regardless of photon energy [37, 191]. Systematic wavelength scans under equal absorbed power, combined with temperature tracking, can separate absorption-driven heating from resonance selective photochemistry [37, 64, 191]. A widely cited quantitative framework is the “light dependent activation barrier” concept developed in the context of plasmon-mediated catalysis, where wavelength and intensity were varied while measuring catalyst surface temperature to evaluate thermal versus electronic contributions [37]. Although that specific case is not PHE, it provides a transferable analysis template for photothermal-enhanced photocatalytic reactions [37, 41].

*Step* 4: Arrhenius analysis and apparent activation parameters. Extracting apparent activation energies (or activation enthalpies) from temperature-dependent kinetics provides an additional quantitative handle [41]. A reduction of the apparent barrier under illumination compared with dark conditions is often interpreted as evidence for a light-driven electronic contribution beyond pure heating [37, 41]. For example, Jain and co-workers showed that visible excitation can lower the activation enthalpy of an Au nanoparticle catalyzed electron transfer reaction, with the magnitude depending on wavelength, power, and scavenger conditions [193]. Such behavior is difficult to rationalize by a purely thermal picture, but it is also a reminder that apparent activation energy barrier in illuminated catalysis can reflect changes in charge supply, interfacial potentials, and surface coverages, so apparent activation energy barrier analysis should be cross-validated with temperature-matched controls [41, 179]. A lowered apparent activation barrier can also result from a shifted rate-determining step under heating or from coverage changes, so it should be cross-checked against intermediate-sensitive spectroscopy and temperature localization uncertainty.

This temperature dependence also has implications for theoretical modeling. Most DFT studies used to interpret PHE-SAC systems rely on static geometry optimization at 0 K or near-room-temperature conditions, followed by standard free-energy corrections such as zero-point energy and entropy terms. This approach is useful for comparing H* adsorption or intermediate-binding trends, but it cannot fully describe photothermal operation, where the local temperature near catalyst particles or support–metal interfaces may exceed the measured bulk temperature. Under such conditions, adsorption free energies, adsorbate entropy, proton-coupled electron-transfer barriers, surface solvation, and even metal–support coordination may become temperature-dependent. Some photothermal water-splitting studies have begun to include temperature-dependent Gibbs free-energy analysis of intermediates, and broader first-principles studies commonly use: Δ*G *= Δ*E *+ Δ*ZPE –* TΔ*S* to include zero-point energy and entropy corrections. However, systematic finite-temperature DFT, ab initio thermodynamics, or AIMD studies under experimentally measured photothermal boundary conditions remain limited. Future theoretical studies should, therefore, connect adsorption free-energy corrections, reaction barriers, solvation effects, and structural dynamics with local or reaction-zone temperatures, rather than relying only on static 0 K models or nominal bulk temperatures.

*Step* 5: Rate position effects and “light off” benchmarking. Recent work has also shown that photothermal rate enhancements can differ dramatically depending on where the catalyst operates on the dark kinetic curve, making it essential to report dark reference kinetics across the relevant temperature range, not only a single temperature-matched point [38, 194]. This “light off” approach is useful when the reaction has steep Arrhenius sensitivity and small temperature uncertainties translate into large rate changes [64, 194].

Representative plasmonic catalysis studies provide useful methodological benchmarks for separating thermal, localized photothermal, and non-thermal carrier contributions. Zhang et al. compared temperature- and light-intensity-dependent kinetics over Rh/oxide plasmonic catalysts to distinguish thermal and non-thermal contributions in CO_2_ methanation [179]. Zhou et al. combined wavelength-, intensity-, and catalyst-temperature-dependent analysis on Cu–Ru antenna–reactor structures to quantify hot carrier and thermal contributions in ammonia decomposition [37]. Kim et al. examined wavelength- and power-dependent activation energies in Au-nanoparticle-catalyzed electron-transfer reactions, showing that illumination can modify apparent activation behavior [193]. Seemala et al. used Ag plasmonic nanostructures for O_2_ dissociation and combined experimental and theoretical analysis to distinguish hot-electron and near-field pathways, highlighting that Ag-LSPR activity also requires mechanism-specific controls [195]. Baffou et al. proposed practical procedures to distinguish photothermal from hot-carrier processes in plasmonic systems and emphasized that distant or bulk thermometry can miss localized heating [189]. Tiburski et al. introduced light-off benchmarking in plasmon-mediated CO oxidation to evaluate whether rate enhancement follows thermal relaxation after illumination is removed [194]. These studies show that LSPR-correlated activity enhancement alone is insufficient for assigning hot-carrier or localized photothermal pathways; wavelength/intensity controls, temperature-matched experiments, and local or reaction-zone thermometry are needed to distinguish different contribution channels.

Collectively, the five diagnostics in Fig. 14 provide an operational workflow for decomposing rate enhancement into thermal, photochemical, and coupled contributions. To clarify how these methods should be selected and interpreted, Table 12 summarizes their key requirements, main outputs, principal limitations, applicable conditions, and representative references. These methods should be treated as complementary diagnostics rather than independent proof of a single mechanism.

**Table 12:** Key decoupling methods for photothermal-enhanced PHE

Decoupling method	Key requirement	Main output	Main limitation	Applicable condition
Temperature-matched controls [41, 179]	Matched dark/illuminated temperature	Thermal baseline, residual light effect	Bulk T may miss hotspots	Initial separation of macroscopic heating from light-induced enhancement
Light-intensity-dependent kinetics [191, 192]	Controlled photon flux, temperature tracking	Rate–intensity exponent	Superlinear response is not proof of hot carriers	Evaluating whether rate is governed by photon flux or absorbed power
Wavelength reactivity/absorption matching [37, 191]	Monochromatic light, matched absorbed power	Action spectrum, absorption-normalized rate	Spectral correlation cannot separate heating, near-field effects, and hot carriers	Systems with plasmonic components or bandgap-selective excitation
Arrhenius/apparent activation analysis [37, 193]	Dark/light temperature-dependent kinetics	${E}_{a.app}$, barrier change	Affected by local T, coverage, charge supply, and RDS shift	Assessing thermally activated surface steps and light-induced apparent barrier changes
Rate-position/light-off benchmarking [38, 194]	Dark kinetic curve, light-on/off transients	Thermal relaxation, residual enhancement	Requires time-resolved rate and temperature data	Testing whether enhancement follows thermal relaxation or persists after light-off
Spatially relevant thermometry [64, 189]	Local/reaction-zone temperature measurement	Local T, gradients, hotspots	Difficult in liquid/slurry systems	Verifying localized photothermal heating and correcting bulk-temperature bias

In slurry reactors, temperature-matched dark controls should be interpreted with caution. External heating can match the measured bulk solution temperature, but it cannot fully reproduce local thermal gradients around illuminated catalyst particles, plasmonic domains, or support–metal interfaces. Therefore, a dark reaction at the same bulk temperature provides a macroscopic thermal baseline rather than a true replica of the illuminated photothermal state. In practice, reactor geometry, catalyst concentration, stirring rate, irradiation area, heating protocol, and probe position should be kept consistent, while bulk temperature matching should be combined with spatially relevant thermometry, absorbed-power normalization, wavelength- and intensity-dependent kinetics, and light-off benchmarking.

This comparison further highlights that the reliability of most decoupling methods depends on how accurately the reaction-zone temperature is constrained. In photothermal systems, the key uncertainty is often not the reactor or bulk temperature, but the local temperature at the catalytic locus, which may deviate because of optical absorption gradients, liquid heat dissipation, and nanoscale hotspots. This issue is especially important for SACs, where the active site is a specific coordination pocket rather than an average particle surface.

Accurate thermometry is, therefore, a prerequisite for assigning photothermal contributions in PHE. The catalytically relevant temperature is not a single scalar value: The bulk solution, reactor wall, illuminated particle, plasmonic domain, and SAC reaction site may experience different temperatures and relaxation times. As summarized in Table 13, thermometry techniques differ in the temperature they actually probe, their applicability in moving slurries, and their ability to distinguish local interfacial heating from macroscopic bulk heating.

**Table 13:** Advantages and limitations of representative thermometry techniques in photothermal-enhanced PHE

Thermometry technique	Temperature mainly probed	Typical spatial resolution	Typical temporal resolution	Main advantages	Major limitations in liquid-phase PHE	References
Thermocouple/fiber-optic thermometer	Bulk solution or probe-tip temperature	mm–cm	ms–s	Simple; low-cost; reactor-compatible	No hotspot resolution; probe-position dependent	[41, 64]
Infrared thermal imaging/IR thermography	Reactor wall, liquid surface, or apparent surface temperature	tens of μm–mm	ms–s	Non-contact; wide-field; reactor-scale mapping	Emissivity-dependent; window/water interference	[64, 190]
Raman thermometry	Local lattice or molecular temperature	sub-μm–μm	ms–s	Local; non-contact; structure-correlated	Weak slurry signal, laser heating; calibration-specific	[187, 196]
Luminescence-based thermometry/luminescent probes	Probe-nearby local temperature	nm–μm	μs–s	Remote; sensitive; micro/nanoscale mapping	Probe perturbation; photobleaching; matrix effects	[197]
Molecular/probe-reaction thermometry	Chemically experienced apparent temperature	molecular–ensemble scale	s–min	Chemically relevant; mechanism-sensitive	Indirect; reaction-dependent; not site-specific	[41, 198]
Operando multi-modal thermometry	Bulk, surface, local, and chemically inferred temperatures	nm/μm–reactor scale	μs–min	Multi-scale; cross-validated; most reliable	Complex; calibration-intensive; slurry-challenging	[41, 189]

Importantly, an increased H_2_ evolution rate under illumination should not be taken as direct evidence of localized photothermal kinetics. If only the bulk solution temperature is monitored, the observed enhancement may simply reflect a bulk-heating artifact, where the entire reaction medium is warmed by irradiation or reactor heat accumulation. Temperature-matched dark controls are, therefore, necessary to estimate the contribution of macroscopic thermal acceleration. However, such controls cannot fully reproduce the spatially localized thermal gradients generated around absorbing catalyst particles or plasmonic domains under illumination. Thus, the strongest evidence for local photothermal effects requires a combination of spatially relevant thermometry, temperature-matched controls, wavelength/intensity-dependent kinetics, and correlations between the reaction-zone temperature and H_2_ evolution rate.

While these thermometry-anchored diagnostics provide essential experimental evidence for distinguishing localized photothermal effects from bulk-heating artifacts, they mainly address the question of whether photothermal enhancement exists, rather than how much each pathway contributes to the overall reaction rate. In practical, photothermal catalytic systems, thermal activation, photon absorption, charge separation, interfacial electron transfer, and surface reaction kinetics are often strongly coupled, making quantitative attribution considerably more challenging. Therefore, beyond experimental decoupling strategies, a kinetic framework is also required to distinguish thermal acceleration from photocarrier-mediated contributions and to enable meaningful comparison across different catalytic systems.

Beyond phenomenological activity enhancement, a quantitative kinetic framework is needed to clarify how thermal activation and photocarrier-mediated processes jointly affect photothermal catalysis. It should be noted that no universal rate law can be applied to all photothermal catalytic systems, because the observed rate depends on local temperature, light absorption, carrier separation, interfacial charge transfer, and surface reaction steps. Therefore, the reaction rate should not be treated simply as a purely thermally activated process.

For photocatalytic hydrogen evolution, a simplified kinetic descriptor can be used to describe this coupling:

$${r}_{{\mathrm{H}}_{2}}\propto {\Phi}_{\mathrm{a}\mathrm{b}\mathrm{s}}{\eta}_{\mathrm{s}\mathrm{e}\mathrm{p}}{\eta}_{\mathrm{t}\mathrm{r}\mathrm{a}\mathrm{n}\mathrm{s}\mathrm{f}\mathrm{e}\mathrm{r}}{k}_{\mathrm{s}\mathrm{u}\mathrm{r}\mathrm{f}}({T}_{\mathrm{l}\mathrm{o}\mathrm{c}})$$

where ${\Phi}_{\mathrm{a}\mathrm{b}\mathrm{s}}$ is the absorbed photon flux, ${\eta}_{\mathrm{s}\mathrm{e}\mathrm{p}}$ represents charge-separation efficiency, ${\eta}_{\mathrm{t}\mathrm{r}\mathrm{a}\mathrm{n}\mathrm{s}\mathrm{f}\mathrm{e}\mathrm{r}}$ describes electron-transfer efficiency toward catalytic sites, and ${k}_{\mathrm{s}\mathrm{u}\mathrm{r}\mathrm{f}}({T}_{\mathrm{l}\mathrm{o}\mathrm{c}})$ denotes the local temperature-dependent surface reaction step. This descriptor is not intended as a universal kinetic equation, but rather as a mechanistic framework that separates photon utilization and carrier-transfer terms from the temperature-dependent surface reaction term. In this way, it helps clarify whether activity enhancement mainly arises from improved light harvesting and carrier utilization, thermal acceleration of surface kinetics, or their coupled contribution [199–202].

The use of Arrhenius-type analysis under photothermal conditions also requires caution. Conventional Arrhenius analysis assumes a spatially uniform and well-defined reaction temperature, whereas photothermal catalysts often involve localized heating, plasmonic hotspots, catalyst-bed temperature gradients, and wavelength-dependent heat generation [202, 203]. Therefore, the local catalytic temperature $\left({T}_{\mathrm{l}\mathrm{o}\mathrm{c}}\right)$ may differ substantially from the measured bulk temperature $\left({T}_{\mathrm{b}\mathrm{u}\mathrm{l}\mathrm{k}}\right)$. Under illumination, the apparent activation energy extracted from Arrhenius plots may include contributions from local heating, photocarrier generation, heat and mass transfer, and surface coverage changes, rather than only reflecting the intrinsic chemical activation barrier [202, 203]. Thus, reduced apparent activation energies should not be directly assigned to non-thermal hot-carrier effects without local temperature mapping or reactor-level heat-transfer analysis [202, 203].

For meaningful comparison among different photothermal catalytic systems, product formation rates alone are insufficient. Future studies should report optical power density, irradiation spectrum, catalyst absorptance, absorbed photon flux, measured temperature distribution, apparent activation energies under dark and illuminated conditions, apparent quantum yield/internal quantum yield when available, and reaction rates normalized by absorbed optical energy or illuminated catalyst area [204]. These descriptors can help decouple intrinsic catalytic kinetics from differences in light harvesting, heat generation, and reactor configuration, thereby enabling more reliable comparison across systems with different optical absorption and thermal characteristics.

### Operando Thermometry and Colocalized Chemo-Thermometry: Real Bottleneck

**Fig. 15:**
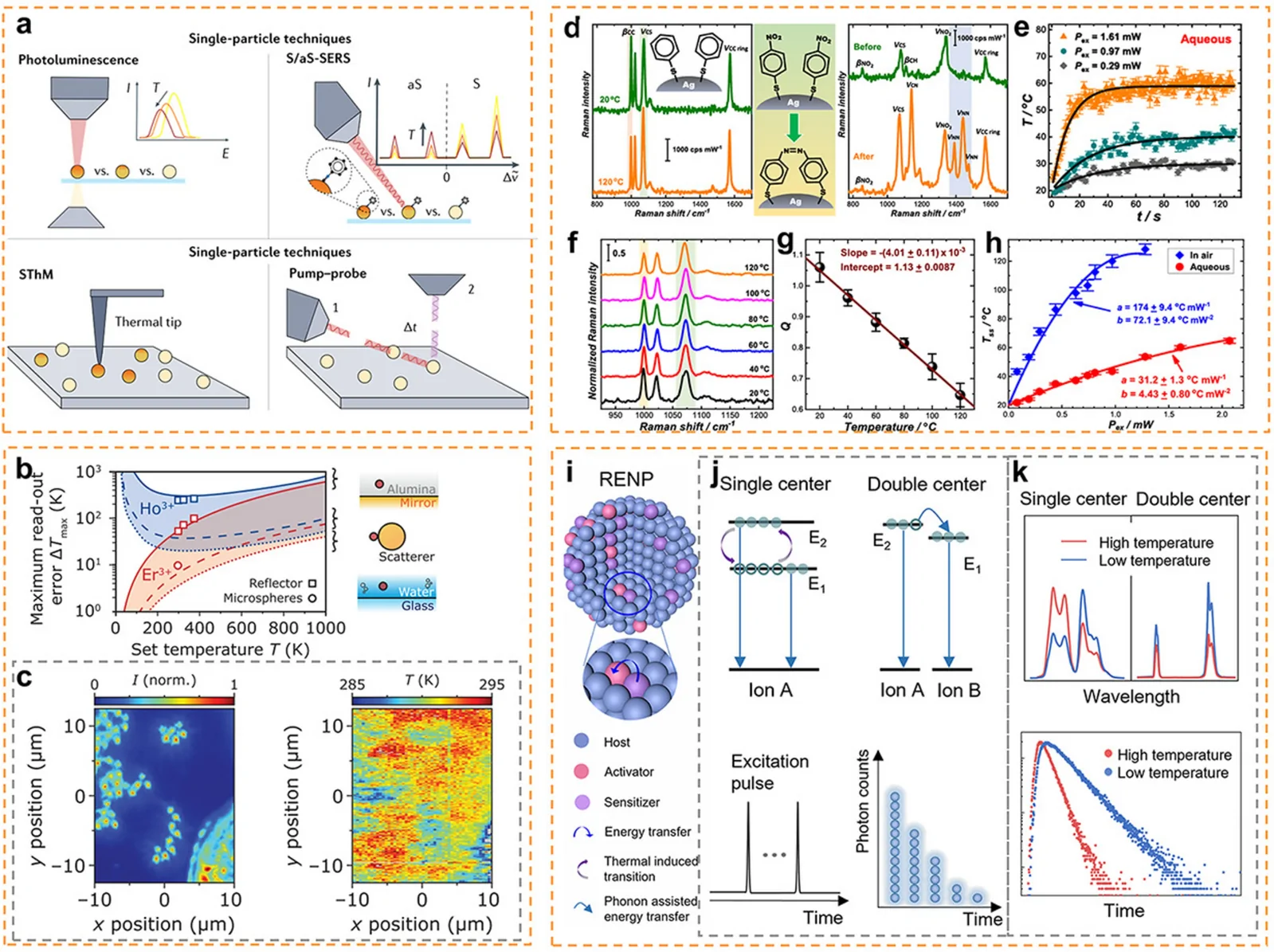
Single-particle thermometry and spectroscopy for nanoheating analysis. **a** PL, S/SERS, SThM, and pump–probe methods [205]. Copyright 2022, Springer Nature. **b**, **c** Maximum readout temperature and spatial maps of intensity and temperature [206]. Copyright 2023, American Chemical Society. **d**–**h** Raman thermometry workflow, calibration, and heating curves in air and aqueous environments [192]. Copyright 2023, American Chemical Society. **i** Typical structure of RENPs. **j** Basic working principles of PLIR. **k** Schematic representations of the PLIR [207]. Copyright 2024, American Chemical Society

If the local reaction-zone temperature is not constrained within a narrow bound, mechanistic assignment between photochemical and thermal pathways remains underdetermined, operando thermometry is, therefore, the real bottleneck. In practice, the dominant uncertainty is the temperature at the reactive site, not the reactor, holder, or bulk liquid [64]. Photothermal catalysts can sustain steep gradients across the fluid phase, particle surface, and nanoscale hotspots, so a single averaged readout can misassign the apparent driving force [41, 64]. This difficulty is amplified for single-atom catalysts, where the reaction zone is a specific coordination pocket at an interface and may be thermally and electronically decoupled from the particle-average state [180]. A rigorous operando thermometry strategy must explicitly state what temperature is measured, where it is measured, and under which optical boundary conditions [64]. Figure 15 highlights why “temperature” is method-dependent. Single-particle approaches (Fig. 15a) span temperature-dependent photoluminescence, (S) ERS anti-Stokes/Stokes nanothermometry, scanning thermal microscopy, and pump–probe transients [205]. Practical constraints, such as the readable temperature window, uncertainty, and spatial heterogeneity of hotspots, are emphasized by the maximum readout and error trends (Fig. 15b) and spatial maps (Fig. 15c), underscoring that particle-averaged values can obscure the true reactive locus [206].

Crucially, the most decisive evidence in photothermal catalysis comes from co-localized chemo-thermometry, i.e., measuring temperature and chemistry at the same locus. This is exemplified by surface-enhanced Raman scattering (SERS)/TERS workflows (Fig. 15d-f), where adsorbate fingerprints and temperature-sensitive spectral metrics are acquired simultaneously, enabling direct constraints on “thermal *vs.* nonthermal” claims at the operative hotspot [164]. Quantitative attribution further requires robust calibration (Fig. 15g) and explicit reporting of medium-dependent boundary conditions, since heat dissipation, and thus hotspot temperature, differs markedly between air and aqueous environments (Fig. 15e, h) [192].

In parallel, luminescence nanothermometry provides a complementary route that can be positioned closer to catalyst interfaces. Rare-earth-doped luminescent NPs (Fig. 15i) encode temperature through energy-transfer networks and thermally activated transitions; “single-center” versus “double-center” designs (Fig. 15j) enable ratio metric readouts, while time-resolved decays provide lifetime thermometry (Fig. 15k) [207]. These probes can mitigate artifacts from excitation and collection fluctuations, yet they still must specify probe placement relative to the true reaction pocket and ensure consistent optical boundary conditions and calibration. Otherwise, they may report temperatures that are not co located with the catalytic locus [64]. Overall, without operando, spatially resolved, and ideally chemo-co-localized, thermometry, photothermal attribution in slurry photocatalysis remains intrinsically ambiguous [64].

For plasmonic antenna architectures, the most informative methods are those that co-locate thermometry with chemistry [205]. Wang reviewed how SERS and TERS can quantify local temperatures in plasmonic hotspots via nanothermometry while simultaneously tracking adsorbate fingerprints, which directly constrains thermal versus non-thermal claims at the same active region [178, 208]. TERS primers and recent nanoscale thermal probing discussions also stress that hotspot thermometry must consider laser heating, junction geometry, and non-equilibrium effects that can perturb anti-Stokes and Stokes populations [209]. Chavez and co-workers further note that hybrid antenna–reactor systems add complexity because multiple metal sites, absorption changes, and restructuring can all masquerade as “nonthermal” enhancements unless temperature and chemistry are measured at the correct locus [178].

## Operando or In Situ Tracking of Photocatalytic HER

Section 4 establishes how to attribute rate enhancement by independently constraining temperature and photochemical driving forces. Building on this framework, Sect. 5 examines whether the proposed active motif is the true working site under coupled optical and thermal fields. For noble metal single atoms, the central issues are whether atomic dispersion persists during turnover, how first-shell coordination and oxidation state evolve under illumination and heating, and whether the electron-accepting state driving proton reduction forms only under operating conditions. These questions cannot be answered reliably by ex situ characterization, which often misses transient intermediates, dynamic restructuring, and interfacial phenomena. Operando and in situ methods are, therefore, essential because they track catalyst structure and surface chemistry in real time. The most convincing mechanistic studies combine element-specific probes such as XAS or synchronous-illumination XPS, intermediate-sensitive spectroscopy such as infrared spectroscopy/diffuse reflectance infrared Fourier transform spectroscopy (IR/DRIFTS) or Raman, and kinetics anchored by Sect. 4 attribution controls, so that mechanistic claims are supported by converging evidence rather than inference [210].

Accordingly, Table 14 summarizes the operando and in situ techniques most relevant to this challenge, clarifying what each method reveals and why it matters for SACs photothermal attribution. The table underscores that atomic stability, electronic-state evolution, reaction intermediates, and local temperature should be probed under working conditions. Only by integrating element-specific, intermediate-sensitive, and time-resolved measurements can rate enhancement be rigorously linked to the true active motif rather than to thermal artifacts or ex situ assumptions.

**Table 14:** Overview of operando and in situ characterization techniques for distinguishing photothermal and non-thermal effects in SACs

Technique	What it reveals	Why it matters for SACs + photothermal
Operando XAS	Dispersion, first-shell coordination, oxidation state under turnover [211–213]	Directly falsifies “single atoms remain isolated” and tracks valence drift during heating
Synchronous-illumination XPS	Surface oxidation state and chemical environment with light on [22, 107, 214–217]	Separates light-induced electronic changes from post-mortem artifacts
Operando DRIFTS or ATR-IR	Adsorbed intermediates, proton/hydride signatures, water and donor participation [18, 21, 215]	Links rate changes to pathway shifts rather than to temperature alone
Operando Raman	Lattice defects, adsorbates, local structure dynamics [213, 218]	Tracks defect healing, phase change, and adsorbate-driven reconstruction
EPR/Electron Spin Resonance (ESR)	Trapped electrons, radical intermediates [219, 220]	Distinguishes carrier trapping at supports versus at metal motifs
Transient absorption (TA)	Carrier generation, trapping, transfer kinetics [215, 221]	Quantifies whether carriers reach the reactor site before recombination under heating
Time-resolved photoluminescence (TRPL)	Recombination dynamics, quenching by SACs [21, 27, 28, 217, 222, 223]	Quick metric for electron-sink strength, must be paired with operando evidence
Nano-thermometry (Raman or luminescent probes)	Local temperature near active pockets or hotspots [192, 224]	Prevents misassignment caused by bulk temperature averaging
Isotope labeling (D_2_O, donor labeling)	Proton source and kinetic isotope effects [28, 108, 220]	Tests whether heating changes the rate-determining proton-coupled step
Online MS/GC with light-on/off protocols	True H_2_ formation dynamics, induction, deactivation [18, 27, 182]	Anchors spectroscopy to real-time kinetics and stability behavior

### Operando X-ray Tracking of Precious-Metal Single-Atom Sites in Photocatalytic Hydrogen Evolution

**Fig. 16:**
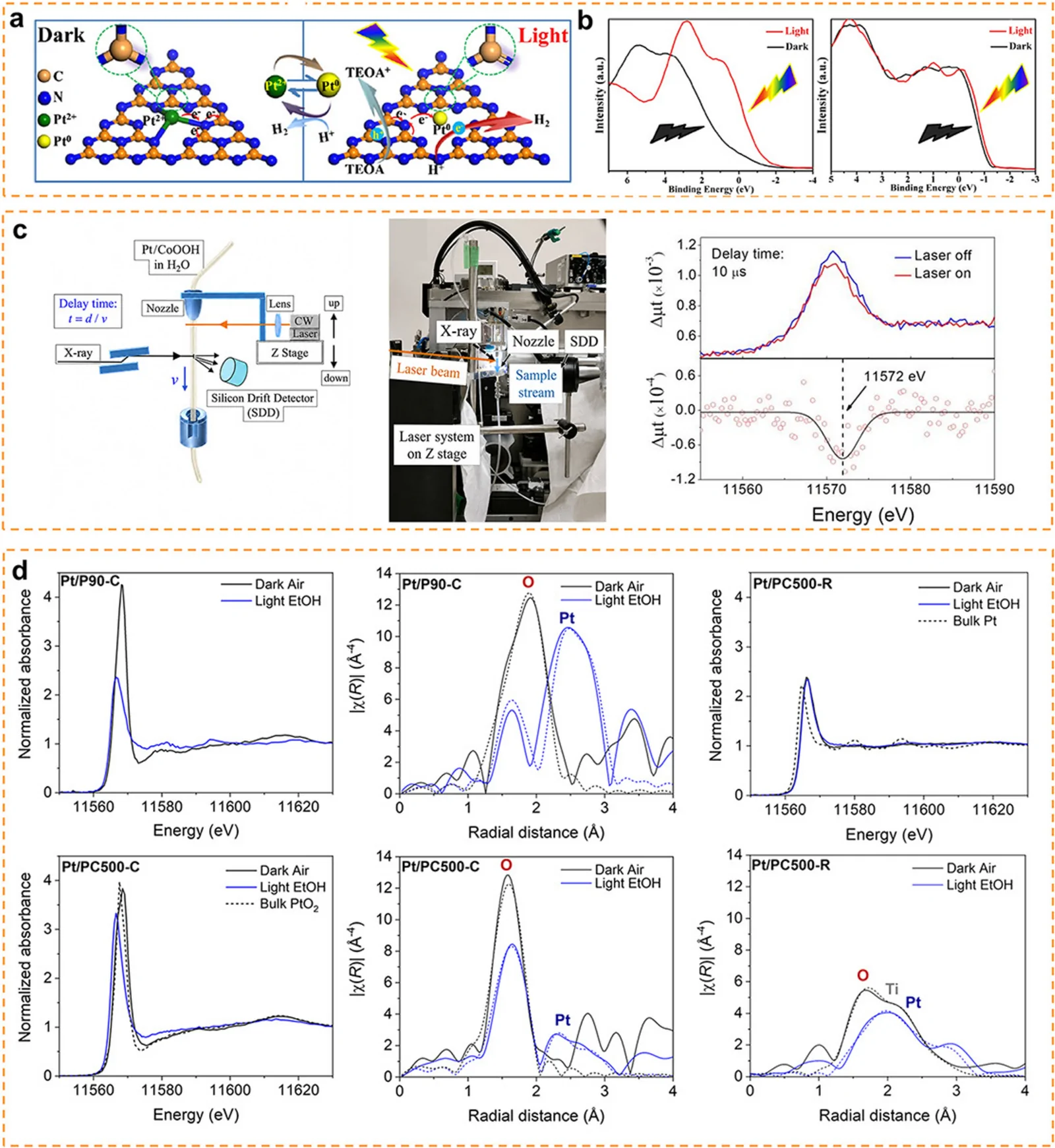
**a** Illustration of charge transfer and bond variation on S-PtC_3_N_4_ catalysts for photocatalytic hydrogen evolution. **b** XPS valence band spectra of S-Pt-C_3_N_4_ and M-Pt-C_3_N_4_ under light and dark conditions [152]. Copyright 2020, Wiley–VCH. **c** Schematic and photo of the laser pump–X-ray probe “X-ray tracking” setup; transient Pt L3-edge signal (Δμt) at a 10 μs delay compares laser off or on, capturing short-time electronic evolution under photo(thermal) fields [211]. Copyright 2025, Oxford University Press. **d** Pt L3-edge XANES and FT-EXAFS (|χ(R)|) for Pt/P90-C, Pt/PC500-C, and Pt/PC500-R in dark air and under light in ethanol; dashed lines are bulk Pt or PtO_2_ references, highlighting changes in Pt–O and Pt–support coordination [212]. Copyright 2020, American Chemical Society

Operando and in situ X-ray methods are well suited to test three questions that matter specifically for noble-metal single atoms: (i) Whether “single atoms” remain isolated under turnover, (ii) how their first-shell coordination evolves, and (iii) whether the electron-accepting state that enables proton reduction is created only under illumination [57, 225, 226]. In practice, the most powerful experimental designs combine an element-specific probe such as XAS or synchronous-illumination XPS with an interface or adsorbate-sensitive technique (e.g., in situ IR/DRIFTS), enabling direct correlation between charge redistribution and bond evolution at the same atomic site [227]. As illustrated by synchronous-illumination XPS and time-resolved X-ray tracking experiments (Fig. 16) [22, 152, 211, 212], light irradiation induces measurable and reversible changes in the Pt electronic state and local bonding environment.

Zhang and coworkers used synchronous-illumination XPS to directly track the working state of single-atom Pt/ C_3_N_4_ during photocatalytic water splitting (Fig. 16a). In the dark, Pt atoms were stabilized by Pt–N coordination. Under light irradiation, Pt–N bond cleavage and Pt⁰ formation were observed, accompanied by C=N bond reconstruction in the C_3_N_4_ framework (Fig. 16b). These changes were absent in Pt nanoparticle-loaded C_3_N_4_, indicating that dynamic Pt–N coordination in single-atom Pt/ C_3_N_4_ promotes interfacial charge transfer and enables Pt⁰ and C_3_N_4_ to serve as reduction and oxidation sites, respectively [152]. Cheng and coworkers applied microsecond-resolved pump-flow-probe XAFS to investigate charge transfer in single-atom Pt/CoOOH photocatalysts [211]. Under laser excitation, a pronounced transient response appears at the Pt L_3_ edge, directly evidencing electron transfer to Pt 5*d* orbitals (Fig. 16c). These results connect ultrafast photoexcitation processes with operando timescales and reveal non-equilibrium electronic states that exist only under illumination.

Piccolo and coworkers studied ultradispersed Pt/TiO_2_ photocatalysts using operando XANES and EXAFS [212]. Light-dependent changes in Pt–O and Pt–support coordination were resolved while atomic dispersion was preserved. This operando approach distinguishes the as-prepared structure from the true working structure that governs hydrogen adsorption and release during photocatalysis. Together, these operando observations demonstrate that atomically dispersed Pt sites are not electronically or structurally static during operation; rather, illumination drives dynamic bond reconfiguration that opens more efficient charge-transfer channels toward the Pt center during photocatalysis, providing a measurable operando electronic consequence that is essential for a noble-metal single-atom narrative (Fig. 16d).

A closely aligned example for a hydrogen-evolution-focused narrative is provided by Pt single atoms anchored on carbon nitride, where SI-XPS combined with SI-DRIFTS captures simultaneous charge migration and Pt–N bond evolution during photocatalytic water splitting [215]. These observations support the view that dynamically evolving N–Pt coordination accelerates forward electron transfer while suppressing backward water formation pathways, a concept that is equally relevant to photothermal photocatalytic PHE, where the interface acts as a dynamic, rate-controlling element that operando tools must validate. Consistently, in situ*/operando* XAFS measurements of Pt single atoms in sulfide photocatalysts such as Cd_0.5_Zn_0.5_S reveal that the Pt coordination environment evolves under illumination, clearly distinguishing the as-prepared structure from the true working configuration [212]. This dynamic restructuring modifies the electronic structure of neighboring sulfur sites, facilitating hydrogen adsorption and release and contributing to enhanced activity [212]. Operando XAFS studies under overall water-splitting conditions further show that oxidic Pt species can form and persist during reaction, underscoring that Pt oxidation state and local geometry may be intrinsically coupled to the photocatalytic cycle rather than fixed material descriptors [228].

Although the studies summarized in Fig. 16 demonstrate the potential of synchronous-illumination XPS, time-resolved X-ray tracking, and operando XANES/EXAFS, systematic time-resolved XAS or XPS under well-controlled photothermal boundary conditions remains scarce. In future studies, X-ray spectral acquisition should be synchronized with light-on/off modulation, local or reaction-zone thermometry, and real-time H_2_ evolution measurements, so that changes in oxidation state, first-shell coordination, and metal–support bonding can be correlated directly with photothermal kinetics. This requirement is particularly important for SAC-based PHE, because reversible valence shifts, coordination breathing, or transient atom–support bond reconfiguration may occur during illumination and heating but disappear after cooling or sample transfer. Therefore, post-reaction HAADF-STEM or EXAFS should be regarded as necessary structural retention evidence, but not as sufficient proof of the true working state under coupled light–heat operation.

### Bridging Ultrafast Charge Transfer with Photothermal Operating Fields

**Fig. 17:**
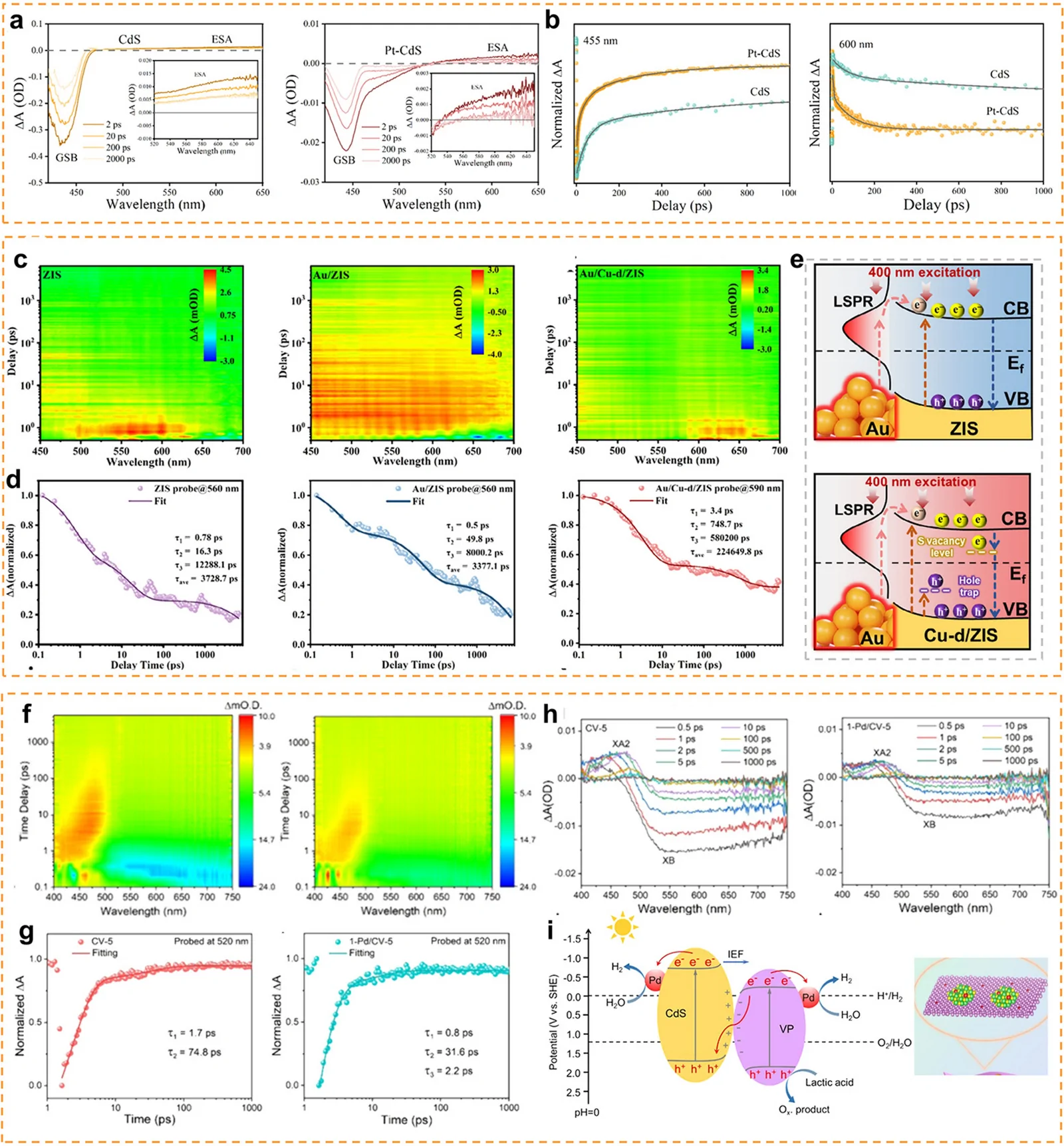
Ultrafast spectroscopy and mechanistic insights into charge dynamics in CdS-based systems. **a**, **b** fs-TA spectra and kinetics comparing CdS and Pt–CdS [125]. Copyright 2023, Elsevier B.V.. **c** Transient absorption spectra and of ZIS, Au/ZIS, and Au/Cu-d/ZIS. **d** Normalized transient absorption kinetics for ZIS, Au/ZIS, and Au/Cu-d/ZIS. **e** Schematic illustration of photogenerated electrons transfer and hot electrons injection [229]. Copyright 2025, Wiley–VCH. **f** 2D TA maps for CV-5 and Pd/CV-5.** g** TA spectra. **h** Kinetics and fitting. **i** Band alignment and interfacial electric field-driven Z-scheme pathway [55]. Copyright 2024, American Chemical Society

To connect operando structural observations with catalytic function, ultrafast spectroscopy provides a critical kinetic bridge, because the apparent superiority of many single-atom cocatalysts originates from their ability to extract photogenerated electrons on sub-nanosecond timescales, well before bulk recombination or thermal equilibration occurs [230, 231]. As summarized in Fig. 17, transient absorption measurements directly visualize how single atoms, defects, and interfacial fields reshape carrier pathways and lifetimes across representative photocatalyst platforms.

Xiang and coworkers anchored platinum (Pt) single atoms on CdS quantum dots, building an EMSI interface via one-pot solvothermal deposition and ligand exchange (Fig. 17a) [125]. Femtosecond transient absorption spectroscopy (fs-TA) quantified an ultrafast CdS to Pt electron transfer of ~ 1.7 ps (Fig. 17b), demonstrating that isolated Pt sites intercept electrons prior to recombination [125]. This kinetic gain yielded a ~ 70-fold increase in PHE in water under illumination, reaching 2.12 mmol h^−1^ g^−1^ under their conditions [125]. The same catalyst also couples hydrogen evolution with selective oxidation of 2-thiophene methanol to 2-thiophenecarboxaldehyde, enabling dual carrier utilization [125].

Ruan et al. employed femtosecond transient absorption spectroscopy to reveal the carrier-transfer behavior in Au/Cu-d/ZIS (Fig. 17c-e) [229]. Compared with pristine ZIS and Au/ZIS, Au/Cu-d/ZIS shows a much slower decay of transient signals, indicating prolonged carrier lifetime and suppressed recombination. This improvement is attributed to the synergistic effect of Cu-induced bulk carrier trapping and Au-driven plasmonic hot-electron accumulation on the surface, which enables more efficient charge separation for photocatalytic H_2_ evolution (Fig. 17e).

For polymer photocatalysts, Durrant and coworkers used transient absorption and operando optical spectroscopy to show that residual palladium clusters left from polymer synthesis can behave as real electron sinks on the femtosecond-to-nanosecond timescale [192]. In fluorene–benzothiadiazole copolymer NPs, palladium levels above ~ 1000 ppm enable ultrafast exciton quenching at Pd clusters that outcompete reductive quenching by sacrificial donors, whereas lowering Pd shifts long-lived electrons onto the polymer [232]. In a dibenzo[b,d]thiophene sulfone polymer, long-lived electrons remain largely polymer bound even at very high Pd loading, implying slow electron delivery to the metal and a kinetic bottleneck for hydrogen evolution [232]. Consistent with this cautionary message, Fig. 17f-h compares ultrafast signatures and fitted kinetics for organic systems with and without Pd (Fig. 17f-h) and summarizes the corresponding band and field-driven pathway picture (Fig. 17i), emphasizing that “metal-assisted” kinetics depends critically on how the metal is speciated and electronically coupled [55, 232].

Photothermal-enhanced PHE adds another dimension that operando studies must explicitly control, namely, temperature [233]. Qian et al. systematically separated the roles of photon flux, wavelength, and temperature on interfacial electron-transfer kinetics using TiO_2_ as a model and then used Sn-doped TiO_2_ to suppress temperature-driven charge leakage in a fixed-bed configuration while achieving high H_2_ productivity at elevated temperature [233]. This directly aligns with the photothermal photocatalysis theme because it shows that heat can reshape the semiconductor–cocatalyst barrier and the effective charge-transfer duration, rather than merely accelerating surface kinetics in an Arrhenius sense [233].

## Summary and Outlook

Photothermal-enhanced PHE on noble metal single-atom catalysts has moved beyond proof of the concept activity gains. It now demands translation-oriented design that treats catalysts and reactors as a coupled system. At the atomic scale, site structure governs charge capture, intermediate binding, and resistance to reconstruction. At larger scales, photon transport, bubble dynamics, and heat and mass transfer define the effective boundary conditions that the site experiences. This mismatch between intrinsic descriptors and reactor level constraints explains why activity trends often fail to translate when catalyst mass increases, water matrices become complex, or illumination becomes non-ideal.

High atomic efficiency and overall light–heat–charge coupling efficiency are not intrinsically contradictory in SAC-based photothermal PHE, but they must be optimized together. Isolated noble-metal atoms can maximize metal utilization and provide well-defined electron extraction or HER-active sites, but their contribution to system efficiency depends on whether they are spatially matched with light-absorbing regions, photothermal domains, charge-transfer interfaces, and accessible reaction pathways. Therefore, the design target should not be the lowest possible noble-metal loading alone, but an optimized balance among atomic-site density, photon absorption, local heat generation, interfacial charge delivery, heat dissipation, and mass transport.

**Fig. 18:**
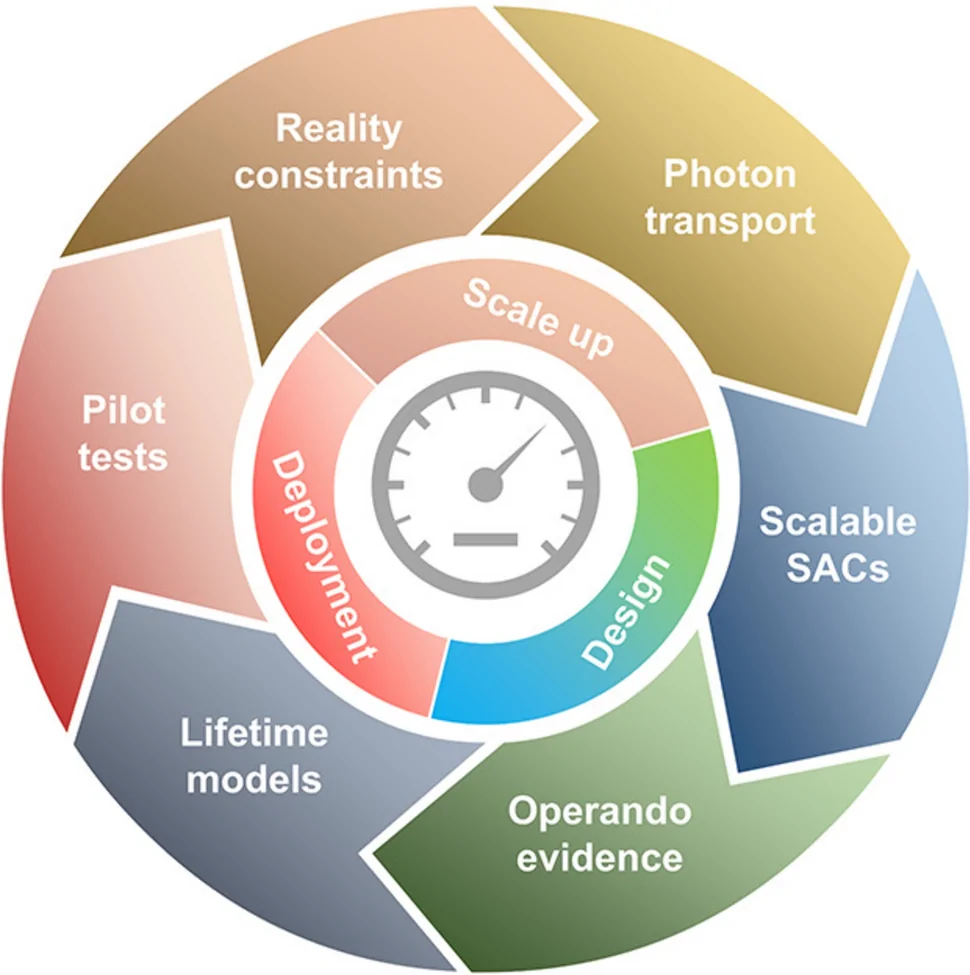
Iterative translation roadmap for photothermal-enhanced PHE on noble metal single-atom catalysts. The outer ring connects six recurring steps, reality constraints, photon transport, scalable SACs, operando evidence, lifetime models, and pilot tests. The inner ring highlights the coupled themes of scale up, deployment, and design

Figure 18 summarizes a practical translation loop that connects discovery to deployment. The loop links reality constraints, photon transport, scalable SACs, operando evidence, lifetime models, and pilot tests. It emphasizes feedback rather than a linear pipeline. Reactor optics and transport set the test conditions. Operando evidence identifies which motifs remain active and stable under those conditions. Lifetime models convert early signatures into deployment relevant predictions. Pilot tests close the loop and expose constraints that guide the next design cycle.

### Stability and Atomic Dispersion Under Photothermal Operation

A central challenge for photothermal-enhanced noble-metal SACs systems is maintaining atomic dispersion and coordination integrity under sustained illumination and elevated temperature. The same factors that boost rates, such as localized heating, stronger interfacial electric fields, and changes in interfacial water structure, can increase atomic mobility and drive restructuring. Sintering via atom migration and coalescence is a primary degradation pathway, and temperature-dependent studies show sintering accelerates sharply once moderate thermal thresholds are crossed, enabling hour-scale diffusion that is negligible at room temperature [234, 235].

Support chemistry adds distinct failure modes. On sulfides, photocorrosion and oxidative dissolution driven by unconsumed holes can destabilize the lattice even with scavengers, while persistent illumination can open new migration pathways through non-equilibrium charges and reactive surface species [236]. On carbides and carbon-based supports, surface oxidation, reconstruction, and compositional shifts such as carburization or decarburization can weaken anchoring motifs. On oxides, oxygen-vacancy healing, cation leaching, and surface plane reconstruction can change both the geometry and electronic structure of the anchored sites.

Long-duration validation remains limited: Many reports show 10–50h of apparent stability, whereas practical duty cycles often require beyond 10,000h with minimal loss [237]. Post-reaction microscopy and X-ray absorption frequently detect sintering or support reconstruction even when short-term activity decay is modest. Extended operando tracking is therefore crucial to separate gradual coalescence from reversible coordination or electronic shifts and from irreversible support degradation. Machine learning is starting to flag instability risk from composition, coordination, and support descriptors, but converting predictions into robust, transferable durability rules is still unresolved.

### Mechanism Complexity and Intermediate Identification

Mechanisms in photothermal-enhanced PHE are still not fully resolved. Even for classical Pt cocatalysts on oxides, key uncertainties remain, including whether transient subsurface hydrogen forms, how significant hydrogen spillover is between Pt and the semiconductor, and how local heating reshapes these routes [238]. For noble-metal single-atom catalysts, interpretation is harder because atomic coordination introduces site heterogeneity: Different facets, defects, and coordination pockets can host electronically distinct single-atom motifs within nominally similar samples [215, 239]. Photothermal operation adds another layer, as dynamic restructuring, local strain, symmetry breaking, and interfacial water organization can shift the working motif and alter intermediate adsorption [240, 241]. Adaptive Pd SACs also show that isolated sites can switch reactivity under different reaction environments [242]. Operando spectroscopy has improved intermediate detection, but temporal and spatial resolution remain limiting. X-ray absorption spectroscopy averages over all metal atoms, potentially obscuring heterogeneity and minority active motifs. Surface-sensitive methods such as diffuse reflectance infrared Fourier transform spectroscopy often require specialized reactors and may not fully represent bulk suspension behavior. High-pressure operando transmission electron microscopy is advancing but remains technically demanding and susceptible to beam effects. Computation, including DFT and molecular dynamics, offers atomic-scale insight but still needs rigorous experimental validation, especially for temperature effects, interfacial water structure, and realistic electrostatics. An emerging frontier is linking ultrafast spectroscopy to mechanistic assignments. Transient absorption can quantify charge-transfer timescales but usually cannot identify which bonds form or which intermediates dominate. Combining time-resolved methods with operando X-ray absorption spectroscopy or infrared measurements could provide much richer mechanistic detail, and developing molecular labels or minimally invasive probes to track key intermediates without disturbing the interface remains a major challenge.

### Scale-Up Constraints: Photons, Transport, and Manufacturing

Scale-up is governed by photon delivery, coupled transport, and manufacturable single-atom catalyst production (Fig. 18). Laboratory photocatalytic tests are usually performed in small batch reactors under simplified conditions, with milligram-scale catalysts, short optical paths, stable illumination, vigorous stirring, and often sacrificial agents. Practical systems, in contrast, must operate under variable sunlight, complex water matrices, larger illuminated areas, continuous gas release, and stricter requirements for safety, durability, heat management, and routine maintenance [2–5, 10, 11]. This creates a quantitative scale gap between material-level PHE demonstrations and reactor-level hydrogen production. Representative system-level studies provide useful benchmarks: Particulate photocatalyst sheets have achieved STH efficiencies exceeding 1%, while outdoor particulate panel systems have been demonstrated at the 100 m^2^ scale with a reported maximum STH efficiency of 0.76% [6, 8]. More recent scalable systems have also emphasized separated H_2_/O_2_ production and practical gas handling as essential reactor-level requirements [9]. These values are often much lower than mass-normalized rates reported in material screening, but they are more relevant to deployment because they include photon delivery, gas collection, reactor geometry, and system-level losses [3–5, 10].

Photon utilization becomes increasingly limiting as reactor dimensions increase. In slurry reactors, scattering and optical attenuation cause photon flux to decrease with depth, so only part of the catalyst population operates near the activity measured in small vials [3, 10]. For photothermal PHE, this optical limitation is coupled to heat dissipation. Increasing catalyst loading or optical density can enhance photon capture and local photothermal conversion near the illuminated window, but it also shortens the effective penetration depth, creates under-illuminated regions, and intensifies local temperature gradients [10, 35, 63]. Dilute suspensions improve light distribution and heat dissipation, but reduce the volumetric density of active sites and weaken photothermal heating [10]. Reactor optimization, therefore, requires a coupled balance among catalyst concentration, optical path length, irradiation geometry, mixing intensity, and heat removal, rather than simply increasing catalyst dosage [3–5, 10].

Gas bubble formation and release are also central to reactor-level operation. As reaction rate and illuminated area increase, H_2_ bubbles can cover active sites, scatter or shield incident light, alter the local temperature field, and increase near-surface mass-transfer resistance [35, 71]. At the same time, bubble detachment may induce local convection, renew the catalyst–liquid interface, and improve heat dissipation [35, 63]. Bubble behavior, therefore, couple photon transport, photothermal gradients, and mass transfer, especially in dense slurries, poorly wetted powders, or immobilized films. Floatable or immobilized platforms can improve optical delivery, reactant supply, and product separation, but they introduce additional constraints including fabrication complexity, module cost, long-term adhesion, interfacial contact resistance, and gas-release pathways [70]. Thus, reactor-level studies should report not only mass-normalized H_2_ evolution rates, but also illuminated area, optical path length, catalyst loading or slurry concentration, temperature distribution, gas-release behavior, mixing conditions, and area- or energy-normalized hydrogen productivity [5, 10].

Manufacturing and practical interface constraints must be considered alongside transport constraints, especially because recent high-current–density water-electrolysis studies further highlight the importance of hierarchical interface engineering under realistic hydrogen production conditions [243]. Techno-economic analyses typically identify catalyst manufacturing cost and photoreactor capital cost as major levers for solar hydrogen production [10]. This is especially relevant for Pt-, Ru-, and Ir-based SACs, where noble-metal loading must be minimized without sacrificing site accessibility, long-term stability, or charge-transfer efficiency. Scalable synthesis should, therefore, be evaluated not only by whether isolated atoms can be formed, but also by batch-to-batch reproducibility, support cost, precursor utilization, wastewater generation, and preservation of atomic dispersion after shaping into slurries, sheets, coatings, or modules.

### Efficiency Metrics and Evaluation Boundaries for Photothermal-Enhanced PHE

Efficiency evaluation in photothermal-enhanced PHE should be matched to the reaction pathway, optical boundary condition, and intended application scenario. A single H_2_ evolution rate is insufficient to describe the solar conversion capacity of a light–heat–charge coupled system, because the apparent rate can be strongly affected by catalyst mass, irradiation area, light intensity, spectral distribution, absorbed power, reaction temperature, sacrificial reagent, and gas collection method [2, 3, 10]. Therefore, efficiency metrics such as AQY, AQE, STH, and LHCE should not be used interchangeably without clarifying their assumptions and applicable boundaries [5, 10].

AQY or AQE is the most practical comparative metric for many studies summarized in this Review, because most reported PHE-SAC systems are evaluated under wavelength-specific or filtered light HER conditions [18, 19, 33]. AQY/AQE is useful for comparing photon-to-H_2_ conversion at a defined wavelength and for identifying whether a catalyst can utilize specific absorption bands or plasmonic resonances [37]. However, AQY/AQE does not directly represent full-spectrum solar conversion efficiency, especially when the test is performed under narrow-band irradiation, high light intensity, or in the presence of sacrificial donors [5, 10]. In photothermal-enhanced systems, AQY/AQE should also be interpreted carefully because absorbed photons may contribute simultaneously to carrier generation and local heating. Therefore, AQY/AQE should be reported together with wavelength, bandwidth or filter type, irradiance, illuminated area, catalyst amount, reaction temperature, and whether the value is based on incident or absorbed photons [10].

By contrast, STH is a higher-level system metric for practical sacrificial-agent-free solar water splitting under standard solar illumination [3, 10]. However, STH should be applied only when the input solar power, illuminated area, product stoichiometry, and absence of sacrificial reagents are clearly defined [5, 10]. For sacrificial-agent-assisted HER, a high H_2_ production rate or high AQY should not be described as STH for overall water splitting, because the oxidation half-reaction is replaced by donor consumption [3]. In conventional overall water splitting, STH evaluation requires quantitative H_2_ and O_2_ detection, near 2:1 stoichiometry, gas-tightness verification, and exclusion of external bias or hidden chemical energy input [5, 10]. For alternative pure water pathways such as H_2_/H_2_O_2_ production, the efficiency calculation should explicitly account for the actual oxidation product rather than assuming four-electron O_2_ evolution [128].

Light-Harvesting Conversion Efficiency (LHCE) and absorbed light-based descriptors are helpful for separating optical absorption from catalytic utilization. These descriptors are especially useful in photothermal-enhanced PHE because a large fraction of absorbed light may be dissipated as heat instead of being converted into long-lived carriers [33]. However, high light absorption or high LHCE alone does not prove efficient H_2_ generation, because absorbed energy may be lost through recombination, bulk heating, nonproductive heat dissipation, or mass-transfer limitations [37, 179]. Thus, absorbed light-based metrics should be coupled with charge-transfer efficiency, local or reaction-zone temperature, and product formation rate [37, 39]. For photothermal systems, absorbed power-normalized H_2_ evolution rate can provide an additional comparison, but it should be reported with the thermal boundary condition and temperature measurement method [179].

A more appropriate evaluation framework for photothermal-enhanced PHE should combine photon, thermal, catalytic, and stability descriptors. At minimum, future studies should report the H_2_ evolution rate, AQY/AQE or STH when applicable, catalyst amount, irradiance, spectral distribution, illuminated area, absorbed photon flux or absorbed power, reaction-zone temperature, sacrificial reagent or pure water condition, product stoichiometry, and long-term stability [37, 179]. For SAC-based systems, these metrics should further be linked to metal loading, site density, TOF calculation basis, and post-reaction or operando verification of atomic dispersion [16]. Such an integrated index system can better reflect whether the system truly improves solar-to-hydrogen conversion, rather than merely increasing apparent activity under favorable optical or thermal conditions.

In this context, the goal of efficiency evaluation is not to identify one universal number, but to define a transparent set of descriptors that matches the mechanism and application scenario. For mechanistic studies, AQY/AQE, absorbed power-normalized activity, local temperature, and kinetic descriptors are most useful [37]. For practical water splitting, STH, H_2_/O_2_ stoichiometry, gas separation safety, and durability under realistic solar fluctuation become more important [2, 3, 10]. For photothermal SACs, metal utilization and TOF remain valuable, but they should be interpreted together with photon absorption, heat localization, charge extraction, and mass transport [16]. This multi-index evaluation is essential for judging the true solar conversion capacity of photothermal-enhanced PHE systems.

### Deployment Gates: Durability, Safety, and Renewable Integration

Deployment is limited by long-duration stability and safe product handling under realistic outdoor conditions. Many laboratory demonstrations still rely on sacrificial electron donors to simplify chemistry and boost apparent rates. Sacrificial-donor-assisted H_2_ evolution should, therefore, be distinguished from overall water splitting. In many PHE-SAC studies, sacrificial agents such as TEOA, methanol, lactic acid, ascorbic acid, Na_2_S/Na_2_SO_3_, or alcohol substrates are used to consume photogenerated holes and increase the apparent H_2_ production rate. These systems are useful for probing electron extraction, proton-reduction kinetics, and support–SAC charge transfer, but they demonstrate a HER half-reaction rather than closed water redox chemistry.

A convincing claim of conventional overall water splitting requires quantitative detection of both H_2_ and O_2_, preferably by calibrated online GC or MS, with a near 2:1 H_2_/O_2_ stoichiometric ratio, stable time-dependent production, gas-tightness and blank controls, and verification of product origin using isotope labeling such as H_2_^1^⁸O or D_2_O when feasible [2–5]. Among SAC-related systems, only a limited number of combinations have been reported under sacrificial-agent-free water-redox conditions with simultaneous product analysis. For conventional H_2_/O_2_ overall water splitting, Ir/BiVO_4_-based Z-scheme systems with Ir/IrO_2_ motifs provide a representative example, where stoichiometric H_2_ and O_2_ evolution was reported under illumination [158]. Ru single atoms/RuOx clusters on ZnIn_2_S_4_ have also been reported for photocatalytic pure water splitting without sacrificial agents [159], but such systems should be evaluated according to whether simultaneous H_2_/O_2_ quantification, near-2:1 stoichiometry, and long-term stability were demonstrated. By contrast, Pd single atoms coupled with sulfur vacancies on ZnIn_2_S_4_ represent a different sacrificial-agent-free water-redox route, producing H_2_ and H_2_O_2_ from pure water rather than H_2_ and O_2_ [128]. This example highlights that “pure-water” operation should not automatically be equated with conventional H_2_/O_2_ overall water splitting; the oxidative product and product stoichiometry must be explicitly identified. Many high rate Pt- or Pd-based sulfide systems still rely on TEOA, lactic acid, benzyl alcohol, or alcohol donors and should, therefore, be interpreted as HER half-reaction benchmarks rather than true overall water-splitting systems [127, 129, 131]. Thus, SAC/support combinations reported under sacrificial conditions should not be directly compared with pure water-splitting systems unless the reaction medium, product stoichiometry, gas analysis, and isotope or control evidence are clearly specified.

Accordingly, sacrificial-free overall water splitting remains challenging for most particulate systems. It often delivers lower efficiency than half-reaction tests and can generate mixed H_2_/O_2_ streams, increasing safety and separation demands. Deployment, therefore, requires robust gas management, separation strategies, and materials that tolerate pressurization and corrosion. Beyond gas handling, diurnal cycling and real-world fluctuations in solar irradiation should also be regarded as critical system-level constraints for photothermal hydrogen production. Under outdoor operation, changes in solar intensity, spectral distribution, ambient temperature, wind, and weather can repeatedly alter local temperature fields, gas-evolution rates, bubble release, and mass transport. For SAC-based photothermal PHE, such heating–cooling cycles may also influence atomic-site stability, coordination evolution, and metal–support interactions. Therefore, large-scale deployment requires reactor and system designs that tolerate intermittent light input and variable gas production, including thermal buffering, robust gas management, hydrogen storage, hybrid operation with other renewable energy sources, and electrical buffering.

### Design Accelerators: Operando Anchored Discovery, Predictive Models, and Multi-site Architectures

Next-generation progress will be fastest when operando observables define predictive rules for activity and lifetime, and when multisite designs are pursued only with tractable synthesis and verification. The design space for photothermal single-atom systems is large. It includes metal identity, support chemistry, anchoring motifs, dopants, defect density, morphology, framework-confined microenvironments, and multi-site architectures [244, 245]. Beyond conventional impregnation, photodeposition, pyrolysis, defect anchoring, and framework-confined strategies, high-resolution photopolymerization 3D printing offers an emerging route to structured SACs that couple atomic-site dispersion with controllable geometry and enhanced mass transport [246]. Chiral SACs further broaden this design space through asymmetric confinement and local stereochemical control [247]. High-throughput computation can prioritize candidates. Predictive reliability decreases when finite temperature dynamics, aqueous ions, and reconstruction pathways dominate. Multiscale modeling is valuable because it links atomic-scale processes to continuum heat and mass transport that governs reactor level behavior. Machine learning can shorten iteration cycles when datasets include synthesis variables, operando descriptors, and stability outcomes. Useful targets include forecasting lifetime from early time signatures and predicting tolerance to impurities in realistic water matrices. Many models still face limits in interpretability and actionability. Hybrid strategies that embed mechanistic constraints and physically meaningful descriptors are more likely to guide synthesis and scale-up decisions. Multi-site architectures are an additional opportunity. Dual atom motifs and atom cluster combinations can distribute elementary steps across neighboring centers. They may enable selectivity and efficiency beyond monometallic sites. Key barriers include control over site spacing and coordination and unambiguous operando identification of coupled dynamics. Hybrid inorganic biological systems can offer high selectivity but require robust immobilization and efficient interfacial electron transfer under photothermal stress.

In summary, the field would benefit from a unified focus on several priorities. First, durability verification under extended operation is essential. Multi-thousand-hour stability tests under realistic light, water, and catalyst conditions, combined with operando structural monitoring, are needed to identify dominant failure modes and develop countermeasures. Second, rigorous mechanistic studies are required. Integrated efforts combining temperature-controlled kinetics, operando multi-probe characterization, and validated computational modeling will clarify how thermal and photochemical effects couple at the atomic scale. Third, synthesis scalability must be prioritized. High-yield, low-temperature, and environmentally benign routes for noble-metal SACs, along with credible pathways to reduce or replace noble metals where performance allows, are critical for deployment. Fourth, advanced photoreactor engineering is needed. Reactor designs optimized for light distribution, heat management, and mass transfer should be validated at scales approaching pilot plants to bridge laboratory and industrial implementation. Fifth, standardized testing and mechanistic frameworks should be adopted. Consensus protocols for reporting activity, separating thermal from photochemical contributions, and quantifying stability will enable transparent comparison across the literature. Sixth, integration with emerging applications deserves greater attention. Coupled redox schemes, hybrid photovoltaic–photocatalytic configurations, and bio-inspired architectures can improve practical viability and expand application scope.
